# The assessment of language restrictions in abstracts of systematic reviews in dentistry: A meta-research study

**DOI:** 10.1371/journal.pone.0323176

**Published:** 2025-05-20

**Authors:** Tatjana Lörscher, Naichuan Su, Clovis Mariano Faggion

**Affiliations:** 1 Department of Periodontology and Operative Dentistry, University Hospital Münster, Münster, Germany; 2 Department of Oral Public Health, Academic Centre for Dentistry Amsterdam (ACTA), University of Amsterdam and Vrije Universiteit Amsterdam, Amsterdam, The Netherlands; 3 Department of Periodontology and Operative Dentistry, University Hospital Münster, Münster, Germany; Danube Private University, AUSTRIA

## Abstract

**Background:**

The adequate interpretation of findings in systematic reviews can be affected by the lack of information on the language of the examined studies. The study sought to assess the reported information on restrictions set on the language of primary studies examined in systematic reviews published in dentistry. The study also investigated associations between the characteristics of the systematic reviews and language restrictions.

**Methods:**

A comprehensive search was conducted in the Web of Science database for systematic reviews in the field of dentistry. Abstracts published from the inception of the database up to 24 February 2023 were included and relevant information was extracted. Only abstracts published in English were included. Logistic regression analyses were performed to examine the association between the characteristics of the systematic reviews and the presence of language restrictions. Additionally, a random sample of 9.2% of the full texts was reviewed to identify differences in the reporting of language restrictions between the abstract and the full texts.

**Results:**

A total of 3922 abstracts were initially retrieved, and 3465 abstracts were included in the analysis based on the eligibility criteria. Approximately 79% (2739) did not report any language information. Only 7% (238) of the abstracts declared no language restrictions in the primary studies selected. Meta-analysis conducted, journal type, reporting of primary study design, actual number of words in abstracts and the country and continent of first authors affiliation were statistically significantly associated with language restrictions of the systematic reviews. However, the absence of information about language restriction appears to be a poor indicator of reporting or not language restriction in the full-text of the article.

**Conclusions:**

Abstracts of systematic reviews in dentistry frequently underreport language restrictions applied to the primary studies examined. Various characteristics of systematic reviews are significantly associated with these restrictions, highlighting inconsistencies in reporting practices.

## Introduction

An abstract should receive the same attention (or even more) as the full text of a scientific article regarding the completeness of the reporting. The relevance and value of a complete abstract have likely risen with the increasingly large amount of literature that is being regularly published. It has been estimated that nearly 80 systematic reviews are indexed in PubMed every day [1]. Hence, for busy readers, such as clinicians, it is challenging to remain updated about a specific speciality by reading the full text of all relevant articles. Some evidence suggests that many clinicians, for example, likely only read the abstracts of many scientific articles to stay current (with further reading being more selective). A study assessing the reading habits of internists with and without epidemiological training reported that they read only the abstracts of up to 63% of the articles from the nine medical journals they read regularly [2].

Several guidelines have been published to help researchers report their studies, and a guideline for reporting abstracts of systematic reviews has also been published [3]. The PRISMA 2020 extension for Abstracts suggests 12 items that should be reported in any abstract of systematic reviews. This does not include specific guidance on how to report information on the language of the articles that are examined in a review or on how this has been applied as part of the search strategies or eligibility criteria [4].

One can consider that some sort of language bias [5] may occur when there is a restriction in the search regarding the number of languages chosen or the particular languages selected. It is important to the reader of the abstract of a systematic review that the information provided is detailed enough to allow an adequate interpretation of the methodology and results.

The aims of this meta-research study were twofold: (a) to investigate the reporting of restrictions set in dental systematic review abstracts regarding the languages of the examined primary studies and (b) to investigate potential associations between the characteristics of the systematic reviews and language restrictions.

## Materials and methods

### Study design

This was a cross-sectional study of systematic reviews. The abstracts were analyzed plus a random sample of 9.2% of the full texts. Some passages in this paper use the terms “abstract” and “systematic review” interchangeably to facilitate understanding.

### Eligibility criteria

#### Inclusion criteria.

Abstracts of systematic reviews, with or without meta-analyses of primary studies, in the field of dentistry were included in the analysis. To be included, a systematic review must have oral health as its primary focus, excluding those centered on related or peripheral fields. Protocols of systematic reviews were also included. Other types of reviews considered systematic such as, for example, scoping reviews were included if the key-word “systematic” was reported in the title of the study. No restrictions were applied regarding the species examined or the type of research. Only abstracts published in English were included.

#### Exclusion criteria.

Abstracts of systematic reviews from other medical disciplines that did not focus on oral health topics were excluded. Additionally, other study designs, including primary studies, comments, and statements, as well as secondary research studies such as reviews of reviews (commonly referred to as umbrella reviews), were also excluded. Studies that identified themselves as systematic reviews but included both reviews and primary studies were excluded. Additionally, if it was unclear from the abstract whether primary studies were included, the study was also excluded to maintain consistency in the selection process.

### Search strategy

A search was conducted in the Web of Science (WoS) database from its inception up to 24 February 2023.

The search used the Medical Subject Headings (MeSH) terms from the United States National Library of Medicine, together with Boolean operators (see supplementary file).

### Data selection

The documents retrieved from the database were exported to an Excel worksheet for data selection and extraction. Titles and abstracts were assessed for eligibility according to the predefined inclusion criteria. Abstracts that did not meet the criteria were excluded, and the specific reason for exclusion was recorded.

Two assessors (TL and CMF) selected a sample of 10% of the eligible studies and made independent inclusion decisions (achieving good agreement, 90 per cent), with the remainder selected by one reviewer (TL) [6].

### Data extraction

The following data were extracted from the abstracts: (a) language restriction (no language restriction, restricted to some languages, restricted to English only or no information about language); (b) topic of the review (based on the classification of dental specialties by the National Commission on Recognition of Dental Specialties and Certifying Boards [NCRDSCB] of the American Dental Association) [7]; (c) structure of abstract (structured or not structured); (d) type of systematic review (intervention or non- intervention); (e) meta-analysis conducted (yes or no); (f) journal type (dental or other); (g) reporting of primary study design (reported or not reported); (h) word limit of abstract (≤250 words or >250 words) based on the information reported in the author guidelines of the journals; and (h`) actual number of words in the abstracts. Additionally, information automatically retrieved from the Web of Science database was compiled, including: (i) author names; (j) article title; (k) author affiliations; (l) number of citations (WoS Core); (m) publisher; (n) country of the affiliation of the first author (developing or developed); and (o) continents (North America, South America, Europe, Asia, Africa or Oceania).

Two assessors (TL and CMF) extracted a sample of 10% of the eligible studies and achieved good agreement on the information accuracy (85 per cent), with the remainder extracted by one reviewer (TL) [6].

### Data analysis

Descriptive statistics were used to summarise the characteristics of the included systematic reviews (i.e., proportions were used for categorical variables; median and interquartile range [IQR] were used for the continuous variables [i.e., actual number of words in abstract and number of citations (WoS Core)], because those two variables were not normally distributed based on Kolmogorov-Smirnov test). To assess the association between the characteristics of the systematic reviews (independent variables) and the language restrictions (outcome variable), multinominal logistic regression analysis was used because the outcome variable included four categories (i.e., no language restriction [reference category], restricted to some languages, restricted to English only and no information). To assess the association between the characteristics of the systematic reviews (independent variables) and the number of languages included (outcome variable), binary logistic regression analysis was used because the outcome variable was dichotomised (i.e., only one language [reference category] and more than one language). In each logistic regression analysis, univariable analysis was first performed to separately assess the association of each independent variable with the outcome variables. Second, a multivariable regression analysis was performed for each outcome variable. In the multivariable regression analyses, only the independent variables with a p-value < .05 in the univariable analyses were included. SPSS Statistics for Windows (version 28.0; IBM, Armonk, NY, USA) was used to perform the statistical analyses.

### Assessment of language restriction between abstracts and full texts

To understand potential differences in reporting of language restriction between abstracts and full texts, a random sample of full texts of these abstracts (9.2%) were scrutinized for language restriction.

## Results

### Study selection

**Fig 1:**
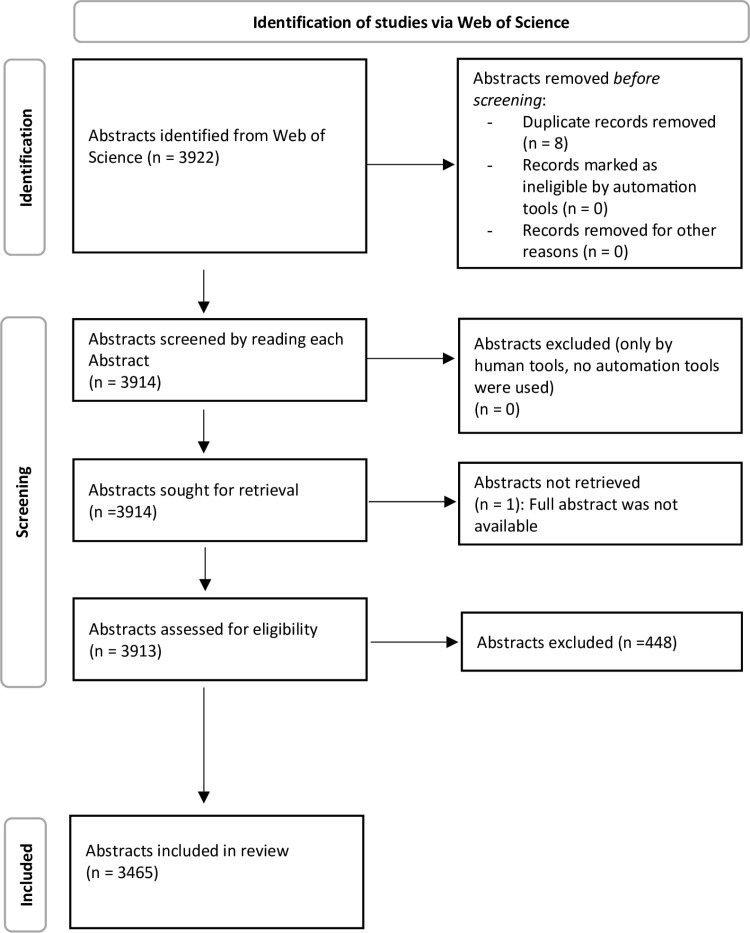
Flowchart of the search and selection process of abstracts of systematic reviews published in dentistry.

A total of 3922 abstracts were initially included. 8 duplicate records have been removed and one that wasn’t fully available. After assessing the abstracts, 448 were excluded because they did not meet the eligibility criteria. Therefore, information from 3465 abstracts of systematic reviews was extracted and included in this study. Detailed information on the selection process is reported in Fig 1.

From the 3465 entries in our database, a sample 347 was selected for full text assessment. This sample was selected randomly using the online Research Randomizer Software (version 4.0; Lancester, Pennsylvania, USA) [8]. Of the 347 selected publications only 319 were accessible for analysis (9.2% of full dataset).

### Study characteristics

Most abstracts (n = 2739, 79%) did not report any information about the language of the studies or related to the search strategies, while the remaining 726 (21%) abstracts reported information about a language restriction. Among the 726 abstracts, the number of abstracts with no language restriction, restriction to some languages and restriction to English only was 238, 68 and 420, respectively. For the group of abstracts that reported the number of included languages (n = 726), 422 (58%) focused on only one language. The main characteristics of the included systematic reviews are presented in Table 1.

**Table 1:** General characteristics of the included systematic reviews and their distributions over language restrictions (N = 3465) and number of included languages (N = 726) based on the abstracts.

	Language restrictions (N = 3465)					Number of included languages (N = 726)		
	Total (N = 3465)	No language restriction (N = 238)	Restricted to some languages (N = 68)	Restricted to English only (N = 420)	No information (N = 2739)	Total (N = 726)	Only 1 language (N = 422)	>1 language (N = 304)
**Topics of the reviews**								
Dental public health	326 (9.4%)	15 (4.6%)	9 (2.8%)	41 (12.6%)	261 (80.1%)	65 (9.0%)	41 (63.1%)	24 (36.9%)
Endodontics	171 (4.9%)	12 (7.0%)	2 (1.2%)	22 (12.9%)	135 (78.9%)	36 (5.0%)	22(61.1%)	14 (38.9%)
Oral and maxillofacial radiology	27 (0.8%)	1 (3.7%)	0 (0.0%)	6 (22.2%)	20 (74.1%)	7 (1.0%)	6 (85.7%)	1 (14.3%)
Oral and maxillofacial surgery	332 (9.6%)	12 (3.6%)	4 (1.2%)	32 (9.6%)	284 (85.5%)	48 (6.6%)	32 (66.7%)	16 (33.3%)
Oral medicine	61 (1.8%)	5 (8.2%)	3 (4.9%)	7 (11.5%)	46 (75.4%)	15 (2.1%)	7 (46.7%)	8 (53.3%)
Orofacial pain	138 (4.0%)	13 (9.4%)	5 (3.6%)	23 (16.7%)	97 (70.3%)	41 (5.6%)	24 (58.5%)	17 (41.5%)
Orthodontics and dentofacial orthopaedics	349 (10.1%)	49 (14.0%)	6 (1.7%)	32 (9.2%)	262 (75.1%)	87 (12.0%)	32 (36.8%)	55 (63.2%)
Pediatric dentistry	122 (3.5%)	10 (8.2%)	1 (0.8%)	14 (11.5%)	97 (79.5%)	25 (3.4%)	14 (56.0%)	11 (44.0%)
Periodontics	461 (13.3%)	23 (5.0%)	7 (1.5%)	46 (10.0%)	385 (83.5%)	76 (10.5%)	46 (60.5%)	30 (39.5%)
Prosthodontics	1165 (33.6%)	78 (6.7%)	24 (2.1%)	155 (13.3%)	908 (77.9%)	257 (35.4%)	156 (60.7%)	101 (39.3%)
Others	313 (9.0%)	20 (6.4%)	7 (2.2%)	42 (13.4%)	244 (78.0%)	69 (9.5%)	42 (60.9%)	27 (39.1%)
**Structure of abstract**								
Structured	2449 (70.7%)	183 (7.5%)	48 (2.0%)	328 (13.4%)	1890 (77.2%)	559 (77.0%)	330 (59.0%)	229 (41.0%)
Not structured	1016 (29.3%)	55 (5.4%)	20 (2.0%)	92 (9.1%)	849 (83.6%)	167 (23.0%)	92 (55.1%)	75 (44.9%)
**Types of systematic reviews**								
Intervention	2082 (60.1%)	155 (7.4%)	38 (1.8%)	251 (12.1%)	1638 (78.7%)	444 (61.2%)	252 (56.8%)	192 (43.2%)
Non-intervention	1383 (39.9%)	83 (6.0%)	30 (2.2%)	169 (12.2%)	1101 (79.6%)	282 (38.8%)	170 (60.3%)	112 (39.7%)
**Meta-analysis conducted**								
Yes	1323 (38.2%)	95 (7.2%)	16 (1.2%)	105 (7.9%)	1107 (83.7%)	216 (29.8%)	106 (49.1%)	110 (50.9%)
No	2142 (61.8%)	143 (6.7%)	52 (2.4%)	315 (14.7%)	1632 (76.2%)	510 (70.2%)	316 (62.0%)	194 (38.0%)
**Journal type**								
Dental	2393 (69.1%)	182 (7.6%)	41 (1.7%)	300 (12.5%)	1870 (78.1%)	523 (72.0%)	302 (57.7%)	221 (42.3%)
Other	1072 (30.9%)	56 (5.2%)	27 (2.5%)	120 (11.2%)	869 (81.1%)	203 (28.0%)	120 (59.1%)	83 (40.9%)
**Reporting of primary study design**								
Reported	2061 (59.5%)	183 (8.9%)	46 (2.2%)	289 (14.0%)	1543 (74.9%)	518 (71.3%)	290 (56.0%)	228 (44.0%)
Not reported	1404 (40.5%)	55 (3.9%)	22 (1.6%)	131 (9.3%)	1196 (85.2%)	208 (28.7%)	132 (63.5%)	76 (36.5%)
**Word limit of abstracts**								
<or = 250	2086 (60.2%)	137 (6.6%)	35 (1.7%)	230 (11.0%)	1684 (80.7%)	402 (55.4%)	232 (57.7%)	170 (42.3%)
> 250	1379 (39.8%)	101 (7.3%)	33 (2.4%)	190 (13.8%)	1055 (76.5%)	324 (44.6%)	190 (58.6%)	134 (41.4%)
Actual number of words in abstracts*	242 (206-278)	248 (215-298)	251 (227-289)	249 (221-294)	239 (203-274)	249 (218-294)	249 (220-294)	248 (217-295)
Number of citations (WoS Core)*	10 (2-30)	11 (3-32)	10 (3-27)	11 (2-35)	10 (2-30)	11 (3-32)	11 (2-34)	11 (3-31)
**Publisher**								
Elsevier	381 (11.0%)	36 (9.4%)	11 (2.9%)	35 (9.2%)	299 (78.5%)	82 (11.3%)	35 (42.7%)	47 (57.3%)
Springer	268 (7.7%)	11 (4.1%)	7 (2.6%)	24 (9.0%)	226 (84.3%)	42 (5.8%)	24 (57.1%)	18 (42.9%)
Wiley	785 (22.7%)	49 (6.2%)	10 (1.3%)	104 (13.2%)	622 (79.2%)	163 (22.5%)	104 (63.8%)	59 (36.2%)
Sage	72 (2.1%)	4 (5.6%)	3 (4.2%)	5 (6.9%)	60 (83.3%)	12 (1.7%)	5 (41.7%)	7 (58.3%)
MDPI	324 (9.4%)	12 (3.7%)	5 (1.5%)	34 (10.5%)	273 (84.3%)	51 (7.0%)	34 (66.7%)	17 (33.3%)
Taylor & Francis	79 (2.3%)	2 (2.5%)	0 (0.0%)	7 (8.9%)	70 (88.6%)	9 (1.2%)	7 (77.8%)	2 (22.2%)
Others	1556 (44.9%)	124 (8.0%)	32 (2.1%)	211 (13.6%)	1189 (76.4%)	367 (50.6%)	213 (58.0%)	154 (42.0%)
**Countries**								
Developing	1504 (43.4%)	132 (8.8%)	35 (2.3%)	178 (11.8%)	1159 (77.1%)	345 (47.6%)	180 (52.2%)	165 (47.8%)
Developed	1957 (56.6%)	106 (5.4%)	32 (1.6%)	242 (12.4%)	1577 (80.6%)	380 (52.4%)	242 (63.7%)	138 (36.3%)
**Continents**								
North America	368 (10.6%)	20 (5.4%)	4 (1.1%)	63 (17.1%)	281 (76.4%)	87 (12.0%)	63 (72.4%)	24 (27.6%)
South America	565 (16.3%)	89 (15.8%)	16 (2.8%)	28 (5.0%)	432 (76.5%)	133 (18.3%)	28 (21.1%)	105 (78.9%)
Europe	1404 (40.5%)	80 (5.7%)	27 (1.9%)	137 (9.8%)	1160 (82.6%)	244 (33.7%)	137 (56.1%)	107 (43.9%)
Asia	880 (25.4%)	37 (4.2%)	13 (1.5%)	148 (16.8%)	682 (77.5%)	198 (27.3%)	150 (75.8%)	48 (24.2%)
Africa	58 (1.7%)	6 (10.3%)	4 (6.9%)	8 (13.8%)	40 (69.0%)	18 (2.5%)	8 (44.4%)	10 (55.6%)
Oceania	186 (5.4%)	6 (3.2%)	3 (1.6%)	36 (19.4%)	141 (75.8%)	45 (6.2%)	36 (80.0%)	9 (20.0%)

*median and interquartile range was used

### Association between the characteristics of systematic reviews and language restrictions

#### Restricted to some languages versus no language restriction.

In the univariable analysis, meta-analysis conducted (*P* = .015) and journal type (*P* = .009) were significantly associated with systematic reviews restricted to some languages in comparison with systematic reviews with no language restriction (Table 2). In the multivariable analysis, meta-analysis conducted (*P* = .044), journal type (*P* = .040), countries (*P* = .044) and continents (*P* = .017 for South America) were significantly associated with systematic reviews restricted to some languages in comparison with systematic reviews with no language restriction (Table 3).

**Table 2:** Univariable multinominal logistic regression analysis for the association between characteristics of included systematic reviews and language restrictions (N = 3465) based on the abstracts.

	Restricted to some languages vs. No language restriction (ref.)			Restricted to English only vs. No language restriction (ref.)			No information vs. No language restriction (ref.)		
Variables	B	OR (95%CI)	P	B	OR (95%CI)	P	B	OR (95%CI)	P
**Topic of the review** ^**A**^									
Dental public health	0.54	1.71 (0.52 5.65)	0.376	0.26	1.30 (0.59 2.89)	0.516	0.36	1.43 (0.71 2.85)	0.315
Endodontics	-0.74	0.48 (0.09 2.68)	0.400	-0.14	0.87 (0.36 2.11)	0.763	-0.08	0.92 (0.44 1.94)	0.831
Oral and maxillofacial radiology Oral and maxillofacial surgery	-17.66 -0.05	- 0.95 (0.23 3.95)	- 0.946	1.05 0.24	2.86 (0.32 25.35) 1.27 (0.54 2.97)	0.346 0.582	0.49 0.66	1.64 (0.21 12.86) 1.94 (0.93 4.05)	0.638 0.078
Oral medicine	0.54	1.71 (0.32 9.11)	0.527	-0.41	0.67 (0.19 2.36)	0.530	-0.28	0.75 (0.27 2.11)	0.591
Orofacial pain	0.09	1.10 (0.29 4.21)	0.891	-0.17	0.84 (0.36 2.00)	0.697	-0.49	0.61 (0.29 1.28)	0.191
Orthodontics and dentofacial	-1.05	0.35 (0.11 1.17)	0.088	-1.17	0.31 (0.16 0.62)	<0.001*	-0.83	0.44 (0.25 0.76)	0.003*
orthopaedics									
Pediatric dentistry	-1.25	0.29 (0.03 2.65)	0.271	-0.41	0.67 (0.25 1.76)	0.413	-0.23	0.80 (0.36 1.76)	0.795
Periodontics	-0.14	0.87 (0.26 2.91)	0.820	-0.05	0.95 (0.46 1.98)	0.896	0.32	1.37 (0.74 2.55)	0.318
Prosthodontics	-0.13	0.88 (0.33 2.33)	0.796	-0.06	0.95 (0.52 1.72)	0.856	-0.05	0.95 (0.57 1.59)	0.857
Others	Ref.			Ref.			Ref.		
**Structure of abstract**									
Structured	-0.33	0.72 (0.40 1.32)	0.288	0.07	1.07 (0.73 1.57)	0.722	-0.40	0.67 (0.49 0.91)	0.012*
Not structured	Ref.			Ref.			Ref.		
**Type of systematic review**									
Intervention Non-intervention	-0.39 Ref.	0.68 (0.39 1.17)	0.165	-0.23 Ref.	0.80 (0.57 1.11)	0.174	-0.23 Ref.	0.80 (0.60 1.05)	0.108
**Meta-analysis conducted**									
Yes	-0.77	0.46 (0.25 0.86)	0.015*	-0.69	0.50 (0.36 0.71)	<0.001*	0.02	1.02 (0.78 1.34)	0.880
No	Ref.			Ref.			Ref.		
**Journal type**									
Dental	-0.76	0.47 (0.26 0.83)	0.009*	-0.26	0.77 (0.53 1.11)	0.161	-0.41	0.66 (0.49 0.90)	0.009*
Other	Ref.			Ref.			Ref.		
**Reporting of primary study design**									
Reported	-0.47	0.63 (0.35 1.13)	0.123	-0.41	0.66 (0.46 0.96)	0.027*	-0.95	0.39 (0.28 0.53)	<0.001*
Not reported	Ref.			Ref.			Ref.		
**Word limit of abstract**									
< or = 250	-0.25	0.78 (0.46 1.34)	0.372	-0.11	0.89 (0.65 1.23)	0.487	0.16	1.18 (0.90 1.54)	0.234
> 250	Ref.			Ref.			Ref.		
**Actual number of words in abstract**	0.000	1.000 (0.996 1.004)	0.864	0.000	1.000 (0.998 1.003)	0.710	-0.004	0.996 (0.994 0.998)	<0.001*
**Number of citations (WoS Core)**	-0.002	0.998 (0.992 1.005)	0.559	0.001	1.001 (0.998 1.004)	0.444	0.001	1.001 (0.998 1.003)	0.545
**Publisher** ^**A**^									
Elsevier	0.17	1.18 (0.54 2.58)	0.671	-0.56	0.57 (0.34 0.96)	0.033*	-0.14	0.87 (0.59 1.28)	0.473
Springer	0.90	2.47 (0.89 6.87)	0.084	0.25	1.28 (0.61 2.71)	0.514	0.76	2.14 (1.14 4.03)	0.018*
Wiley	-0.24	0.79 (0.36 1.73)	0.557	0.22	1.25 (0.83 1.87)	0.286	0.28	1.32 (0.94 1.87)	0.111
Sage	1.07	2.91 (0.62 13.65)	0.176	-0.31	0.74 (0.19 2.79)	0.650	0.45	1.56 (0.56 4.38)	0.394
MDPI	0.48	1.62 (0.53 4.92)	0.399	0.51	1.67 (0.83 3.34)	0.150	0.86	2.37 (1.29 4.35)	0.005*
Taylor & Francis	-17.93	-	-	0.72	2.06 (0.42 10.06)	0.373	1.30	3.65 (0.88 15.07)	0.073
Others	Ref.			Ref.			Ref.		
**Countries**									
Developing	-0.13	0.88 (0.51 1.51)	0.640	-0.53	0.59 (0.43 0.81)	0.001*	-0.53	0.59 (0.45 0.77)	<0.001*
Developed	Ref.			Ref.			Ref.		
**Continents**									
North America	-0.52	0.59 (0.19 1.89)	0.376	0.61	1.84 (1.04 3.27)	0.037*	-0.03	0.97 (0.58 1.61)	0.903
South America	-0.63	0.53 (0.27 1.06)	0.073	-1.69	0.18 (0.11 0.31)	<0.001*	-1.09	0.34 (0.24 0.46)	<0.001*
Asia	0.04	1.04 (0.48 2.24)	0.918	0.85	2.34 (1.48 3.68)	<0.001*	0.24	1.27 (0.85 1.90)	0.241
Africa	0.68	1.98 (0.52 7.53)	0.319	-0.25	0.78 (0.26 2.33)	0.654	-0.78	0.46 (0.19 1.12)	0.086
Oceania	0.39	1.48 (0.35 6.33)	0.596	1.25	3.50 (1.41 8.68)	0.007*	0.48	1.62 (0.69 3.78)	0.264
Europe	Ref.			Ref.			Ref.		

*P < 0.05; A: the variables were not included in the multivariable analysis because extremely small sample size in some categories which may influence the validity of the model; OR: odds ratio; CI: confidence interval; Ref.: reference category

**Table 3:** Multivariable multinominal logistic regression analysis for the association between characteristics of included systematic reviews and language restrictions (N = 3465) based on the abstracts.

	Restricted to some languages vs. No language restriction (ref.)			Restricted to English only vs. No language restriction (ref.)			No information vs. No language restriction (ref.)		
Variables	B	OR (95%CI)	P	B	OR (95%CI)	P	B	OR (95%CI)	P
**Structure of abstract**									
Structured	-0.18	0.84 (0.44 1.58)	0.582	0.09	1.10 (0.73 1.64)	0.655	-0.22	0.80 (0.58 1.12)	0.191
Not structured	Ref.			Ref.			Ref.		
**Meta-analysis conducted**									
Yes	-0.65	0.52 (0.28 0.98)	0.044*	-0.63	0.54 (0.38 0.76)	<0.001*	0.28	1.33 (1.00 1.76)	0.052
No	Ref.			Ref.			Ref.		
**Journal type**									
Dental	-0.63	0.54 (0.30 0.97)	0.040*	-0.06	0.95 (0.64 1.39)	0.780	-0.20	0.82 (0.59 1.13)	0.227
Other	Ref.			Ref.			Ref.		
**Reporting of primary study design**									
Reported	-0.39	0.68 (0.37 1.24)	0.209	-0.35	0.71 (0.49 1.03)	0.072	-0.85	0.43 (0.31 0.59)	<0.001*
Not reported	Ref.			Ref.			Ref.		
**Actual number of words in abstract**	0.001	1.001 (0.997 1.005)	0.759	0.000	1.000 (0.998 1.002)	0.993	-0.004	0.996 (0.994 0.998)	<0.001*
**Countries**									
Developing	2.15	8.59 (1.07 69.28)	0.044*	-0.63	0.53 (0.11 2.67)	0.443	0.21	1.23 (0.28 5.51)	0.784
Developed	Ref.			Ref.			Ref.		
**Continents**									
North America	-0.70	0.50 (0.15 1.67)	0.258	0.63	1.88 (1.06 3.34)	0.032*	-0.03	0.97 (0.58 1.63)	0.916
South America	-2.64	0.07 (0.01 0.62)	0.017*	-0.98	0.38 (0.07 2.03)	0.255	-1.42	0.24 (0.05 1.11)	0.068
Asia	-2.05	0.13 (0.02 1.13)	0.064	1.54	4.66 (0.92 23.62)	0.063	-0.07	0.94 (0.21 4.23)	0.931
Africa	-1.47	0.23 (0.02 2.69)	0.241	0.44	1.55 (0.22 10.84)	0.661	-1.02	0.36 (0.06 2.07)	0.253
Oceania	0.32	1.38 (0.32 5.94)	0.665	1.24	3.46 (1.40 8.59)	0.007*	0.43	1.53 (0.65 3.60)	0.326
Europe	Ref.			Ref.					

*P < 0.05; OR: odds ratio; CI: confidence interval; ref.: reference category

### Restricted to English only versus no language restriction

In the univariable analysis, topics of reviews (*P* < .001) for orthodontics and dentofacial orthopaedics), meta-analysis conducted (*P* < .001), reporting of primary study design (*P* = .027), publisher (*P* = .033 for Elsevier), countries (*P* = .001) and continents (*P* = .037 for North America, *P* < .001 for South America, *P* < .001 for Asia and *P* = .007 for Oceania) were significantly associated with systematic reviews restricted to English only in comparison with systematic reviews with no language restriction (Table 2).

In the multivariable analysis, meta-analysis conducted (*P* < .001) and continents (*P* = .032 for North America and *P* = .007 for Oceania) were significantly associated with systematic reviews restricted to English only in comparison with those with no language restriction (Table 3).

### No information about language versus no language restriction

In the univariable analysis, topics of reviews (*P* = .003 for orthodontics and dentofacial orthopaedics), structure of abstracts (*P* = .012), journal type (*P* = .009), reporting of primary study design (*P* < .001), actual number of words in abstracts (*P* < .001), publisher (*P* = .018 for Springer and *P* = .005 for MDPI), countries (*P* < .001) and continents (*P* < .001 for South America) were significantly associated with systematic reviews with no information in comparison with those with no language restriction (Table 2).

In the multivariable analysis, reporting of primary study design (*P* < .001) and actual number of words in abstracts (*P* < .001) were significantly associated with systematic reviews with no information of the language used in examined articles in comparison with systematic reviews with no language restriction (Table 3).

An explanation of these associations is provided in the supplementary file.

### Association between the characteristics of systematic reviews and the number of included languages

In the univariable analysis, topics of the review (*P* = .019), meta-analysis (*P* = .001), publisher (*P* = .029), countries (*P* = .002) and continents (*P* < .001) were significantly associated with the number of included languages (Table 4).

**Table 4:** Univariable and multivariable binary logistic regression analysis for the association between characteristics of included systematic reviews and number of included languages (N = 726) based on the abstracts.

	Univariable			Multivariable		
Variables	B	OR (95%CI) (only 1 language as the reference)	P value	B	OR (95%CI) (only 1 language as the reference)	P value
**Topic of the review**			0.019*			0.002*
Dental public health	-0.09	0.91 (0.45 1.83)	0.793	0.16	1.17 (0.52 2.67)	0.703
Endodontics	-0.01	0.99 (0.43 2.26)	0.981	0.34	1.41 (0.55 3.62)	0.477
Oral and maxillofacial radiology	-1.35	0.26 (0.03 2.27)	0.223	-2.26	0.11 (0.01 1.13)	0.063
Oral and maxillofacial surgery	-0.25	0.78 (0.36 1.68)	0.523	-0.35	0.71 (0.29 1.71)	0.444
Oral medicine	0.58	1.78 (0.58 5.47)	0.316	0.86	2.37 (0.63 8.88)	0.199
Orofacial pain	0.10	1.10 (0.50 2.42)	0.809	0.00	1.00 (0.40 2.48)	0.997
Orthodontics and dentofacial orthopaedics	0.98	2.67 (1.40 5.13)	0.003*	1.21	3.35 (1.58 7.08)	0.002*
Pediatric dentistry	0.20	1.22 (0.48 3.09)	0.671	0.40	1.49 (0.51 4.33)	0.462
Periodontics	0.01	1.01 (0.52 1.98)	0.966	0.09	1.10 (0.50 2.39)	0.814
Prosthodontics	0.01	1.01 (0.58 1.74)	0.980	0.03	1.03 (0.54 1.97)	0.933
Others	Ref.			Ref.		
**Structure of abstract**						
Structured	-0.16	0.85 (0.60 1.21)	0.365			
Not structured	Ref.					
**Type of systematic reviews**						
Intervention	0.15	1.16 (0.85 1.57)	0.348			
Non-intervention	Ref.					
**Meta-analysis conducted**						
Yes	0.53	1.69 (1.23 2.33)	0.001*	0.55	1.73 (1.19 2.53)	0.004*
No	Ref.			Ref.		
**Journal type**						
Dental	0.06	1.06 (0.76 1.47)	0.737			
Other	Ref.					
**Reporting of primary study design**						
Reported	0.31	1.37 (0.98 1.90)	0.065			
Not reported	Ref.					
**Word limit of abstracts**						
< or = 250	0.04	1.04 (0.77 1.40)	0.801			
> 250	Ref.					
**Actual number of words in abstracts**	-0.001	0.999 (0.997 1.002)	0.651			
**Number of citations (WoS Core)**	-0.002	0.998 (0.995 1.001)	0.243			
**Publisher**			0.029*			0.010*
Elsevier	0.62	1.86 (1.14 3.01)	0.012*	0.62	1.86 (1.07 3.24)	0.028*
Springer	0.04	1.04 (0.54 1.98)	0.911	-0.22	0.81 (0.37 1.75)	0.583
Wiley	-0.24	0.79 (0.54 1.15)	0.212	-0.43	0.65 (0.41 1.02)	0.062
Sage	0.66	1.94 (0.60 6.22)	0.267	1.15	3.15 (0.89 11.15)	0.076
MDPI	-0.37	0.69 (0.37 1.28)	0.242	-0.50	0.61 (0.30 1.22)	0.160
Taylor & Francis	-0.93	0.40 (0.08 1.93)	0.251	-0.35	0.71 (0.12 4.22)	0.701
Others	Ref.			Ref.		
**Countries**						
Developing	0.48	1.61 (1.19 2.16)	0.002*	2.04	7.71 (0.80 74.66)	0.078
Developed	Ref.			Ref.		
**Continents**			< 0.001*			<0.001*
North America	-0.72	0.49 (0.29 0.83)	0.008*	-0.83	0.44 (0.24 0.79)	0.006*
South America	1.57	4.80 (2.95 7.81)	<0.001*	-0.29	0.75 (0.07 7.71)	0.809
Asia	-0.89	0.41 (0.27 0.62)	<0.001*	-3.04	0.05 (0.01 0.48)	0.010*
Africa	0.47	1.60 (0.61 4.19)	0.339	-1.67	0.19 (0.02 2.27)	0.189
Oceania	-1.14	0.32 (0.15 0.69)	0.004*	-1.08	0.34 (0.15 0.77)	0.010*
Europe	Ref.					

*P < 0.05; OR: odds ratio; CI: confidence inter; ref.: reference category

In the multivariable analysis, topics of the reviews (*P* = .002), meta-analysis (*P* = .004), publisher (*P* = .010) and continents (*P* < .001) remained statistically significant (Table 4).

An explanation of these associations is provided in the supplementary file.

### Differences in reporting language restrictions between the abstract and the full text

Based on Table 5, the proportions of the full texts reporting no language restriction, restriction to some languages, restriction to English only, and no information about language restriction are 27%, 13%, 53%, and 7%, respectively.

**Table 5:** Language restrictions reported in the abstracts and full texts.

	Abstracts					
**Full texts**		No language restriction	Restricted to some languages	Restricted to English only	No information	Total
	No language restriction	**18**	0	0	67	85
	Restricted to some languages	1	**9**	0	30	40
	Restricted to English only	3	0	**36**	130	169
	No information	1	0	1	**23**	25
	Total	23	9	37	250	319

Among the 23 studies reporting no language restrictions in the abstracts, 18 (78%) of them also reported the same in the full texts. Among the 9 studies reporting restriction to some languages in the abstracts, all (100%) also reported the same in the full texts. Among the 37 studies reporting restriction to English only in the abstracts, 36 (97%) also reported the same in the full texts. This is contrasted with the finding that among the 250 studies reporting no information about language restriction in the abstracts, only 23 studies (9%) reported the same in the full texts, while 67 (27%), 30 (12%), and 130 (52%) studies reported no language restriction, restriction to some languages, and restriction to English only, respectively (Table 5).

## Discussion

### Main findings

This meta-research assessing the reporting of languages in abstracts of systematic reviews in the dental field found that the great majority (79%) of the abstracts did not report any information about language restriction. Meta-analysis conducted, journal type, reporting of primary study design, actual number of words in abstract, country and continent of first authors´ affiliation were significantly associated with the reporting of language restrictions in the abstracts of systematic reviews. In addition, the topic of the review, meta-analysis conducted, publisher and continents were significantly associated with the number of languages reported.

### Interpretation and relevance of the findings

To the best of current knowledge, this is the first study to comprehensively assess the reporting of language in the abstracts of systematic reviews in any biomedical field. The findings emphasize the importance of more detailed and consistent reporting of the language in the abstracts of systematic reviews.

More detailed information would allow a more accurate interpretation of the findings by the readers, for example, when the effect size of the meta-analytic estimates is small and there is no information in the abstract about whether there was any limitation set in the search strategies regarding languages of the examined articles. In such cases, one cannot be sure whether potential language bias could in fact influence the size of these estimates.

Some evidence suggests that not including primary studies in languages other than English will not alter the conclusions of a systematic review [9,10]. However, the scope of the discussion of potential language bias should be broader. Some authors suggest that primary studies published in some languages can provide more positive results [11]. This assumption is corroborated by evidence of language and indexing bias in Chinese-sponsored clinical trials that suggest positive trials are more likely to be published in English than negative trials [12].

In the cited study [12], Chinese-sponsored trials with positive results and registered in the Chinese Clinical Trial Registry were 3.92 (95% CI, 2.20–7.00) times more likely to be published in English than Chinese when compared with negative Chinese-sponsored trials. Similarly, Chinese-sponsored trials with positive results and registered in English-language registries were 3.22 (95% CI, 1.34–7.78) times more likely to be published in English than Chinese when compared with negative Chinese-sponsored trials.

Furthermore, various authors reported issues other than language bias when limiting the search in systematic reviews. For example, a language limitation in systematic reviews of pharmacogenomics and pharmacogenetics may hinder the generalisability of findings to non-native English-language populations [13].

In fact, constructing a comprehensive search and including studies without language restriction minimizes the chance of publication bias [14]. A comprehensive search means a detailed search strategy, applied in multiple databases and grey literature without language and publication date restrictions. Such a strategy contributes to a more realistic effect estimate of a specific intervention. Language restrictions, on the other hand, may yield to an effect overestimation.

In the regression analyses, several variables were found to be significantly associated with language restrictions in the abstracts. For example, systematic reviews with meta-analyses may be more likely to include more comprehensive literature in all available languages to generate more precise meta-analytic estimates than systematic reviews without meta-analyses. This could be why systematic reviews with a meta-analysis were likely to have fewer language restrictions than those without a meta-analysis. Furthermore, it was observed that systematic reviews originating from North America or Oceania were more likely to impose language restrictions, whereas those from South America were less likely to do so.

This may be because English is the main official language on those continents (e.g., Canada and USA in North America) and Australia and New Zealand in Oceania.

Therefore, systematic reviews from those two continents are prone to be restricted to English alone. In contrast, countries in South America have a greater variety of official languages, such as Spanish, Portuguese, French, English and Dutch. In addition, dental journals are more likely to have language restrictions than non-dental journals. This may indicate that systematic reviews in dentistry need to include more comprehensive literature to improve the external validity of the results in the future. Most of the other significant associations remain to be explained.

A random sample of 9.2% of the full texts was reviewed and it appears that there is a poor consistency in reporting no information about language restriction between abstracts and full texts. When no information about language was reported in the abstract, 91% of the full texts of these abstracts reported information about language restriction. On the contrary there is good consistency in reporting no language restrictions, restriction to some languages and restriction to English only between abstracts and full texts.

### Strengths and limitations

The study has several strengths. It is the first study in dentistry to assess language restrictions in the abstracts of systematic reviews. The sample size was substantial and may be representative of the dental field. Furthermore, this large sample of abstracts rendered a robust statistical assessment with several variables.

This study has certain limitations. Some information regarding language restrictions that was not reported in the abstracts may have been included in the full texts of the articles. An analysis of the full texts from a 9.2% sample of all included studies supports this assumption. However, restricting our analysis to abstracts can also be considered a strength in terms of relevance, as many readers rely solely on abstracts when assessing scientific articles. Therefore, comprehensive reporting within abstracts is crucial.

Additionally, the sample size used in the multinomial regression analysis may be insufficient. According to the rule of events per variable (EPV > 10), at least 300 samples per category of the dependent variable (language restriction) are required, given that 12 independent variables with 30 categories were included. However, some categories within the dependent variable contained fewer than 300 samples, potentially reducing the statistical power of the analysis.

### Further developments

Reporting checklists, such as PRISMA for Abstracts, could be updated to include more detailed information on guiding researchers to report on the language of examined articles in the abstracts of systematic reviews. For example, by guiding authors to declare whether language restrictions were imposed in the search strategy phase or whether language restriction was applied as an eligibility criterion [4].

Furthermore, a similar assessment should be conducted in other medical disciplines to understand whether the problem of this type of underreporting is widespread in the biomedical field.

## Conclusion

This meta-research study demonstrates that abstracts of systematic reviews in dentistry frequently underreport language restrictions applied to the primary studies examined. Several characteristics of systematic reviews are significantly associated with these restrictions, underscoring inconsistencies in reporting practices. This study emphasizes the necessity of reevaluating reporting practices to enhance transparency and standardization.

## Supporting information

S1 Data (XLSX)

S1 File Supplementary file. (DOCX)
