# Supplementary material for: The assessment of language restrictions in abstracts of systematic reviews in dentistry: A meta-research study
Source: PLoS One. 2025 May 20;20(5):e0323176. doi: 10.1371/journal.pone.0323176 (PMC12092015; doi:10.1371/journal.pone.0323176)
Supplement: S1 File — (DOCX) [file pone.0323176.s002.docx]

**Supplementary file**

**Content**

I List of abbrevations

II Search strategy

III Rationale for the assessment

IV Explanation of the associations

V List of excluded articles (n= 448) with reasons for exclusion

**I List of abbreviations**

MA=Meta-Analysis; SR= Systematic Review; SRs= Systematic Reviews

**II Search strategy**

Search in Web of Science (24 February 2023)

3 #1 AND #2

3,922

2 TI=(systematic)

306,005

1 AB=(dentistry OR "dental occlusion" OR "oral diagnosis" OR endodontics OR orthodontics OR "oral pathology" OR periodontics OR prosthodontics OR "oral surgery" OR "oral medicine" OR "dental restoration" OR "dental implants" OR "dental implantation" OR “dental care” OR peri-implantitis OR osseointegration OR pulpectomy OR pulpotomy OR “root canal therapy” OR “guided tissue regeneration” OR “root planing” OR “periodontal medicine” OR “orthodontic appliances” OR “dental prosthesis” OR “prosthetic dentistry” OR “mouth diseases” OR “oral surgical procedure” OR “dental caries” OR “tooth diseases” OR “Stomatognathic Diseases” OR “dental diseases” OR “oral cancer” OR “mouth neoplasm” OR “periodontal disease” OR “periapical disease” OR “oral health” OR “guided bone regeneration” OR “sinus floor augmentation” OR “oral hygiene” OR “dental prophylaxis” OR “dental scaling” OR “dental cavity” OR “dental health education” OR “dental bonding” OR “dental equipment” OR “periodontal debridement” OR “orthognathic surgery” OR “composite resins” OR “dental materials” OR “dental porcelain” OR “dental sealants” OR “tooth sealant” OR dentifrices OR toothpastes OR “dental polishes” OR “dental adhesive” OR “dental cement” OR “orthodontic adhesives” OR “cariogenic agents” OR “cariostatic agents” OR “topical fluorides” OR “dental impression materials” OR “dental inlay” OR “dental onlay” OR dentures OR “dental veneers” OR “periodontal prosthesis” OR “dental crowns” OR “tooth extraction” OR “crown lengthening” OR “orthodontic extrusion” OR “occlusal adjustment” OR “occlusal splints” OR “temporomandibular joint syndrome” OR “TMJ syndrome” OR “tooth mobility” OR “masticatory forces” OR “orthodontic retainers”)

**III Rationale for assessment**

Data extraction was primarily conducted by reading and assessing the titles and the abstracts (February and October 2023). This process was repeated a minimum of three times to ensure accuracy.

For language restrictions (a), information was extracted regarding language restrictions for the inclusion of articles in SRs and what languages were stated (only English or other languages).

For SRs with or without MA (c), it was extracted if the abstract or title implied a performed MA. If there was nothing stated about MA or a reason why an MA couldn’t be performed, it was extracted as “no MA performed.”

For SR type (d), the abstracts were grouped into different types of SRs. As a result, the abstracts were categorized into two groups: “Intervention” (includes therapies and treatment) and “non-intervention” (includes prevalence, diagnosis, other).

For type of abstract (e), it was extracted if the abstract summarized the main sections of the paper in an unstructured or structured format (divided into different sections, each with a heading).

 For type of primary study included in the SR (f), it was extracted if the type of primary study included in the SR was reported (e.g., “Case studies,” “randomized controlled trials”). It was extracted as "not reported" if there was no or unclear information about included study designs (e.g., “studies,” “literature”).

For dental field (h), the abstracts were grouped into ten different categories based on content.

The data of source title (k) enabled the extraction of the journal type (b) by: 1. categorizing journals into dental/oral field or other, and 2. extracting the word limits for SR abstracts from the official guidelines for authors (from publishers’ websites or journal websites).

With the related data of authors (i) and affiliations (m), the country of the first author (g) was extracted by researching the countries of the affiliated organizations. For 111 data series, affiliations (m) data were missing, so the affiliated country of the authors was researched online (e.g., PubMed, ResearchGate, National Library).

**IV Explanation of the associations**

Regression analyses demonstrated that when compared with no language restrictions, SRs with meta-analysis conducted, published in dental journals, and performed in South America have significantly lower odds to restrict to some languages than SRs with no meta-analysis conducted, published in other journals than dentistry, and performed in Europe. SRs performed in developing countries have significantly higher odds to restrict to some languages than SRs performed in developed countries, when compared with no language restrictions. Systematic reviews with meta-analysis conducted have significantly lower odds to restrict to English only than SRs with no meta-analysis conducted. SRs performed in North America and Oceania have significantly higher odds to restrict to English only than SRs from Europe, when compared with no language restrictions. When compared with no language restrictions, SRs that reported the design of primary studies and had larger number of words in abstracts have significantly lower odds to report no information in language than SRs without reporting the design of the reviewed studies and smaller number of words in abstracts (Table 3).

Systematic reviews in the field of and dentofacial orthopaedics, with meta-analysis conducted, and published in Elsevier have significantly higher odds to include >1 type of language in the SRs, than the SRs in “other” fields, without meta-analysis conducted and published in “other” publishers. In addition, SRs from North America, Asia, and Oceania have significantly lower odds to include >1 type of language than SRs from Europe (Table 4).

**V List of excluded articles (n=448) with reasons for exclusion**

1. Abdelbasset, WK; Jasim, SA; Bokov, DO; Shalaby, MN; Opulencia, MJC; Thangavelu, L; Alkadir, OKA; Ansari, MJ; Kzar, HH; Al-Gazally, ME. Polysaccharides, as biological macromolecule-based platforms in skeletal muscle tissue engineering: a systematic review. INTERNATIONAL JOURNAL OF POLYMERIC MATERIALS AND POLYMERIC BIOMATERIALS. 2023. http://dx.doi.org/10.1080/00914037.2022.2090940.
   1. Reason: Not primary an oral health topic.
2. Abed, H; Alhabshi, M; Alkhayal, Z; Burke, M; Nizarali, N. Oral and dental management of people with myelodysplastic syndromes and acute myeloid leukemia: A systematic search and evidence-based clinical guidance. SPECIAL CARE IN DENTISTRY 2019. http://dx.doi.org/10.1111/scd.12384.
   1. Reason: Not a SR.
3. Ahmad, P; Dummer, PMH; Chaudhry, A; Rashid, U; Saif, S; Asif, JA. A bibliometric study of the top 100 most-cited randomized controlled trials, systematic reviews and meta-analyses published in endodontic journals. INTERNATIONAL ENDODONTIC JOURNAL 2019. http://dx.doi.org/10.1111/iej.13131.
   1. Reason: Not a SR.
4. Akl, EA; Gaddam, S; Gunukula, SK; Honeine, R; Abou Jaoude, P; Irani, J. The effects of waterpipe tobacco smoking on health outcomes: a systematic review. INTERNATIONAL JOURNAL OF EPIDEMIOLOGY 2010. http://dx.doi.org/10.1093/ije/dyq002.
   1. Reason: Not primary an oral health topic.
5. Al Moaleem, MM; Al-layl, OA; Alhomood, MA; Ageeli, WA; Qsadi, AA; Mohammed, TIM; Baraqt, ES; Zubayni, BMA; Alalmaie, NI; Huraysi, AHM. Computer Engineering Complete Dentures Workflow: Systematic Techniques Review. JOURNAL OF PHARMACEUTICAL RESEARCH INTERNATIONAL 2021 http://dx.doi.org/10.9734/JPRI/2021/v33i53A33653.
   1. Reason: Not a SR.
6. Alanko, OME; Svedstrom-Oristo, AL; Tuomisto, MT. Patients' perceptions of orthognathic treatment, well-being, and psychological or psychiatric status: a systematic review. ACTA ODONTOLOGICA SCANDINAVICA 2010. http://dx.doi.org/10.3109/00016357.2010.494618.
   1. Not primary an oral health topic.
7. Alarcon, MA; Diaz, KT; Aranda, L; Cafferata, EA; Faggion, CM; Monje, A. Use of Biologic Agents to Promote Bone Formation in Implant Dentistry: A Critical Assessment of Systematic Reviews. INTERNATIONAL JOURNAL OF ORAL & MAXILLOFACIAL IMPLANTS 2017. http://dx.doi.org/10.11607/jomi.5101.
   1. Reason: Not a SR.
8. Alkadhimi, A; Reeves, S; DiBiase, AT. How to appraise the literature: basic principles for the busy clinician-part 2: systematic reviews and meta-analyses. BRITISH DENTAL JOURNAL 2022. http://dx.doi.org/10.1038/s41415-022-4151-8.
   1. Reason: Not primary an oral health topic.
9. Alkhutari, AS; Al-Moraissi, EA; Galvao, EL; Christidis, N; Falci, SGM. Top 100 cited systematic reviews and meta-analyses in the major journals of oral and maxillofacial surgery: a bibliometric analysis. ORAL AND MAXILLOFACIAL SURGERY-HEIDELBERG 2022. http://dx.doi.org/10.1007/s10006-021-00981-9.
   1. Reason: Not a SR.
10. Alleman, DS; Magne, P. A systematic approach to deep caries removal end points: The peripheral seal concept in adhesive dentistry. QUINTESSENCE INTERNATIONAL 2012.
    1. Reason: Not a SR.
11. Alnamankany, A; Ashley, P. Assessment of the quality of reporting of randomized clinical trials in paediatric dentistry: A comparative systematic review. JOURNAL OF TAIBAH UNIVERSITY MEDICAL SCIENCES 2020. http://dx.doi.org/10.1016/j.jtumed.2019.12.006.
    1. Reason: Not primary an oral health topic.
12. Alves, CPD; Vetromilla, BM; Moreno, LB; Helal, L; Sarkis-Onofre, R; Pereira-Cenci, T. Systematic reviews on the success of dental implants present low spin of information but may be better reported and interpreted: An overview of systematic reviews with meta-analysis. CLINICAL IMPLANT DENTISTRY AND RELATED RESEARCH 2022. http://dx.doi.org/10.1111/cid.13067.
    1. Reason: Not a SR.
13. Anaya, MM; Franco, JVA; Ballesteros, M; Sola, I; Cuchi, GU; Cosp, XB. Evidence mapping and quality assessment of systematic reviews on therapeutic interventions for oral cancer. CANCER MANAGEMENT AND RESEARCH 2019. http://dx.doi.org/10.2147/CMAR.S186700.
    1. Reason: Not a SR.
14. Anderson, LM; St Charles, J; Fullilove, MT; Scrimshaw, SC; Fielding, JE; Normand, J. Providing affordable family housing and reducing residential segregation by income - A systematic review. AMERICAN JOURNAL OF PREVENTIVE MEDICINE 2003. http://dx.doi.org/10.1016/S0749-3797(02)00656-6.
    1. Reason: Not primary an oral health topic.
15. Andrade, L; Lee, KM; Sylvetsky, AC; Kirkpatrick, SI. Low-calorie sweeteners and human health: a rapid review of systematic reviews. NUTRITION REVIEWS 2021. http://dx.doi.org/10.1093/nutrit/nuaa123.
    1. Reason: Not primary an oral health topic. Review of SRs.
16. Andresen, T; Bahr, C; Ciranna-Raab, C. Efficacy of osteopathy and other manual treatment approaches for malocclusion - A systematic review of evidence. INTERNATIONAL JOURNAL OF OSTEOPATHIC MEDICINE 2013.  http://dx.doi.org/10.1016/j.ijosm.2012.07.006.
    1. Reason: Primary studies and review(s) were included in the assessment.
17. Asthana, S; Vohra, P; Labani, S. Association of smokeless tobacco with oral cancer: A review of systematic reviews. TOBACCO PREVENTION & CESSATION 2019. http://dx.doi.org/10.18332/tpc/112596.
    1. Reason: Not a SR.
18. Astvaldsdottir, A; Bostrom, AM; Davidson, T; Gabre, P; Gahnberg, L; Englund, GS; Skott, P; Stahlnacke, K; Tranaeus, S; Wilhelmsson, H; Wardh, I; Ostlund, P; Nilsson, M. Oral health and dental care of older persons-A systematic map of systematic reviews. GERODONTOLOGY 2018. http://dx.doi.org/10.1111/ger.12368.
    1. Reason: Not a SR.
19. Attin, T; Hannig, C; Wiegand, A; Attin, R. Effect of bleaching on restorative materials and restorations - a systematic review. DENTAL MATERIALS 2004. http://dx.doi.org/10.1016/j.dental.2004.04.002.
    1. Reason: Unclear whether only primary studies were included the in assessment.
20. Azarpazhooh, A; Limeback, H. The application of ozone in dentistry: A systematic review of literature. JOURNAL OF DENTISTRY 2008. http://dx.doi.org/10.1016/j.jdent.2007.11.008.
    1. Reason: Primary studies and review(s) were included in the assessment.
21. Aziz, T; Compton, S; Nassar, U; Matthews, D; Ansari, K; Flores-Mir, C. Methodological quality and descriptive characteristics of prosthodontic-related systematic reviews. JOURNAL OF ORAL REHABILITATION 2013. http://dx.doi.org/10.1111/joor.12028.
    1. Reason: Not a SR.
22. Azzopardi, PS; Kennedy, EC; Patton, GC; Power, R; Roseby, RD; Sawyer, SM; Brown, AD. The quality of health research for young Indigenous Australians: systematic review. MEDICAL JOURNAL OF AUSTRALIA 2013. http://dx.doi.org/10.5694/mja12.11141.
    1. Reason: Not primary an oral health topic.
23. Bachelet, VC; Carrasco, VA; Bravo-Cordova, F; Diaz, RA; Lizana, FJ; Meza-Ducaud, N; Pardo-Hernandez, H; Uribe, FA; Vergara, AF; Villanueva, J; Navarrete, MS. Quality of reporting for randomised clinical trials published in Latin American and Spanish journals: A protocol for a systematic survey of three clinical specialities. BMJ OPEN 2020. http://dx.doi.org/10.1136/bmjopen-2019-036148.
    1. Reason: Not primary an oral health topic. Not a SR.
24. Bachelet, VC; Navarrete, MS; Barrera-Riquelme, C; Carrasco, VA; Dallaserra, M; Diaz, RA; Ibarra, AA; Lizana, FJ; Meza-Ducaud, N; Saavedra, MG; Tapia-Davegno, C; Vergara, AF; Villanueva, J. A multiyear systematic survey of the quality of reporting for randomised trials in dentistry, neurology and geriatrics published in journals of Spain and Latin America. BMC MEDICAL RESEARCH METHODOLOGY 2021. http://dx.doi.org/10.1186/s12874-021-01337-3.
    1. Reason: Not primary an oral health topic. Not a SR.
25. Bader, J; Ismail, A. Survey of systematic reviews in dentistry. JOURNAL OF THE AMERICAN DENTAL ASSOCIATION 2004 http://dx.doi.org/10.14219/jada.archive.2004.0212.
    1. Reason: Not a SR.
26. Baines, RL; de Bere, SR. Optimizing patient and public involvement (PPI): Identifying its essential and desirable principles using a systematic review and modified Delphi methodology. HEALTH EXPECTATIONS 2018 http://dx.doi.org/10.1111/hex.12618.
    1. Reason: Not primary an oral health topic.
27. Bakdach, WMM; Hadad, R. Effectiveness of different adjunctive interventions in the management of orthodontically induced white spot lesions: A systematic review of systematic reviews and meta-analyses. DENTAL AND MEDICAL PROBLEMS 2020. http://dx.doi.org/10.17219/dmp/118330.
    1. Reason:
28. Banfai-Csonka, H; Betlehem, J; Deutsch, K; Derzsi-Horvath, M; Banfai, B; Financz, J; Podraczky, J; Csima, M. Health Literacy in Early Childhood: A Systematic Review of Empirical Studies. CHILDREN-BASEL 2022. http://dx.doi.org/10.3390/children9081131.
    1. Reason: Not primary an oral health topic.
29. Bao, JH; Huang, XY; Wang, L; He, YD; Rasubala, L; Ren, YF. Clinical practice guidelines for oral health care during pregnancy: a systematic evaluation and summary recommendations for general dental practitioners. QUINTESSENCE INTERNATIONAL 2022. http://dx.doi.org/10.3290/j.qi.b2644863.
    1. Reason: Not a SR.
30. Barbosa, E; Pires, PGS; Hauptli, L; Moraes, P. Strategies to improve the home care of periodontal disease in dogs: A systematic review. RESEARCH IN VETERINARY SCIENCE 2023. http://dx.doi.org/10.1016/j.rvsc.2022.10.025.
    1. Reason: Not primary a human oral health topic.
31. Barrere, S; Reina, N; Peters, OA; Rapp, L; Vergnes, JN; Maret, D. Dental assessment prior to orthopedic surgery: A systematic review. ORTHOPAEDICS & TRAUMATOLOGY-SURGERY & RESEARCH 2019. http://dx.doi.org/10.1016/j.otsr.2019.02.024.
    1. Reason: Not primary an oral health topic.
32. Barry, MA; Aldawsari, SA; Alrashidi, SM; Alshehri, RMA; Alhussain, BS; Ansari, SH. Impact of COVID-19 on the Utilization of Dental Services: A Systematic Review. JOURNAL OF PHARMACEUTICAL RESEARCH INTERNATIONAL 2021. http://dx.doi.org/10.9734/JPRI/2021/v33i51A33487.
    1. Reason: Not primary an oral health topic.
33. Bassani, R; Pereira, GKR; Page, MJ; Tricco, AC; Moher, D; Sarkis-Onofre, R. Systematic reviews in dentistry: Current status, epidemiological and reporting characteristics. JOURNAL OF DENTISTRY 2019. http://dx.doi.org/10.1016/j.jdent.2019.01.014.
    1. Reason: Not a SR.
34. Batista, KBDL; Thiruvenkatachari, B; O'Brien, K. Intention-to-treat analysis: Are we managing dropouts and missing data properly in research on orthodontic treatment? A systematic review. AMERICAN JOURNAL OF ORTHODONTICS AND DENTOFACIAL ORTHOPEDICS 2019. http://dx.doi.org/10.1016/j.ajodo.2018.08.013.
    1. Reason: Not primary an oral health topic.
35. Beecher, T; James, P; Browne, J; Di Blasi, Z; Harding, M; Whelton, H. Dental patient reported outcome and oral health-related quality of life measures: protocol for a systematic evidence map of reviews. BDJ OPEN 2021. http://dx.doi.org/10.1038/s41405-021-00065-6.
    1. Reason: Not a SR.
36. Begum, SN; Ray, AS; Rahaman, CH. A comprehensive and systematic review on potential anticancer activities of eugenol: From pre-clinical evidence to molecular mechanisms of action. PHYTOMEDICINE 2022. http://dx.doi.org/10.1016/j.phymed.2022.154456.
    1. Reason: Not primary an oral health topic.
37. Beks, H; Walsh, S; Alston, L; Jones, M; Smith, T; Maybery, D; Sutton, K; Versace, VL. Approaches Used to Describe, Measure, and Analyze Place of Practice in Dentistry, Medical, Nursing, and Allied Health Rural Graduate Workforce Research in Australia: A Systematic Scoping Review. INTERNATIONAL JOURNAL OF ENVIRONMENTAL RESEARCH AND PUBLIC HEALTH 2022. http://dx.doi.org/10.3390/ijerph19031438.
    1. Reason: Not primary an oral health topic.
38. Bendersky, J; Uribe, M; Bravo, M; Vargas, JP; Flores, E; Aguero, I; Villanueva, J; Urrutia, G; Bon, X. Systematic mapping review of orthognathic surgery. JOURNAL OF STOMATOLOGY ORAL AND MAXILLOFACIAL SURGERY 2022. http://dx.doi.org/10.1016/j.jormas.2022.05.011.
    1. Reason: Primary studies and review(s) were included in the assessment.
39. Bendersky, J; Uribe, M; Bravo, M; Vargas, JP; Villanueva, J; Urrutia, G; Bonfill, X. Systematic Mapping Review of Orthognathic Surgery (Protocol). CRANIOMAXILLOFACIAL TRAUMA & RECONSTRUCTION. http://dx.doi.org/10.1177/19433875221078385.
    1. Reason: Primary studies and review(s) were included in the assessment.
40. Bergamini, M; Simeone, G; Verga, MC; Doria, M; Cuomo, B; D'Antonio, G; Dello Iacono, I; Di Mauro, G; Leonardi, L; Miniello, VL; Palma, F; Scotese, I; Tezza, G; Caroli, M; Vania, A. Complementary Feeding Caregivers' Practices and Growth, Risk of Overweight/Obesity, and Other Non-Communicable Diseases: A Systematic Review and Meta-Analysis. NUTRIENTS 2022. http://dx.doi.org/10.3390/nu14132646.
    1. Reason: Not primary an oral health topic.
41. Berkman, ND; Sheridan, SL; Donahue, KE; Halpern, DJ; Crotty, K. Low Health Literacy and Health Outcomes: An Updated Systematic Review. ANNALS OF INTERNAL MEDICINE 2011. http://dx.doi.org/10.7326/0003-4819-155-2-201107190-00005.
    1. Reason: Not primary an oral health topic.
42. Bernabe, E; Marcenes, W; Hernandez, CR; Bailey, J; Abreu, LG; Alipour, V; Amini, S; Arabloo, J; Arefi, Z; Arora, A; Ayanore, MA; Barnighausen, TW; Chan, TH; Bijani, A; Cho, DY; Chu, DT; Crowe, CS; Demoz, GT; Demsie, DG; Forooshani, ZSD; Du, M; El Tantawi, M; Fischer, F; Folayan, MO; Futran, ND; Geramo, YCD; Haj-Mirzaian, A; Hariyani, N; Hasanzadeh, A; Hassanipour, S; Hay, SI; Hole, MK; Hostiuc, S; Ilic, MD; James, SL; Kalhor, R; Kemmer, L; Keramati, M; Khader, YS; Kisa, S; Kisa, A; Koyanagi, A; Lalloo, R; Le Nguyen, Q; London, SD; Manohar, ND; Massenburg, BB; Mathur, MR; Meles, HG; Mestrovic, T; Mohammadian-Hafshejani, A; Mohammadpourhodki, R; Mokdad, AH; Morrison, SD; Nazari, J; Nguyen, TH; Nguyen, CT; Nixon, MR; Olagunju, TO; Pakshir, K; Pathak, M; Rabiee, N; Rafiei, A; Ramezanzadeh, K; Rios-Blancas, MJ; Roro, EM; Sabour, S; Samy, AM; Sawhney, M; Schwendicke, F; Shaahmadi, F; Shaikh, MA; Stein, C; Tovani-Palone, MR; Tran, BX; Unnikrishnan, B; Vu, GT; Vukovic, A; Warouw, TSS; Zaidi, Z; Zhang, ZJ; Kassebaum, NJ. Global, Regional, and National Levels and Trends in Burden of Oral Conditions from 1990 to 2017: A Systematic Analysis for the Global Burden of Disease 2017 Study. JOURNAL OF DENTAL RESEARCH 2020. http://dx.doi.org/10.1177/0022034520908533.
    1. Reason: Not a SR.
43. Bezdjian, A; Smith, RA; Thomeer, HGXM; Willie, BM; Daniel, SJ. A Systematic Review on Factors Associated With Percutaneous Bone Anchored Hearing Implants Loss. OTOLOGY & NEUROTOLOGY 2018. http://dx.doi.org/10.1097/MAO.0000000000002041.
    1. Reason: Not primary an oral health topic.
44. Bhagat, S; Agarwal, M; Roy, V. Serratiopeptidase: A systematic review of the existing evidence. INTERNATIONAL JOURNAL OF SURGERY 2013. http://dx.doi.org/10.1016/j.ijsu.2013.01.010.
    1. Reason: Not primary an oral health topic.
45. Bidra, AS. Technique for systematic bone reduction for fixed implant-supported prosthesis in the edentulous maxilla. JOURNAL OF PROSTHETIC DENTISTRY 2015. http://dx.doi.org/10.1016/j.prosdent.2015.01.011.
    1. Reason: Not a SR.
46. Bijle, MNA; Yiu, CKY; Ekambaram, M. CALCIUM-BASED CARIES PREVENTIVE AGENTS: A META-EVALUATION OF SYSTEMATIC REVIEWS AND META-ANALYSIS. JOURNAL OF EVIDENCE-BASED DENTAL PRACTICE 2018. http://dx.doi.org/10.1016/j.jebdp.2017.09.003.
    1. Reason: Not a SR.
47. Bodison, SC; Parham, LD. Specific Sensory Techniques and Sensory Environmental Modifications for Children and Youth With Sensory Integration Difficulties: A Systematic Review. AMERICAN JOURNAL OF OCCUPATIONAL THERAPY 2018. http://dx.doi.org/10.5014/ajot.2018.029413.
    1. Reason: Not primary an oral health topic.
48. Bolognesi, C; Bruzzone, M; Ceppi, M; Marcon, F. Micronuclei and upper body cancers (head, neck, breast cancers) a systematic review and meta-analysis. MUTATION RESEARCH-REVIEWS IN MUTATION RESEARCH 2021. http://dx.doi.org/10.1016/j.mrrev.2020.108358.
    1. Reason: Not primary an oral health topic.
49. Bonardi, A; Clifford, CJ; Hadar, N. A Structured Approach Using the Systematic Review Data Repository (SRDR): Building the Evidence for Oral Health Interventions in the Population With Intellectual and Developmental Disability. EVALUATION REVIEW 2017. http://dx.doi.org/10.1177/0193841X16664811.
    1. Reason: Not primary an oral health topic. Not a SR.
50. Bondemark, L; Lilja-Karlander, L. A systematic review of Swedish research in orthodontics during the past decade. ACTA ODONTOLOGICA SCANDINAVICA 2004. http://dx.doi.org/10.1080/00016350310008148.
    1. Reason: Not primary an oral health topic.
51. Boren, SA; Gunlock, TL; Schaefer, J; Albright, A. Reducing risks in diabetes self-management - A systematic review of the literature. DIABETES EDUCATOR 2007. http://dx.doi.org/10.1177/0145721707309809.
    1. Reason: Not primary an oral health topic.
52. Boyland, E; McGale, L; Maden, M; Hounsome, J; Boland, A; Angus, K; Jones, A. Association of Food and Nonalcoholic Beverage Marketing With Children and Adolescents' Eating Behaviors and Health A Systematic Review and Meta-analysis. JAMA PEDIATRICS 2022. http://dx.doi.org/10.1001/jamapediatrics.2022.1037.
    1. Reason: Not primary an oral health topic.
53. Boyland, E; McGale, L; Maden, M; Hounsome, J; Boland, A; Jones, A. Systematic review of the effect of policies to restrict the marketing of foods and non-alcoholic beverages to which children are exposed. OBESITY REVIEWS 2022. http://dx.doi.org/10.1111/obr.13447.
    1. Reason: Not primary an oral health topic.
54. Brighenti, FL; Salvador, MJ; Delbem, ACB; Delbem, ACB; Oliveira, MAC; Soares, CP; Freitas, LSF; Koga-Ito, CY. Systematic Screening of Plant Extracts from the Brazilian Pantanal with Antimicrobial Activity against Bacteria with Cariogenic Relevance. CARIES RESEARCH 2014. http://dx.doi.org/10.1159/000357225.
    1. Reason: Not a SR.
55. Brown, JVE; Crampton, PES; Finn, GM; Morgan, JE. From the sticky floor to the glass ceiling and everything in between: protocol for a systematic review of barriers and facilitators to clinical academic careers and interventions to address these, with a focus on gender inequality. SYSTEMATIC REVIEWS 2020. http://dx.doi.org/10.1186/s13643-020-1286-z.
    1. Reason: Not primary an oral health topic.
56. Bryce, M; Zahra, D; Burns, L; Hanks, S; Gale, T. Progress and challenges in the harmonisation of European undergraduate dental education: A systematic literature review with narrative synthesis. EUROPEAN JOURNAL OF DENTAL EDUCATION. http://dx.doi.org/10.1111/eje.12860.
    1. Reason: Not primary an oral health topic.
57. Buchalla, W; Attin, T. External bleaching therapy with activation by heat, light or laser - A systematic review. DENTAL MATERIALS 2007. http://dx.doi.org/10.1016/j.dental.2006.03.018
    1. Reason: Primary studies and review(s) were included in the assessment.
58. Buckley, S; Coleman, J; Davison, I; Khan, KS; Zamora, J; Malick, S; Morley, D; Pollard, D; Ashcroft, T; Popovic, C; Sayers, J. The educational effects of portfolios on undergraduate student learning: A Best Evidence Medical Education (BEME) systematic review. BEME Guide No. 11. MEDICAL TEACHER 2009. http://dx.doi.org/10.1080/01421590902889897.
    1. Reason: Not primary an oral health topic.
59. Bunevicius, A. The Association of Digit Ratio (2D:4D) with Cancer: A Systematic Review and Meta-Analysis. DISEASE MARKERS 2018. http://dx.doi.org/10.1155/2018/7698193.
    1. Reason: Not primary an oral health topic.
60. Bussadori, SK; Motta, LJ; Horliana, ACRT; Santos, EM; Martimbianco, ALC. The Current Trend in Management of Bruxism and Chronic Pain: An Overview of Systematic Reviews. JOURNAL OF PAIN RESEARCH 2020. http://dx.doi.org/10.2147/JPR.S268114.
    1. Reason: Not a SR.
61. Carreras-Torras, C; Gay-Escoda, C. Techniques for early diagnosis of oral squamous cell carcinoma: Systematic review. MEDICINA ORAL PATOLOGIA ORAL Y CIRUGIA BUCAL 2015. http://dx.doi.org/10.4317/medoral.20347.
    1. Reason: Primary studies and review(s) were included in the assessment.
62. Carson, SJ; Abuhaloob, L; Richards, D; Hector, MP; Freeman, R. The relationship between childhood body weight and dental caries experience: an umbrella systematic review protocol. SYSTEMATIC REVIEWS 2017. http://dx.doi.org/10.1186/s13643-017-0610-8.
    1. Reason: Not a SR.
63. Ceron, L; Pacheco, M; Gaete, AD; Torres, WB; Rubio, DA. Therapies for sleep bruxism in dentistry: A critical evaluation of systematic reviews. DENTAL AND MEDICAL PROBLEMS. http://dx.doi.org/10.17219/dmp/156400.
    1. Reason: Not a SR.
64. Champarnaud, M; Villars, H; Girard, P; Brechemier, D; Balardy, L; Nourhashempi, F. Effectiveness of Therapeutic Patient Education Interventions for Older Adults with Cancer: A Systematic Review. JOURNAL OF NUTRITION HEALTH & AGING 2020. http://dx.doi.org/10.1007/s12603-020-1395-3.
    1. Reason: Not primary an oral health topic.
65. Chang, CM; Corey, CG; Rostron, BL; Apelberg, BJ. Systematic review of cigar smoking and all cause and smoking related mortality. BMC PUBLIC HEALTH 2015. http://dx.doi.org/10.1186/s12889-015-1617-5.
    1. Reason: Not primary an oral health topic.
66. Chapireau, D; Adlam, D; Cameron, M; Thompson, M. Paraneoplastic syndromes in patients with primary oral cancers: a systematic review. BRITISH JOURNAL OF ORAL & MAXILLOFACIAL SURGERY 2010. http://dx.doi.org/10.1016/j.bjoms.2009.08.025.
    1. Reason: Unclear whether only primary studies were included in the assessment
67. Chavan, AS; Al Muderis, M; Tetsworth, K; Rustamov, ID; Hoellwarth, JS. Residual Amputee Limb Segment Lengthening: A Systematic Review. JOURNAL OF LIMB LENGTHENING & RECONSTRUCTION. 2022. http://dx.doi.org/10.4103/jllr.jllr_17_22.
    1. Reason: Not primary an oral health topic.
68. Chen, P; Wu, H; Yao, HL; Zhang, JS; Fan, WY; Chen, Z; Su, WW; Wang, YG; Li, PB. Multi-Omics Analysis Reveals the Systematic Relationship Between Oral Homeostasis and Chronic Sleep Deprivation in Rats. FRONTIERS IN IMMUNOLOGY 2022. http://dx.doi.org/10.3389/fimmu.2022.847132.
    1. Reason: Not a SR.
69. Chen, R; Santo, K; Wong, G; Sohn, W; Spallek, H; Chow, C; Irving, M. Mobile Apps for Dental Caries Prevention: Systematic Search and Quality Evaluation. JMIR MHEALTH AND UHEALTH 2021. http://dx.doi.org/10.2196/19958.
    1. Reason: Not a SR.
70. Chen, YW; Chen, XC; Yu, HX; Zhou, HB; Xu, S. Oral Microbiota as Promising Diagnostic Biomarkers for Gastrointestinal Cancer: A Systematic Review. ONCOTARGETS AND THERAPY 2019. http://dx.doi.org/10.2147/OTT.S230262.
    1. Reason: Not primary an oral health topic.
71. Chong, E; Pelletier, MH; Mobbs, RJ; Walsh, WR. The design evolution of interbody cages in anterior cervical discectomy and fusion: a systematic review. BMC MUSCULOSKELETAL DISORDERS 2015. http://dx.doi.org/10.1186/s12891-015-0546-x
    1. Reason: Not primary an oral health topic.
72. Chotai, PN; Nollan, R; Huang, EY; Gosain, A Surgical informed consent in children: a systematic review. JOURNAL OF SURGICAL RESEARCH 2017.  http://dx.doi.org/10.1016/j.jss.2017.02.047.
    1. Reason: Not primary an oral health topic.
73. Chung, CJ; Long, HY. Systematic strontium substitution in hydroxyapatite coatings on titanium via micro-arc treatment and their osteoblast/osteoclast responses. ACTA BIOMATERIALIA 2011. http://dx.doi.org/10.1016/j.actbio.2011.07.004.
    1. Reason: Not a SR.
74. Cooper, B; Behnke, NL; Cronk, R; Anthonj, C; Shackelford, BB; Tu, R; Bartram, J. Environmental health conditions in the transitional stage of forcible displacement: A systematic scoping review. SCIENCE OF THE TOTAL ENVIRONMENT 2021. http://dx.doi.org/10.1016/j.scitotenv.2020.143136.
    1. Reason: Not primary an oral health topic.
75. Cortela, DCB; de Souza, AL; Virmond, MCL; Ignotti, E. Inflammatory Mediators of Leprosy Reactional Episodes and Dental Infections: A Systematic Review. MEDIATORS OF INFLAMMATION 2015. http://dx.doi.org/10.1155/2015/548540.
    1. Reason: Not primary an oral health topic.
76. Creugers, NHJ; Kreulen, CM. Systematic review of 10 years of systematic reviews in prosthodontics. INTERNATIONAL JOURNAL OF PROSTHODONTICS 2003.
    1. Reason: Review of SRs.
77. Critchley, JA; Unal, B. Health effects associated with smokeless tobacco: a systematic review. THORAX 2003. http://dx.doi.org/10.1136/thorax.58.5.435.
    1. Reason: Not primary an oral health topic.
78. Cotti, E; Arrica, M; Di Lenarda, A; Serri, SB; Bassareo, P; Padeletti, L; Mercuro, G. The perioperative dental screening and management of patients undergoing cardiothoracic, vascular surgery and other cardiovascular invasive procedures: A systematic review. EUROPEAN JOURNAL OF PREVENTIVE CARDIOLOGY. http://dx.doi.org/10.1177/2047487316682348.
    1. Reason: Not primary an oral health topic.
79. Cueno, ME; Ochiai, K. Gingival Periodontal Disease (PD) Level-Butyric Acid Affects the Systematic Blood and Brain Organ: Insights Into the Systemic Inflammation of Periodontal Disease. FRONTIERS IN IMMUNOLOGY 2018. http://dx.doi.org/10.3389/fimmu.2018.01158.
    1. Reason: Not a SR.
80. Cunningham, A; McPolin, O; Fallis, R; Coyle, C; Best, P; McKenna, G. A systematic review of the use of virtual reality or dental smartphone applications as interventions for management of paediatric dental anxiety. BMC ORAL HEALTH 2021. http://dx.doi.org/10.1186/s12903-021-01602-3.
    1. Reason: Unclear whether only primary studies were included the in assessment.
81. Cushley, S; Duncan, HF; Lundy, FT; Nagendrababu, V; Clarke, M; El Karim, I. Outcomes reporting in systematic reviews on vital pulp treatment: A scoping review for the development of a core outcome set. INTERNATIONAL ENDODONTIC JOURNAL 2022. http://dx.doi.org/10.1111/iej.13785.
    1. Reason: Not a SR.
82. Cushley, S; McLister, C; Lappin, MJ; Harrington, M; Nagendrababu, V; Duncan, HF; El Karim, I. Outcomes reporting in systematic reviews on revitalization: A scoping review for the development of a core outcome set. INTERNATIONAL ENDODONTIC JOURNAL 2022. http://dx.doi.org/10.1111/iej.13829.
    1. Reason: Not a SR.
83. da-Silva-Domingues, H; del-Pino-Casado, R; Palomino-Moral, PA; Martinez, CL; Moreno-Camara, S; Frias-Osuna, A. Relationship between sense of coherence and health-related behaviours in adolescents and young adults: a systematic review. BMC PUBLIC HEALTH 2022. http://dx.doi.org/10.1186/s12889-022-12816-7.
    1. Reason: Not primary an oral health topic.
84. Dai, JW; Fu, YF; Chen, DM; Sun, ZY.A novel and injectable strontium-containing hydroxyapatite bone cement for bone substitution: A systematic evaluation. MATERIALS SCIENCE AND ENGINEERING C-MATERIALS FOR BIOLOGICAL APPLICATIONS 2021. http://dx.doi.org/10.1016/j.msec.2021.112052.
    1. Reason: Not a SR.
85. Dallman, AR; Artis, J; Watson, L; Wright, S. Systematic Review of Disparities and Differences in the Access and Use of Allied Health Services Amongst Children with Autism Spectrum Disorders. JOURNAL OF AUTISM AND DEVELOPMENTAL DISORDERS 2021. http://dx.doi.org/10.1007/s10803-020-04608-y.
    1. Reason: Not primary an oral health topic.
86. Dallora, AL; Anderberg, P; Kvist, O; Mendes, E; Ruiz, SD; Berglund, JS. Bone age assessment with various machine learning techniques: A systematic literature review and meta-analysis. PLOS ONE 2019. http://dx.doi.org/10.1371/journal.pone.0220242.
    1. Reason: Not primary an oral health topic.
87. De Buitrago, JG; Avila-Ortiz, G; Elangovan, S. Quality assessment of systematic reviews on alveolar ridge preservation. JOURNAL OF THE AMERICAN DENTAL ASSOCIATION 2013. http://dx.doi.org/10.14219/jada.archive.2013.0070.
    1. Reason: Not a SR.
88. de Lima, ACB; dos Santos, DCM; de Almeida, SL; da Silva, EL; Pereira, EBF. Hybrid education in healthcare education : a systematic review. REVISTA CUIDARTE. http://dx.doi.org/10.15649/cuidarte.2051.
    1. Reason: Not primary an oral health topic.
89. de Luna, CJMM; Vitus, K; Torslev, MK; Krasnik, A; Jervelund, SS. Ethnic inequalities in child and adolescent health in the Scandinavian welfare states: The role of parental socioeconomic status - a systematic review. SCANDINAVIAN JOURNAL OF PUBLIC HEALTH 2019. http://dx.doi.org/10.1177/1403494818779853.
    1. Reason: Not primary an oral health topic.
90. de Medeiros, MMD; Carletti, TM; Magno, MB; Maia, LC; Cavalcanti, YW; Rodrigues-Garcia, RCM. Does the institutionalization influence elderly's quality of life? A systematic review and meta-analysis. BMC GERIATRICS 2020. http://dx.doi.org/10.1186/s12877-020-1452-0.
    1. Reason: Not primary an oral health topic.
91. de Oliveira-Neto, OB; Barbosa, FT; de Sousa-Rodrigues, CF; de Lima, FJC. Risk of bias assessment of systematic reviews regarding dental implant placement in smokers: An umbrella systematic review. JOURNAL OF PROSTHETIC DENTISTRY 2018. http://dx.doi.org/10.1016/j.prosdent.2017.12.026.
    1. Reason: Not a SR.
92. de Oliveira-Neto, OB; Barbosa, FT; de Sousa-Rodrigues, CF; de Lima, FJC. Quality assessment of systematic reviews regarding immediate placement of dental implants into infected sites: An overview. JOURNAL OF PROSTHETIC DENTISTRY 2017. http://dx.doi.org/10.1016/j.prosdent.2016.09.007.
    1. Reason: Not a SR.
93. de Oliveira-Neto, OB; Santos, IO; Barbosa, FT; de Sousa-Rodrigues, CF; de Lima, FJC. Quality assessment of systematic reviews regarding dental implant placement on diabetic patients: an overview of systematic reviews. MEDICINA ORAL PATOLOGIA ORAL Y CIRUGIA BUCAL  2019. http://dx.doi.org/10.4317/medoral.22955.
    1. Reason: Not a SR.
94. Dixon, CJ; Knight, T; Binns, E; Ihaka, B; O'Brien, D. Clinical measures of balance in people with type two diabetes: A systematic literature review. GAIT & POSTURE 2017. http://dx.doi.org/10.1016/j.gaitpost.2017.08.022.
    1. Reason: Not primary an oral health topic.
95. do Amaral, GS; Negrini, T; Maltz, M; Arthur, RA. Restorative materials containing antimicrobial agents: is there evidence for their antimicrobial and anticaries effects? A systematic review. AUSTRALIAN DENTAL JOURNAL 2016. http://dx.doi.org/10.1111/adj.12338.
    1. Reason: Primary studies and review(s) were included in the assessment.
96. dos Santos, MBF; Agostini, BA; Bassani, R; Pereira, GKR; Sarkis-Onofre, R. Protocol registration improves reporting quality of systematic reviews in dentistry. BMC MEDICAL RESEARCH METHODOLOGY 2020. http://dx.doi.org/10.1186/s12874-020-00939-7.
    1. Reason: Not a SR.
97. Dotto, L; Lemes, LTO; Spazzin, AO; Sousa, YTCS; Pereira, GKR; Bacchi, A; Sarkis-Onofre, R. Acceptance of systematic reviews as Master/PhD theses in Brazilian graduate programs in dentistry. JOURNAL OF EVIDENCE BASED MEDICINE 2020. http://dx.doi.org/10.1111/jebm.12382.
    1. Reason: Not a SR.
98. Dreiseidler, T; Lentzen, MP; Zirk, M; Safi, AF; Zoeller, JE; Kreppel, M. Systematic three-dimensional analysis of wafer-based maxillary repositioning procedures in orthognathic surgery. JOURNAL OF CRANIO-MAXILLOFACIAL SURGERY 2017. http://dx.doi.org/10.1016/j.jcms.2017.08.022.
    1. Reason: Not a SR.
99. Edelhoff, D; Prandtner, O; Pour, RS; Wichelhaus, A; Liebermann, A. Systematic development of esthetics and function in a young patient with maxillary dental aplasia. JOURNAL OF ESTHETIC AND RESTORATIVE DENTISTRY 2017. http://dx.doi.org/10.1111/jerd.12315.
    1. Reason: Not a SR.
100. Edmonds, CJ; Foglia, E; Booth, P; Fu, CHY; Gardner, M. Dehydration in older people: A systematic review of the effects of dehydration on health outcomes, healthcare costs and cognitive performance. ARCHIVES OF GERONTOLOGY AND GERIATRICS 2021. http://dx.doi.org/10.1016/j.archger.2021.104380.
     1. Reason: Not primary an oral health topic.
101. Edvinsson, J; Rahm, M; Trinks, A; Hoglund, PJ. Senior Alert: A Quality Registry to Support a Standardized, Structured, and Systematic Preventive Care Process for Older Adults. QUALITY MANAGEMENT IN HEALTH CARE 2015. http://dx.doi.org/10.1097/QMH.0000000000000058.
     1. Reason: Not primary an oral health topic. Not a SR.
102. Eguren, M; Holguin, A; Diaz, K; Vidalon, J; Linan, C; Pacheco-Pereira, C; Vich, MOL. Can gray values be converted to Hounsfield units? A systematic review. DENTOMAXILLOFACIAL RADIOLOGY 2022. http://dx.doi.org/10.1259/dmfr.20210140.
     1. Reason: Not primary an oral health topic.
103. Elangovan, S; Avila-Ortiz, G; Johnson, GK; Karimbux, NY; Allareddy, V. Quality Assessment of Systematic Reviews on Periodontal Regeneration in Humans. JOURNAL OF PERIODONTOLOGY 2013. http://dx.doi.org/10.1902/jop.2012.120021.
     1. Reason: Not a SR.
104. Elangovan, S; Mawardi, HH; Karimbux, NY. Quality Assessment of Systematic Reviews on Short Dental Implants. JOURNAL OF PERIODONTOLOGY 2013. http://dx.doi.org/10.1902/jop.2012.120317.
     1. Reason: Not a SR.
105. Elkordy, SA; Palomo, L; Palomo, JM; Mostafa, YA. Do fixed orthodontic appliances adversely affect the periodontium? A systematic review of systematic reviews. SEMINARS IN ORTHODONTICS 2019. http://dx.doi.org/10.1053/j.sodo.2019.05.005
     1. Reason: Review of SRs.
106. Ellis-Smith, C; Evans, CJ; Bone, AE; Henson, LA; Dzingina, M; Kane, PM; Higginson, IJ; Daveson, BA. Measures to assess commonly experienced symptoms for people with dementia in long-term care settings: a systematic review. BMC MEDICINE 2016. http://dx.doi.org/10.1186/s12916-016-0582-x.
     1. Reason: Not primary an oral health topic.
107. Elsman, EBM; Al Baaj, M; van Rens, GHMB; Sijbrandi, W; van den Broek, EGC; van der Aa, HPA; Schakel, W; Heymans, MW; de Vries, R; Vervloed, MPJ; Steenbergen, B; van Nispen, RMA. Interventions to improve functioning, participation, and quality of life in children with visual impairment: a systematic review. SURVEY OF OPHTHALMOLOGY 2019. http://dx.doi.org/10.1016/j.survophthal.2019.01.010.
     1. Reason: Not primary an oral health topic.
108. Erickson, J; Sadeghirad, B; Lytvyn, L; Slavin, J; Johnston, BC. The Scientific Basis of Guideline Recommendations on Sugar Intake A Systematic Review. ANNALS OF INTERNAL MEDICINE 2017. http://dx.doi.org/10.7326/M16-2020.
     1. Reason: Not primary an oral health topic.
109. Etchebarne, M; Fricain, JC; Kerdjoudj, H; Di Pietro, R; Wolbank, S; Gindraux, F; Fenelon, M. Use of Amniotic Membrane and Its Derived Products for Bone Regeneration: A Systematic Review. FRONTIERS IN BIOENGINEERING AND BIOTECHNOLOGY 2021. http://dx.doi.org/10.3389/fbioe.2021.661332.
     1. Reason: Not primary an oral health topic.
110. Evans, K; Stone, V; Chen, L; Ge, XC; Xu, P. Systematic study of genes influencing cellular chain length in Streptococcus sanguinis. MICROBIOLOGY-SGM 2014. http://dx.doi.org/10.1099/mic.0.071688-0.
     1. Reason: Not a SR.
111. Faggion, CM; Atieh, M; Zanicotti, DG. Reporting of sources of funding in systematic reviews in periodontology and implant dentistry. BRITISH DENTAL JOURNAL 2014. http://dx.doi.org/10.1038/sj.bdj.2014.47.
     1. Reason: Not a SR.
112. Faggion, CM; Atieh, MA; Park, S. Search strategies in systematic reviews in periodontology and implant dentistry. JOURNAL OF CLINICAL PERIODONTOLOGY 2013. http://dx.doi.org/10.1111/jcpe.12132.
     1. Reason: Not a SR.
113. Faggion, CM; Chambrone, L; Gondim, V; Schmitter, M; Tu, YK. Comparison of the effects of treatment of peri-implant infection in animal and human studies: systematic review and meta-analysis. CLINICAL ORAL IMPLANTS RESEARCH 2010. http://dx.doi.org/10.1111/j.1600-0501.2009.01753.x.
     1. Reason: Unclear whether only primary studies were included the in assessment.
114. Faggion, CM; Cullinan, MP; Atieh, M; Wasiaksystemic, J. An overview of systematic reviews of the use of systemic antimicrobials for the treatment of periodontitis. BRITISH DENTAL JOURNAL 2014. http://dx.doi.org/10.1038/sj.bdj.2014.909.s.
     1. Reason: Review of SRs.
115. Faggion, CM; Giannakopoulos, NN. Critical appraisal of systematic reviews on the effect of a history of periodontitis on dental implant loss. JOURNAL OF CLINICAL PERIODONTOLOGY 2013. http://dx.doi.org/10.1111/jcpe.12096.
     1. Reason: Not a SR.
116. Faggion, CM; Huda, F; Wasiak, J. Use of methodological tools for assessing the quality of studies in periodontology and implant dentistry: a systematic review. JOURNAL OF CLINICAL PERIODONTOLOGY 2014. http://dx.doi.org/10.1111/jcpe.12251.
     1. Reason: Not primary an oral health topic.
117. Faggion, CM; Listl, S; Alarcon, MA. Is the evaluation of risk of bias in periodontology and implant dentistry comprehensive? A systematic review. JOURNAL OF CLINICAL PERIODONTOLOGY 2015. http://dx.doi.org/10.1111/jcpe.12394.
     1. Reason: Not primary an oral health topic.
118. Faggion, CM; Listl, S; Giannakopoulos, NN. The methodological quality of systematic reviews of animal studies in dentistry. VETERINARY JOURNAL 2012. http://dx.doi.org/10.1016/j.tvjl.2011.08.006.
     1. Reason: Not primary an oral health topic.
119. Faggion, CM; Liu, J; Huda, F; Atieh, M. Assessment of the quality of reporting in abstracts of systematic reviews with meta-analyses in periodontology and implant dentistry. JOURNAL OF PERIODONTAL RESEARCH 2014. http://dx.doi.org/10.1111/jre.12092.
     1. Reason: Not a SR.
120. Faggion, CM; Monje, A; Wasiak, J. Appraisal of systematic reviews on the management of peri-implant diseases with two methodological tools. JOURNAL OF CLINICAL PERIODONTOLOGY 2018. http://dx.doi.org/10.1111/jcpe.12893.
     1. Reason: Not a SR.
121. Fakhri, Y; Mohseni-Bandpei, A; Conti, GO; Ferrante, M; Cristaldi, A; Jeihooni, AK; Dehkordi, MK; Alinejad, A; Rasoulzadeh, H; Mohseni, SM; Sarkhosh, M; Keramati, H; Moradi, B; Amanidaz, N; Baninameh, Z. Systematic review and health risk assessment of arsenic and lead in the fished shrimps from the Persian gulf. FOOD AND CHEMICAL TOXICOLOGY. http://dx.doi.org/10.1016/j.fct.2018.01.046.
     1. Reason: Not primary an oral health topic.
122. Fakhruddin, KS; Samaranayake, LP; Buranawat, B; Ngo, H. Oro-facial mucocutaneous manifestations of Coronavirus Disease-2019 (COVID-19): A systematic review. PLOS ONE 2022. http://dx.doi.org/10.1371/journal.pone.0265531.
     1. Reason: Not primary an oral health topic.
123. Fathi, A; Ebadian, B; Dezaki, SN; Mardasi, N; Mosharraf, R; Isler, S; Tabatabaei, SS. An Umbrella Review of Systematic Reviews and Meta-Analyses Evaluating the Success Rate of Prosthetic Restorations on Endodontically Treated Teeth. INTERNATIONAL JOURNAL OF DENTISTRY 2022. http://dx.doi.org/10.1155/2022/4748291.
     1. Reason: Review of SRs.
124. Feinberg, AE; Chesney, TR; Srikandarajah, S; Acuna, SA; McLeod, RS. Opioid Use After Discharge in Postoperative Patients A Systematic Review. ANNALS OF SURGERY 2018. http://dx.doi.org/10.1097/SLA.0000000000002591.
     1. Reason: Not primary an oral health topic.
125. Feng, Y; Yang, DS; Tang, HB; Ding, YS; Li, XG. Efficacy and safety of cisplatin for the management of adult patients with oral cancer A protocol for systematic review. MEDICINE 2019. http://dx.doi.org/10.1097/MD.0000000000018210.
     1. Reason: Not a SR.
126. Feres, M; Duarte, PM; Figueiredo, LC; Goncalves, C; Shibli, J; Retamal-Valdes, B. Systematic and scoping reviews to assess biological parameters. JOURNAL OF CLINICAL PERIODONTOLOGY 2022. http://dx.doi.org/10.1111/jcpe.13681.
     1. Reason: Not a SR.
127. Fernandez-Ferrer, L; Montiel-Company, JM; Pinho, T; Almerich-Silla, JM; Bellot-Arcis, C. Effects of mandibular setback surgery on upper airway dimensions and their influence on obstructive sleep apnoea - A systematic review. JOURNAL OF CRANIO-MAXILLOFACIAL SURGERY 2015. http://dx.doi.org/10.1016/j.jcms.2014.11.017.
     1. Reason: Primary studies and review(s) were included in the assessment.
128. Fieux, M; Le Quellec, S; Battier, S; Coste, A; Louis, B; Giroudon, C; Nourredine, M; Bequignon, E. FcRn as a Transporter for Nasal Delivery of Biologics: A Systematic Review. INTERNATIONAL JOURNAL OF MOLECULAR SCIENCES 2021. http://dx.doi.org/10.3390/ijms22126475.
     1. Reason: Not primary an oral health topic.
129. Fijacko, N; Gosak, L; Cilar, L; Novsak, A; Creber, RM; Skok, P; Stiglic, G. The Effects of Gamification and Oral Self-Care on Oral Hygiene in Children: Systematic Search in App Stores and Evaluation of Apps. JMIR MHEALTH AND UHEALTH 2020. http://dx.doi.org/10.2196/16365.
     1. Reason: Not a SR.
130. Fiorino, S; Bacchi-Reggiani, L; De Biase, D; Fornelli, A; Masetti, M; Tura, A; Grizzi, F; Zanello, M; Mastrangelo, L; Lombardi, R; Acquaviva, G; di Tommaso, L; Bondi, A; Visani, M; Sabbatani, S; Pontoriero, L; Fabbri, C; Cuppini, A; Pession, A; Jovine, E. Possible association between hepatitis C virus and malignancies different from hepatocellular carcinoma: A systematic review. WORLD JOURNAL OF GASTROENTEROLOGY 2015. http://dx.doi.org/10.3748/wjg.v21.i45.12896.
     1. Reason: Not primary an oral health topic.
131. Fite, RO; Kooti, W; Azeze, GA; Tesfaye, B; Hagisso, SN. Seroprevalence and factors associated with hepatitis B virus infection in blood donors in Ethiopia: a systematic review and meta-analysis. ARCHIVES OF VIROLOGY 2020. http://dx.doi.org/10.1007/s00705-020-04591-w.
     1. Reason: Not primary an oral health topic.
132. Fleming, PS; DiBiase, AT. Systematic reviews in orthodontics: What have we learned?. INTERNATIONAL DENTAL JOURNAL 2008. https://doi.org/10.1111/j.1875-595X.2008.tb00171.x.
     1. Reason: Not a SR.
133. Fleming, PS; Seehra, J; Polychronopoulou, A; Fedorowicz, Z; Pandis, N. A PRISMA assessment of the reporting quality of systematic reviews in orthodontics. ANGLE ORTHODONTIST 2013 http://dx.doi.org/10.2319/032612-251.1.
     1. Reason: Not a SR.
134. Fleming, PS; Seehra, J; Polychronopoulou, A; Fedorowicz, Z; Pandis, N. Cochrane and non-Cochrane systematic reviews in leading orthodontic journals: a quality paradigm? EUROPEAN JOURNAL OF ORTHODONTICS 2013. http://dx.doi.org/10.1093/ejo/cjs016.
     1. Reason: Not a SR.
135. Flett, K; Clark-Carter, D; Grogan, S; Davey, R. How effective are physical appearance interventions in changing smoking perceptions, attitudes and behaviours? A systematic review. TOBACCO CONTROL 2013. http://dx.doi.org/10.1136/tobaccocontrol-2011-050236.
     1. Reason: Not primary an oral health topic.
136. Flores-Mir, C; Major, MP; Major, PW. Search and selection methodology of systematic reviews in orthodontics (2000-2004). AMERICAN JOURNAL OF ORTHODONTICS AND DENTOFACIAL ORTHOPEDICS. 2006. http://dx.doi.org/10.1016/j.ajodo.2006.02.028.
     1. Reason: Not a SR.
137. Fortuna, G; Aria, M; Piscitelli, A; Mignogna, MD; Klasser, GD. Global research trends in complex oral sensitivity disorder: A systematic bibliometric analysis of the framework. JOURNAL OF ORAL PATHOLOGY & MEDICINE 2020. http://dx.doi.org/10.1111/jop.13076.
     1. Reason: Not a SR.
138. Fortuna, G; Aria, M; Piscitelli, A; Mignogna, MD; Klasser, GD. Global research trends in complex oral sensitivity disorder: A systematic bibliometric analysis of the structures of knowledge. JOURNAL OF ORAL PATHOLOGY & MEDICINE 2020. http://dx.doi.org/10.1111/jop.13077.
     1. Reason: Not a SR.
139. Fransson, H; Wolf, E; Petersson, K. Formation of a hard tissue barrier after experimental pulp capping or partial pulpotomy in humans: an updated systematic review. INTERNATIONAL ENDODONTIC JOURNAL 2016. http://dx.doi.org/10.1111/iej.12480.
     1. Reason: Unclear whether only primary studies were included the in assessment.
140. Frisch, E; Wild, V; Ratka-Kruger, P; Vach, K; Sennhenn-Kirchner, S. Long-termresults of implants and implant-supportedprostheses under systematic supportive implant therapy: A retrospective25-yearstudy. CLINICAL IMPLANT DENTISTRY AND RELATED RESEARCH 2020. http://dx.doi.org/10.1111/cid.12944.
     1. Reason: Not a SR.
141. Fritz, R; Bauer, JG; Spackman, SS; Bains, AK; Jetton-Rangel, J. CONFIDENCE IN OUTCOME ESTIMATES FROM SYSTEMATIC REVIEWS USED IN INFORMED CONSENT. JOURNAL OF EVIDENCE-BASED DENTAL PRACTICE 2016. http://dx.doi.org/10.1016/j.jebdp.2016.08.001.
     1. Reason: Not a SR.
142. Fuentes, R; Arias, A; Lezcano, MF; Saravia, D; Kuramochi, G; Dias, FJ. Systematic Standardized and Individualized Assessment of Masticatory Cycles Using Electromagnetic 3D Articulography and Computer Scripts. BIOMED RESEARCH INTERNATIONAL 2017. http://dx.doi.org/10.1155/2017/7134389.
     1. Reason: Not a SR.
143. Fugazza, A; Gaiani, F; Carra, MC; Brunetti, F; Levy, M; Sobhani, I; Azoulay, D; Catena, F; de'Angelis, GL; de'Angelis, N. Confocal Laser Endomicroscopy in Gastrointestinal and Pancreatobiliary Diseases: A Systematic Review and Meta-Analysis. BIOMED RESEARCH INTERNATIONAL 2016. http://dx.doi.org/10.1155/2016/4638683.
     1. Reason: Not primary an oral health topic.
144. Garcia-Gonzalez, M; Munoz, F; Gonzalez-Cantalapiedra, A; Lopez-Pena, M; Saulacic, N. Systematic Review and Quality Evaluation Using ARRIVE 2.0 Guidelines on Animal Models Used for Periosteal Distraction Osteogenesis. ANIMALS 2021. http://dx.doi.org/10.3390/ani11051233.
     1. Reason: Not primary an oral health topic.
145. Gargon, E; Gurung, B; Medley, N; Altman, DG; Blazeby, JM; Clarke, M; Williamson, PR. Choosing Important Health Outcomes for Comparative Effectiveness Research: A Systematic Review. PLOS ONE 2014. http://dx.doi.org/10.1371/journal.pone.0099111.
     1. Reason: Not primary an oral health topic.
146. Gartagani, Z; Doumas, S; Kyriakopoulou, A; Economopoulou, P; Psaltopoulou, T; Kotsantis, I; Sergentanis, TN; Psyrri, A. Lymph Node Ratio as a Prognostic Factor in Neck Dissection in Oral Cancer Patients: A Systematic Review and Meta-Analysis. CANCERS 2022. http://dx.doi.org/10.3390/cancers14184456.
     1. Reason: Not primary an oral health topic.
147. Gartenmann, SJ; Dorig, I; Sahrmann, P; Held, U; Walter, C; Schmidlin, PR. Influence of different post-interventional maintenance concepts on periodontal outcomes: an evaluation of three systematic reviews. BMC ORAL HEALTH 2016. http://dx.doi.org/10.1186/s12903-016-0244-6.
     1. Reason: Not a SR.
148. Gasparro, R; Bucci, R; De Rosa, F; Sammartino, G; Bucci, P; D'Anto, V; Marenzi, G. Effectiveness of surgical procedures in the acceleration of orthodontic tooth movement: Findings from systematic reviews and meta-analyses. JAPANESE DENTAL SCIENCE REVIEW 2022. http://dx.doi.org/10.1016/j.jdsr.2022.03.003.
     1. Reason: Not a SR.
149. Gaur, S; Agnihotri, R. Health Effects of Trace Metals in Electronic Cigarette Aerosolsa Systematic Review. BIOLOGICAL TRACE ELEMENT RESEARCH 2019. http://dx.doi.org/10.1007/s12011-018-1423-x.
     1. Reason: Not primary an oral health topic.
150. George, PP; Papachristou, N; Belisario, JM; Wang, W; Wark, PA; Cotic, Z; Rasmussen, K; Sluiter, R; Riboli-Sasco, E; Car, LT; Musulanov, EM; Molina, JA; Heng, BH; Zhang, YF; Wheeler, EL; Al Shorbaji, N; Majeed, A; Car, J. Online eLearning for undergraduates in health professions: A systematic review of the impact on knowledge, skills, attitudes and satisfaction. JOURNAL OF GLOBAL HEALTH 2014. http://dx.doi.org/10.7189/jogh.04.010406.
     1. Reason: Not primary an oral health topic.
151. Gevert, MV; Soares, R; Wambier, LM; Ribeiro, AE; Avais, LS; de Souza, JF; Chibinski, ACR. How is the quality of the available evidence on molar-incisor hypomineralization treatment? An overview of systematic reviews. CLINICAL ORAL INVESTIGATIONS 2022. http://dx.doi.org/10.1007/s00784-022-04612-9.
     1. Reason: Not a SR.
152. Ghasemiesfe, M; Barrow, B; Leonard, S; Keyhani, S; Korenstein, D. Association Between Marijuana Use and Risk of Cancer A Systematic Review and Meta-analysis. JAMA NETWORK OPEN 2019. http://dx.doi.org/10.1001/jamanetworkopen.2019.16318.
     1. Reason: Not primary an oral health topic.
153. Ghorbanian, A; Rashidian, A; Lankarani, KB; Kavosi, Z. The Prevalence and Determinants of Catastrophic Health Expenditures in Iran: A Systematic Review and Meta-Analysis. HEALTH SCOPE 2019. http://dx.doi.org/10.5812/jhealthscope.63210.
     1. Reason: Not primary an oral health topic.
154. Giannakoulas, DG; Koletsi, D; Tzanetakis, GN. Assessment of spin in abstracts of Endodontic Systematic Reviews with meta-analyses published between 2010 and 2022. Are we in need of more transparent interpretation of findings?. INTERNATIONAL ENDODONTIC JOURNAL 2022. http://dx.doi.org/10.1111/iej.13832
     1. Reason: Not a SR.
155. Gil, APS; Haas, OL; Machado-Fernandez, A; Munoz-Pereira, ME; Velasques, BD; da Rosa, BM; Favoreto, AXP; de Oliveira, RB. Antibiotic prophylaxis in orthognathic surgery: an overview of systematic reviews. BRITISH JOURNAL OF ORAL & MAXILLOFACIAL SURGERY 2021. http://dx.doi.org/10.1016/j.bjoms.2021.05.010.
     1. Reason: Not a SR.
156. Glenny, AM; Esposito, M; Coulthard, P; Worthington, HV. The assessment of systematic reviews in dentistry. EUROPEAN JOURNAL OF ORAL SCIENCES 2003. http://dx.doi.org/10.1034/j.1600-0722.2003.00013.
     1. Reason: Not a SR.
157. Gnanamanickam, ES; Teusner, DN; Arrow, PG; Brennan, DS. Dental insurance, service use and health outcomes in Australia: a systematic review. AUSTRALIAN DENTAL JOURNAL. http://dx.doi.org/10.1111/adj.12534.
     1. Reason: Not primary an oral health topic.
158. Gogos, C; Kodonas, K; Fardi, A; Economides, N. Top 100 cited systematic reviews and meta-analyses in dentistry. ACTA ODONTOLOGICA SCANDINAVICA 2020. http://dx.doi.org/10.1080/00016357.2019.1653495.
     1. Reason: Not a SR.
159. Gomes, RFT; Schuch, LF; Martins, MD; Honorio, EF; de Figueiredo, RM; Schmith, J; Machado, GN; Carrard, VC. Use of Deep Neural Networks in the Detection and Automated Classification of Lesions Using Clinical Images in Ophthalmology, Dermatology, and Oral Medicine-A Systematic Review. JOURNAL OF DIGITAL IMAGING. http://dx.doi.org/10.1007/s10278-023-00775-3.
     1. Reason: Not primary an oral health topic.
160. Gomez-Moreno, G; Aguilar-Salvatierra, A; Boquete-Castro, A; Guardia, J; Piattelli, A; Perrotti, V; Delgado-Ruiz, RA; Calvo-Guirado, JL. Outcomes of Topical Applications of Melatonin in Implant Dentistry: A Systematic Review. IMPLANT DENTISTRY 2015. http://dx.doi.org/10.1097/ID.0000000000000186.
     1. Reason: Primary studies and review(s) were included in the assessment.
161. Gonzalez-Moles, MA; Aguilar-Ruiz, M; Ramos-Garcia, P. Challenges in the Early Diagnosis of Oral Cancer, Evidence Gaps and Strategies for Improvement: A Scoping Review of Systematic Reviews. CANCERS 2022. http://dx.doi.org/10.3390/cancers14194967.
     1. Reason: Not a SR.
162. Gonzalez-Moles, MA; Keim-del Pino, C; Ramos-Garcia, P. Hallmarks of Cancer Expression in Oral Lichen Planus: A Scoping Review of Systematic Reviews and Meta-Analyses. INTERNATIONAL JOURNAL OF MOLECULAR SCIENCES 2022. http://dx.doi.org/10.3390/ijms232113099.
     1. Reason: Not a SR.
163. Gordon, SJ; Grimmer, K; Bradley, A; Direen, T; Baker, N; Marin, T; Kelly, MT; Gardner, S; Steffens, M; Burgess, T; Hume, C; Oliffe, JL. Health assessments and screening tools for adults experiencing homelessness: a systematic review. BMC PUBLIC HEALTH 2019. http://dx.doi.org/10.1186/s12889-019-7234-y.
     1. Reason: Not primary an oral health topic.
164. Griffin, SO; Naavaal, S; Scherrer, C; Patel, M; Chattopadhyay, S. Evaluation of School-Based Dental Sealant Programs: An Updated Community Guide Systematic Economic Review. AMERICAN JOURNAL OF PREVENTIVE MEDICINE 2017. http://dx.doi.org/10.1016/j.amepre.2016.10.004.
     1. Reason: Not primary an oral health topic.
165. Grusovin, MG; Coulthard, P; Worthington, HV; Esposito, M. Maintaining and recovering soft tissue health around dental implants: a Cochrane systematic review of randomised controlled clinical trials. EUROPEAN JOURNAL OF ORAL IMPLANTOLOGY 2008.
     1. Reason: Unclear whether only primary studies were included the in assessment.
166. Guida, A; Cecoro, G; Rullo, R; Laino, L; Del Fabbro, M; Annunziata, M. A Systematic Critical Appraisal of the Methodological Quality of Systematic Reviews on the Effect of Autologous Platelet Concentrates in the Treatment of Periodontal Intraosseous Defects. MATERIALS 2020. http://dx.doi.org/10.3390/ma13184180.
     1. Reason: Not a SR.
167. Haakenstad, A; Irvine, CMS; Knight, M; Bintz, C; Aravkin, AY; Zheng, P; Gupta, V; Abrigo, MRM; Abushouk, AI; Adebayo, OM; Agarwal, G; Alahdab, F; Al-Aly, Z; Alam, K; Alanzi, TM; Alcalde-Rabanal, JE; Alipour, V; Alvis-Guzman, N; Amit, AML; Andrei, CL; Andrei, T; Antonio, CAT; Arabloo, J; Aremu, O; Ayanore, MA; Banach, M; Barnighausen, TW; Barthelemy, CM; Bayati, M; Benzian, H; Berman, AE; Bienhoff, K; Bijani, A; Bikbov, B; Biondi, A; Boloor, A; Busse, R; Butt, ZA; Camera, LA; Campos-Nonato, IR; Cardenas, R; Carvalho, F; Chansa, C; Chattu, SK; Chattu, VK; Chu, DT; Dai, XC; Dandona, L; Dandona, R; Dangel, WJ; Daryani, A; De Neve, JW; Dhimal, M; Dipeolu, IO; Djalalinia, S; Do, HT; Doshi, CP; Doshmangir, L; Ehsani-Chimeh, E; El Tantawi, M; Fernandes, E; Fischer, F; Foigt, NA; Fomenkov, AA; Foroutan, M; Fukumoto, T; Fullman, N; Gad, MM; Ghadiri, K; Ghafourifard, M; Ghashghaee, A; Glucksman, T; Goudarzi, H; Das Gupta, R; Hamadeh, RR; Hamidi, S; Haro, JM; Hasanpoor, E; Hay, SI; Hegazy, MI; Heibati, B; Henry, NJ; Hole, MK; Hossain, N; Househ, M; Ilesanmi, OS; Imani-Nasab, MH; Irvani, SSN; Islam, SMS; Jahani, MA; Joshi, A; Kalhor, R; Kayode, GA; Khalid, N; Khatab, K; Kisa, A; Kochhar, S; Krishan, K; Defo, BK; Lal, DK; Lami, FH; Larsson, AO; Leasher, JL; LeGrand, KE; Lim, LL; Mahotra, NB; Majeed, A; Maleki, A; Manjunatha, N; Massenburg, BB; Mestrovic, T; Mini, GK; Mirica, A; Mirrakhimov, EM; Mohammad, Y; Mohammed, S; Mokdad, AH; Morrison, SD; Naghavi, M; Ndwandwe, DE; Negoi, I; Negoi, RI; Ngunjiri, JW; Nguyen, CT; Nigatu, YT; Onwujekwe, OE; Ortega-Altamirano, DV; Otstavnov, N; Otstavnov, SS; Owolabi, MO; Pakhare, AP; Pepito, VCF; Perico, N; Pham, HQ; Pigott, DM; Pokhrel, KN; Rabiee, M; Rabiee, N; Rahimi-Movaghar, V; Rawaf, DL; Rawaf, S; Rawal, L; Remuzzi, G; Renzaho, AMN; Resnikoff, S; Rezaei, N; Rezapour, A; Rickard, J; Roever, L; Sahu, M; Samy, AM; Sanabria, J; Santric-Milicevic, MM; Saraswathy, SYI; Seedat, S; Senthilkumaran, S; Servan-Mori, E; Shaikh, MA; Sheikh, A; Silva, DAS; Stein, C; Stein, DJ; Titova, MV; Topp, SM; Tovani-Palone, MR; Ullah, S; Unnikrishnan, B; Vacante, M; Valdez, PR; Vasankari, TJ; Venketasubramanian, N; Vlassov, V; Vos, T; Yearwood, JA; Yonemoto, N; Younis, MZ; Yu, CH; Zadey, S; Bin Zaman, S; Zerfu, TA; Zhang, ZJ; Ziapour, A; Zodpey, S; Lim, SS; Murray, CJL; Lozano, R. Measuring the availability of human resources for health and its relationship to universal health coverage for 204 countries and territories from 1990 to 2019: a systematic analysis for the Global Burden of Disease Study 2019. LANCET 2022. http://dx.doi.org/10.1016/S0140-6736(22)00532-3.
     1. Reason: Not primary an oral health topic. Not a SR.
168. Haas, OL; Guijarro-Martinez, R; Gil, APD; Meirelles, LD; Scolari, N; Munoz-Pereira, ME; Hernandez-Alfaro, F; de Oliveira, RB. Hierarchy of surgical stability in orthognathic surgery: overview of systematic reviews. INTERNATIONAL JOURNAL OF ORAL AND MAXILLOFACIAL SURGERY 2019. http://dx.doi.org/10.1016/j.ijom.2019.03.003.
     1. Reason: Review of SRs.
169. Halim, FC; Pesce, P; De Angelis, N; Benedicenti, S; Menini, M. Comparison of the Clinical Outcomes of Titanium and Zirconia Implant Abutments: A Systematic Review of Systematic Reviews. JOURNAL OF CLINICAL MEDICINE 2022. http://dx.doi.org/10.3390/jcm11175052.
     1. Reason: Review of SRs.
170. Hammel, C; Pandis, N; Pieper, D; Faggion, CM. Methodological assessment of systematic reviews of in-vitro dental studies. BMC MEDICAL RESEARCH METHODOLOGY 2022. http://dx.doi.org/10.1186/s12874-022-01575-z.
     1. Reason: Not a SR.
171. Hanks, S; Cotton, D; Spowart, L. Leadership in Dental Practice: a Three Stage Systematic Review and Narrative Synthesis. JOURNAL OF DENTISTRY 2020. http://dx.doi.org/10.1016/j.jdent.2020.103480.
     1. Reason: Not primary an oral health topic.
172. Harris, VC; Links, AR; Walsh, J; Schoo, DP; Lee, AH; Tunkel, DE; Boss, EF. A Systematic Review of Race/Ethnicity and Parental Treatment Decision-Making. CLINICAL PEDIATRICS 2018. http://dx.doi.org/10.1177/0009922818788307.
     1. Reason: Not primary an oral health topic.
173. Hashem, KM; He, FJ; MacGregor, GA. Systematic review of the literature on the effectiveness of product reformulation measures to reduce the sugar content of food and drink on the population's sugar consumption and health: a study protocol. BMJ OPEN 2016. http://dx.doi.org/10.1136/bmjopen-2016-011052.
     1. Reason: Not primary an oral health topic.
174. Hashem, KM; He, FJ; MacGregor, GA. Effects of product reformulation on sugar intake and healtha systematic review and meta-analysis. NUTRITION REVIEWS 2019. http://dx.doi.org/10.1093/nutrit/nuy015.
     1. Reason: Not primary an oral health topic.
175. Hasuike, A; Iguchi, S; Suzuki, D; Kawano, E; Sato, S. Systematic review and assessment of systematic reviews examining the effect of periodontal treatment on glycemic control in patients with diabetes. MEDICINA ORAL PATOLOGIA ORAL Y CIRUGIA BUCAL 2017. http://dx.doi.org/10.4317/medoral.21555.
     1. Reason: Review of SRs.
176. Hasuike, A; Ueno, D; Nagashima, H; Kubota, T; Tsukune, N; Watanabe, N; Sato, S. Methodological quality and risk-of-bias assessments in systematic reviews of treatments for peri-implantitis. JOURNAL OF PERIODONTAL RESEARCH 2019. http://dx.doi.org/10.1111/jre.12638.
     1. Reason: Not a SR.
177. Hayes, MJ; Cockrell, D; Smith, DR. A systematic review of musculoskeletal disorders among dental professionals. INTERNATIONAL JOURNAL OF DENTAL HYGIENE 2009. http://dx.doi.org/10.1111/j.1601-5037.2009.00395.x.
     1. Reason: Not primary an oral health topic.
178. He, J; Yarbrough, DK; Kreth, J; Anderson, MH; Shi, WY; Eckert, R. Systematic Approach to Optimizing Specifically Targeted Antimicrobial Peptides against Streptococcus mutans. ANTIMICROBIAL AGENTS AND CHEMOTHERAPY 2010. http://dx.doi.org/10.1128/AAC.01391-09.
     1. Reason: Not a SR.
179. Heiderich, CMC; Tedesco, TK; Netto, SS; de Sousa, RC; Allegrini, S; Mendes, FM; Gimenez, T. Methodological quality and risk of bias of systematic reviews about loading time of multiple dental implants in totally or partially edentulous patients: An umbrella systematic review. JAPANESE DENTAL SCIENCE REVIEW 2020. http://dx.doi.org/10.1016/j.jdsr.2020.09.004.
     1. Reason: Not a SR.
180. Helfand, M; Buckley, DI; Freeman, M; Fu, RW; Rogers, K; Fleming, C; Humphrey, LL. Emerging Risk Factors for Coronary Heart Disease: A Summary of Systematic Reviews Conducted for the US Preventive Services Task Force. ANNALS OF INTERNAL MEDICINE 2009. http://dx.doi.org/10.7326/0003-4819-151-7-200910060-00010.
     1. Reason: Not a SR.
181. Henry, JA; Muthu, MS; Swaminathan, K; Kirubakaran, R. Do Oral Health Educational Programmes for Expectant Mothers Prevent Early Childhood Caries? - A Systematic Review. ORAL HEALTH & PREVENTIVE DENTISTRY 2017. http://dx.doi.org/10.3290/j.ohpd.a38522.
     1. Reason: Not primary an oral health topic.
182. Hilmanto, D; Mawardi, F; Lestari, AS; Widiasta, A. Disease-Associated Systemic Complications in Childhood Nephrotic Syndrome: A Systematic Review. INTERNATIONAL JOURNAL OF NEPHROLOGY AND RENOVASCULAR DISEASE 2022. http://dx.doi.org/10.2147/IJNRD.S351053.
     1. Reason: Not primary an oral health topic.
183. Hollands, G; Usher-Smith, JJ; Hasan, R; Alexander, F; Clarke, N; Griffin, S. Visualising health risks with medical imaging for changing recipients' health behaviours and risk factors: Systematic review with meta-analysis. PLOS MEDICINE 2022. http://dx.doi.org/10.1371/journal.pmed.1003920.
     1. Reason: Not primary an oral health topic.
184. Hoodbhoy, Z; Jeelani, SM; Aziz, A; Habib, MI; Iqbal, B; Akmal, W; Siddiqui, K; Hasan, B; Leeflang, M; Das, JK. Machine Learning for Child and Adolescent Health: A Systematic Review. PEDIATRICS 2021. http://dx.doi.org/10.1542/peds.2020-011833.
     1. Reason: Not primary an oral health topic.
185. Huybrechts, I; Zouiouich, S; Loobuyck, A; Vandenbulcke, Z; Vogtmann, E; Pisanu, S; Iguacel, I; Scalbert, A; Indave, I; Smelov, V; Gunter, MJ; Michels, N. The Human Microbiome in Relation to Cancer Risk: A Systematic Review of Epidemiologic Studies. CANCER EPIDEMIOLOGY BIOMARKERS & PREVENTION 2020. http://dx.doi.org/10.1158/1055-9965.EPI-20-0288.
     1. Reason: Not primary an oral health topic.
186. Iftikhar, H; Awan, MO; Awan, MS; Mustafa, K; Das, JK; Ahmed, SK Role of Probiotics in Patients with Allergic Rhinitis: A Systematic Review of Systematic Reviews. INTERNATIONAL ARCHIVES OF OTORHINOLARYNGOLOGY 2022. http://dx.doi.org/10.1055/s-0042-1749370.
     1. Reason: Not primary an oral health topic. and Review of SRs.
187. Ijaz, S; Croucher, RE; Marinho, VCC. Systematic Reviews of Topical Fluorides for Dental Caries: A Review of Reporting Practice. CARIES RESEARCH 2010. http://dx.doi.org/10.1159/000322132.
     1. Reason: Not a SR.
188. Inchingolo, AD; Inchingolo, AM; Malcangi, G; Avantario, P; Azzollini, D; Buongiorno, S; Viapiano, F; Campanelli, M; Ciocia, AM; De Leonardis, N; de Ruvo, E; Ferrara, I; Garofoli, G; Montenegro, V; Netti, A; Palmieri, G; Mancini, A; Patano, A; Piras, F; Marinelli, G; Di Pede, C; Laudadio, C; Rapone, B; Hazballa, D; Corriero, A; Fatone, MC; Palermo, A; Lorusso, F; Scarano, A; Bordea, IR; Di Venere, D; Inchingolo, F; Dipalma, G. Effects of Resveratrol, Curcumin and Quercetin Supplementation on Bone Metabolism-A Systematic Review. NUTRIENTS 2022. http://dx.doi.org/10.3390/nu14173519.
     1. Reason: Not primary an oral health topic.
189. Inchingolo, F; Hazballa, D; Inchingolo, AD; Malcangi, G; Marinelli, G; Mancini, A; Maggiore, ME; Bordea, IR; Scarano, A; Farronato, M; Tartaglia, GM; Lorusso, F; Inchingolo, AM; Dipalma, G. Innovative Concepts and Recent Breakthrough for Engineered Graft and Constructs for Bone Regeneration: A Literature Systematic Review. MATERIALS 2022. http://dx.doi.org/10.3390/ma15031120.
     1. Reason: Not primary an oral health topic.
190. Jayaraman, J; Nagendrababu, V; Pulikkotil, SJ; Innes, NP. Critical appraisal of methodological quality of Systematic Reviews and Meta-analysis in Paediatric Dentistry journals. INTERNATIONAL JOURNAL OF PAEDIATRIC DENTISTRY 2018. http://dx.doi.org/10.1111/ipd.12414.
     1. Reason: Not a SR.
191. Jayawardena, R; Swarnamali, H; Ranasinghe, P; Misra, A. Health effects of coconut oil: Summary of evidence from systematic reviews and meta-analysis of interventional studies. DIABETES & METABOLIC SYNDROME-CLINICAL RESEARCH & REVIEWS 2021. http://dx.doi.org/10.1016/j.dsx.2021.02.032.
     1. Reason: Not a SR.
192. Jin, J; Bridges, SM. Educational Technologies in Problem-Based Learning in Health Sciences Education: A Systematic Review. JOURNAL OF MEDICAL INTERNET RESEARCH 2014. http://dx.doi.org/10.2196/jmir.3240.
     1. Reason: Not primary an oral health topic.
193. Jokstad, A; Gokce, M; Hjortsjo, C. A systematic review of the scientific documentation of fixed partial dentures made from fiber-reinforced polymer to replace missing teeth. INTERNATIONAL JOURNAL OF PROSTHODONTICS 2005.
     1. Reason: Not primary an oral health topic.
194. Joshi, AA; Gaikwad, AM; Padhye, AM; Nadgere, JB. Overview of Systematic Reviews and Meta-analyses Investigating the Efficacy of Different Nonsurgical Therapies for the Treatment of Peri-implant Diseases. INTERNATIONAL JOURNAL OF ORAL & MAXILLOFACIAL IMPLANTS 2022. http://dx.doi.org/10.11607/jomi.9088.
     1. Reason: Review of SRs.
195. Kairey, L; Leech, B; El-Assaad, F; Bugarcic, A; Dawson, D; Lauche, R. The effects of kefir consumption on human health: a systematic review of randomized controlled trials. NUTRITION REVIEWS 2023. http://dx.doi.org/10.1093/nutrit/nuac054.
     1. Reason: Not primary an oral health topic.
196. Kakde, S; Bhopal, RS; Jones, CM. A systematic review on the social context of smokeless tobacco use in the South Asian population: Implications for public health. PUBLIC HEALTH 2012. http://dx.doi.org/10.1016/j.puhe.2012.05.002.
     1. Reason: Not primary an oral health topic.
197. Kakudate, N; Morita, M; Sugai, M; Kawanami, M. Systematic cognitive behavioral approach for oral hygiene instruction: A short-term study. PATIENT EDUCATION AND COUNSELING 2009. http://dx.doi.org/10.1016/j.pec.2008.08.014.
     1. Reason: Not a SR.
198. Kanzow, P; Buttcher, AF; Wiegand, A; Schwendicke, F. Quality of Information Regarding Repair Restorations on Dentist Websites: Systematic Search and Analysis. JOURNAL OF MEDICAL INTERNET RESEARCH 2020. http://dx.doi.org/10.2196/17250.
     1. Reason: Not a SR.
199. Kao, RT; Nares, S; Reynolds, MA. Periodontal Regeneration - Intrabony Defects: A Systematic Review From the AAP Regeneration Workshop. JOURNAL OF PERIODONTOLOGY 2015. http://dx.doi.org/10.1902/jop.2015.130685.
     1. Reason: Unclear whether only primary studies were included the in assessment.
200. Kassebaum, NJ; Smith, AGC; Bernabe, E; Fleming, TD; Reynolds, AE; Vos, T; Murray, CJL; Marcenes, W. Global, Regional, and National Prevalence, Incidence, and Disability-Adjusted Life Years for Oral Conditions for 195 Countries, 1990-2015: A Systematic Analysis for the Global Burden of Diseases, Injuries, and Risk Factors. JOURNAL OF DENTAL RESEARCH 2017. http://dx.doi.org/10.1177/0022034517693566.
     1. Reason: Not a SR.
201. Khanna, SS; Dhaimade, PA. Green dentistry: a systematic review of ecological dental practices. ENVIRONMENT DEVELOPMENT AND SUSTAINABILITY 2019. http://dx.doi.org/10.1007/s10668-018-0156-5.
     1. Reason: Primary studies and review(s) were included in the assessment.
202. Kim, DM; Neiva, R. Periodontal Soft Tissue Non-Root Coverage Procedures: A Systematic Review From the AAP Regeneration Workshop. JOURNAL OF PERIODONTOLOGY 2015. http://dx.doi.org/10.1902/jop.2015.130684.
     1. Reason: Unclear whether only primary studies were included the in assessment.
203. Kiriakou, J; Pandis, N; Fleming, PS; Madianos, P; Polychronopoulou, A. Reporting quality of systematic review abstracts in leading oral implantology journals. JOURNAL OF DENTISTRY 2013. http://dx.doi.org/10.1016/j.jdent.2013.09.006.
     1. Reason: Not a SR.
204. Kirihara, S. Systematic Compounding of Ceramic Pastes in Stereolithographic Additive Manufacturing. MATERIALS 2021. http://dx.doi.org/10.3390/ma14227090.
     1. Reason: Not a SR.
205. Kirkevang, LL; El Karim, IA; Duncan, HF; Nagendrababu, V; Kruse, C. Outcomes reporting in systematic reviews on non-surgical root canal treatment: A scoping review for the development of a core outcome set. INTERNATIONAL ENDODONTIC JOURNAL 2022. http://dx.doi.org/10.1111/iej.13812.
     1. Reason: Not a SR.
206. Kiuchi, Y. Systematic and Stepwise Interprofessional Education in Showa University. YAKUGAKU ZASSHI-JOURNAL OF THE PHARMACEUTICAL SOCIETY OF JAPAN 2017. http://dx.doi.org/10.1248/yakushi.17-00003-2
     1. Reason: Not primary an oral health topic.
207. Klein, J; von dem Knesebeck, O. Inequalities in health care utilization among migrants and non-migrants in Germany: a systematic review. INTERNATIONAL JOURNAL FOR EQUITY IN HEALTH 2018. http://dx.doi.org/10.1186/s12939-018-0876-z.
     1. Reason: Not primary an oral health topic.
208. Klinge, A; Tranaeus, S; Becktor, J; Winitsky, N; Naimi-Akbar, A. The risk for infraposition of dental implants and ankylosed teeth in the anterior maxilla related to craniofacial growth, a systematic review. ACTA ODONTOLOGICA SCANDINAVICA 2021. http://dx.doi.org/10.1080/00016357.2020.1807046.
     1. Reason: Unclear whether only primary studies were included the in assessment.
209. Koide, Y; Kataoka, Y; Hasegawa, T; Ota, E; Noma, H. Effect of systemic bisphosphonate administration on patients with periodontal disease: a systematic review and meta-analysis protocol. BMJ OPEN 2022. http://dx.doi.org/10.1136/bmjopen-2021-057768.
     1. Reason: Not a SR.
210. Koletsi, D; Fleming, PS; Eliades, T; Pandis, N. The evidence from systematic reviews and meta-analyses published in orthodontic literature. Where do we stand?. EUROPEAN JOURNAL OF ORTHODONTICS 2015. http://dx.doi.org/10.1093/ejo/cju087.
     1. Reason: Not a SR.
211. Koletsi, D; Valla, K; Fleming, PS; Chaimani, A; Pandis, N. Assessment of publication bias required improvement in oral health systematic reviews. JOURNAL OF CLINICAL EPIDEMIOLOGY 2016. http://dx.doi.org/10.1016/j.jclinepi.2016.02.019.
     1. Reason: Not a SR.
212. Koole, S; De Bruyn, H. Contemporary undergraduate implant dentistry education: a systematic review. EUROPEAN JOURNAL OF DENTAL EDUCATION 2014. http://dx.doi.org/10.1111/eje.12076.
     1. Reason: Not primary an oral health topic.
213. Kullman, L; Al Sane, M. Guidelines for dental radiography immediately after a dento-alveolar trauma, a systematic literature review. DENTAL TRAUMATOLOGY 2012. http://dx.doi.org/10.1111/j.1600-9657.2011.01099.x.
     1. Reason: Unclear whether only primary studies were included the in assessment.
214. Kumar, SS; Swaminathan, A; Abdel-Daim, MM; Mohideen, SS. A systematic review on the effects of acrylamide and bisphenol A on the development of Drosophila melanogaster. MOLECULAR BIOLOGY REPORTS. <http://dx.doi.org/10.1007/s11033-022-07642-4>.
     1. Reason: Not primary an oral health topic.
215. Kunutsor, SK; Gillatt, D; Blom, AW. Systematic review of the safety and efficacy of osseointegration prosthesis after limb amputation. BRITISH JOURNAL OF SURGERY 2018. http://dx.doi.org/10.1002/bjs.11005.
     1. Reason: Not primary an oral health topic.
216. Lago, ADN; Cordon, R; Goncalves, LM; Menezes, CFS; Furtado, GS; Rodrigues, FCN; Marques, DMC. How to use laser safely in times of COVID-19: Systematic review. SPECIAL CARE IN DENTISTRY 2021 http://dx.doi.org/10.1111/scd.12593.
     1. Reason: Not primary an oral health topic.
217. Lahti, SM; Hausen, HW; Vaskilampi, T. The perceptions of users about barriers to the use of free systematic oral care among Finnish pre-school children - a qualitative study. ACTA ODONTOLOGICA SCANDINAVICA 1999. https://doi.org/10.1080/000163599428869.
     1. Reason: Not a SR.
218. Lauka, L; Brunetti, F; Beghdadi, N; Notarnicola, M; Sommacale, D; de'Angelis, N. Advantages of robotic right colectomy over laparoscopic right colectomy beyond the learning curve: a systematic review and meta-analysis. ANNALS OF LAPAROSCOPIC AND ENDOSCOPIC SURGERY 2020. http://dx.doi.org/10.21037/ales-20-36.
     1. Reason: Not primary an oral health topic.
219. Layton, D. A Critical Review of Search Strategies Used in Recent Systematic Reviews Published in Selected Prosthodontic and Implant-Related Journals: Are Systematic Reviews Actually Systematic?. INTERNATIONAL JOURNAL OF PROSTHODONTICS 2017. http://dx.doi.org/10.11607/ijp.5193.
     1. Reason: Not a SR.
220. Lee, OS; Ahn, S; Lee, YS. Effect and safety of early weight-bearing on the outcome after open-wedge high tibial osteotomy: a systematic review and meta-analysis. ARCHIVES OF ORTHOPAEDIC AND TRAUMA SURGERY 2017. http://dx.doi.org/10.1007/s00402-017-2703-1.
     1. Reason: Not primary an oral health topic.
221. Lei, Z; Zhang, WJ; Hua, J; Dong, W; Ning, W; Wang, YP. Effect and safety of Shengxuening (extract from excrement of bombyxin) for renal anemia: a systematic review. JOURNAL OF TRADITIONAL CHINESE MEDICINE 2016. http://dx.doi.org/10.1016/S0254-6272(16)30077-2.
     1. Reason: Not primary an oral health topic.
222. Leroy, R; Bourgeois, J; Verleye, L; Toma, S. Should systemic antibiotics be prescribed in periodontal abscesses and pericoronitis? A systematic review of the literature. EUROPEAN JOURNAL OF ORAL SCIENCES 2022. http://dx.doi.org/10.1111/eos.12884.
     1. Reason: Unclear whether only primary studies were included the in assessment.
223. Leroy, ZC; Wallin, R; Lee, S. The Role of School Health Services in Addressing the Needs of Students With Chronic Health Conditions: A Systematic Review. JOURNAL OF SCHOOL NURSING 2017. http://dx.doi.org/10.1177/1059840516678909.
     1. Reason: Not primary an oral health topic.
224. Leung, WK; Corbet, EF; Kan, KW; Lo, ECM; Liu, JKS. A regimen of systematic periodontal care after removal of impacted mandibular third molars manages periodontal pockets associated with the mandibular second molars. JOURNAL OF CLINICAL PERIODONTOLOGY 2005. http://dx.doi.org/10.1111/j.1600-051X.2005.00773.x.
     1. Reason: Not a SR.
225. Levi, L; Barak, S; Katz, J. Allergic reactions associated with metal alloys in porcelain-fused-to-metal fixed prosthodontic devices-A systematic review. QUINTESSENCE INTERNATIONAL 2012.
     1. Reason: Primary studies and review(s) were included in the assessment.
226. Levinson, J; Kohl, K; Baltag, V; Ross, DA. Investigating the effectiveness of school health services delivered by a health provider: A systematic review of systematic reviews. PLOS ONE 2019. http://dx.doi.org/10.1371/journal.pone.0212603.
     1. Reason: Not primary an oral health topic. Review of SRs.
227. Li, T; Hua, F; Dan, SQ; Zhong, YX; Levey, C; Song, YL. Reporting quality of systematic review abstracts in operative dentistry: An assessment using the PRISMA for Abstracts guidelines. JOURNAL OF DENTISTRY 2020. http://dx.doi.org/10.1016/j.jdent.2020.103471.
     1. Reason: Not a SR.
228. Li, Y; Yang, HA; Cao, J. Association between Alcohol Consumption and Cancers in the Chinese Population-A Systematic Review and Meta-Analysis. PLOS ONE 2011. http://dx.doi.org/10.1371/journal.pone.0018776.
     1. Reason: Not primary an oral health topic.
229. Lin, WS; Eckert, SE. Clinical performance of intentionally tilted implants versus axially positioned implants: A systematic review. CLINICAL ORAL IMPLANTS RESEARCH 2018. http://dx.doi.org/10.1111/clr.13294.
     1. Reason: Primary studies and review(s) were included in the assessment.
230. List, T; Axelsson, S. Management of TMD: evidence from systematic reviews and meta-analyses. JOURNAL OF ORAL REHABILITATION 2010. http://dx.doi.org/10.1111/j.1365-2842.2010.02089.x.
     1. Reason: Review of SRs.
231. Liu, F; Song, SP; Ye, X; Huang, SQ; He, J; Wang, G; Hu, XY. Oral health-related multiple outcomes of holistic health in elderly individuals: An umbrella review of systematic reviews and meta-analyses. FRONTIERS IN PUBLIC HEALTH 2022. http://dx.doi.org/10.3389/fpubh.2022.1021104.
     1. Reason: Review of SRs.
232. Loeb, MB; Becker, M; Eady, A; Walker-Dilks, C. Interventions to prevent aspiration pneumonia in older adults: A systematic review. JOURNAL OF THE AMERICAN GERIATRICS SOCIETY 2003. http://dx.doi.org/10.1046/j.1365-2389.2003.51318.x.
     1. Reason: Not primary an oral health topic.
233. Lopez-Lopez, J; Salas, EJ; Kustner, EC. Prognosis and treatment of dry mouth. Systematic review. MEDICINA CLINICA 2014. http://dx.doi.org/10.1016/j.medcli.2013.02.036.
     1. Reason: Primary studies and review(s) were included in the assessment.
234. Lopez, ER; Vazquez, LMR; Centelles, AV; Otero, FV; Otero, AIB; Fraga, CF; Centelles, PV. Impact of the systematic use of the informed consent form at public dental care units in Galicia (Spain). MEDICINA ORAL PATOLOGIA ORAL Y CIRUGIA BUCAL 2008.
     1. Reason: Not a SR.
235. Low, LF; Fletcher, J; Goodenough, B; Jeon, YH; Etherton-Beer, C; MacAndrew, M; Beattie, E. A Systematic Review of Interventions to Change Staff Care Practices in Order to Improve Resident Outcomes in Nursing Homes. PLOS ONE 2015. http://dx.doi.org/10.1371/journal.pone.0140711.
     1. Reason: Not primary an oral health topic.
236. Lukoff, D; Provenzano, R; Lu, F; Turner, R. Religious and spiritual case reports on MEDLINE: A systematic analysis of records from 1980 to 1996. ALTERNATIVE THERAPIES IN HEALTH AND MEDICINE 1999.
     1. Reason: Not primary an oral health topic. Not a SR.
237. MacDonald-Jankowski, DS; Dozier, MF. Systematic review in diagnostic radiology. DENTOMAXILLOFACIAL RADIOLOGY 2001. http://dx.doi.org/10.1038/sj.dmfr.4600586.
     1. Reason: Not a SR.
238. Machmud, PB; Djuwita, R; Gayatri, D; Khairani, N; Putra, WKY; Ronoatmodjo, S. Influence of Micronutrient Consumption by Tuberculosis Patients on the Sputum Conversion Rate: A Systematic Review and Meta-analysis Study. ACTA MEDICA INDONESIANA 2020.
     1. Reason: Not primary an oral health topic.
239. Madi, M; Hamzeh, H; Griffiths, M; Rushton, A; Heneghan, NR. Exploring taught masters education for healthcare practitioners: a systematic review of literature. BMC MEDICAL EDUCATION 2019. http://dx.doi.org/10.1186/s12909-019-1768-7.
     1. Reason: Not primary an oral health topic.
240. Mahasneh, SA; Horner, K; Cunliffe, J; Al-Salehi, S; Sengupta, A; AlHadidi, A. Guidelines on radiographic imaging as part of root canal treatment: a systematic review with a focus on review imaging after treatment. INTERNATIONAL ENDODONTIC JOURNAL 2018. http://dx.doi.org/10.1111/iej.12857.
     1. Reason: Unclear whether only primary studies were included the in assessment.
241. Mahri, M; Shen, N; Berrizbeitia, F; Rodan, R; Daer, A; Faigan, M; Taqi, D; Wu, KY; Ahmadi, M; Ducret, M; Emami, E; Tamimi, F. Osseointegration Pharmacology: A Systematic Mapping Using Artificial Intelligence. ACTA BIOMATERIALIA 2021. http://dx.doi.org/10.1016/j.actbio.2020.11.011.
     1. Reason: Not a SR.
242. Mallika, L; Augustine, D; Rao, RS; Patil, S; Alamir, AWH; Awan, KH; Sowmya, SV; Haragannavar, VC; Prasad, K. Does microbiome shift play a role in carcinogenesis? A systematic review. TRANSLATIONAL CANCER RESEARCH 2020. http://dx.doi.org/10.21037/tcr.2020.02.11.
     1. Reason: Not primary an oral health topic.
243. Mamikutty, R; Aly, AS; Marhazlinda, J. Databases Selection in a Systematic Review of the Association between Anthropometric Measurements and Dental Caries among Children in Asia. CHILDREN-BASEL 2021. http://dx.doi.org/10.3390/children8070565.
     1. Reason: Not a SR.
244. Manini, DR; Shega, FD; Guo, CF; Wang, YX. Role of Platelet-Rich Plasma in Spinal Fusion Surgery: Systematic Review and Meta-Analysis. ADVANCES IN ORTHOPEDICS 2020. http://dx.doi.org/10.1155/2020/8361798.
     1. Reason: Not primary an oral health topic.
245. Manna, S; Tripathy, S; Sah, RK; Padhi, BK; Kaur, S; Nowrouzi-Kia, B; Chattu, VK. The Burden of Non-Communicable Diseases (NCDs) among Prisoners in India: A Systematic Review and Meta-Analysis. HEALTHCARE 2022. http://dx.doi.org/10.3390/healthcare10102046.
     1. Reason: Not primary an oral health topic.
246. Marcenes, W; Kassebaum, NJ; Bernabe, E; Flaxman, A; Naghavi, M; Lopez, A; Murray, CJL. Global Burden of Oral Conditions in 1990-2010: A Systematic Analysis. JOURNAL OF DENTAL RESEARCH 2013. http://dx.doi.org/10.1177/0022034513490168.
     1. Reason: Not a SR.
247. Marconi, V; Iommi, M; Monachesi, C; Faragalli, A; Skrami, E; Gesuita, R; Ferrante, L; Carle, F.Validity of age estimation methods and reproducibility of bone/dental maturity indices for chronological age estimation: a systematic review and meta-analysis of validation studies. SCIENTIFIC REPORTS 2022. http://dx.doi.org/10.1038/s41598-022-19944-5.
     1. Reason: Not primary an oral health topic.
248. Marimon, X; Cerrolaza, M; Ferrer, M; Canto-Naves, O; Cabratosa-Termes, J; Perez, R. A Systematic Study of Restorative Crown-Materials Combinations for Dental Implants: Characterization of Mechanical Properties under Dynamic Loads. INTERNATIONAL JOURNAL OF MOLECULAR SCIENCES 2022. http://dx.doi.org/10.3390/ijms23158769.
     1. Reason: Not a SR.
249. Markkula, N; Cabieses, B; Lehti, V; Uphoff, E; Astorga, S; Stutzin, F. Use of health services among international migrant children - a systematic review. GLOBALIZATION AND HEALTH 2018. http://dx.doi.org/10.1186/s12992-018-0370-9.
     1. Reason: Not primary an oral health topic.
250. Marshall, BDL; Werb, D. Health outcomes associated with methamphetamine use among young people: a systematic review. ADDICTION 2010. http://dx.doi.org/10.1111/j.1360-0443.2010.02932.x.
     1. Reason: Not primary an oral health topic.
251. Marshman, Z; Gibson, BJ; Owens, J; Rodd, HD; Mazey, H; Baker, SR; Benson, PE; Robinson, PG. Seen but not heard: a systematic review of the place of the child in 21st-century dental research. INTERNATIONAL JOURNAL OF PAEDIATRIC DENTISTRY 2007. http://dx.doi.org/10.1111/j.1365-263X.2007.00845.x.
     1. Reason: Not primary an oral health topic.
252. Martelli, N; Serrano, C; van den Brink, H; Pineau, J; Prognon, P; Borget, I; El Batti, S. Advantages and disadvantages of 3-dimensional printing in surgery: A systematic review. SURGERY 2016. http://dx.doi.org/10.1016/j.surg.2015.12.017.
     1. Reason: Not primary an oral health topic.
253. Masterson, D; Martinez-Silveira, MS. Application of Peer Review of Electronic Search Strategies (PRESS) to assess the quality of systematic reviews search strategies. EM QUESTAO 2022. http://dx.doi.org/10.19132/1808-5245283.117865.
     1. Reason: Not primary oral health topic Not a SR.
254. Matei, A; Saccone, G; Vogel, JP; Armson, AB. Primary and secondary prevention of preterm birth: a review of systematic reviews and ongoing randomized controlled trials. EUROPEAN JOURNAL OF OBSTETRICS & GYNECOLOGY AND REPRODUCTIVE BIOLOGY 2019. http://dx.doi.org/10.1016/j.ejogrb.2018.12.022.
     1. Reason: Not primary an oral health topic. Review of SRs.
255. Maurer-Grubinger, C; Avaniadi, I; Adjami, F; Christian, W; Doerry, C; Fay, V; Fisch, V; Gerez, A; Goecke, J; Kaya, U; Keller, J; Kruger, D; Pflaum, J; Porsch, L; Wischnewski, C; Scharnweber, B; Sosnov, P; Oremek, G; Groneberg, DA; Ohlendorf, D. Systematic changes of the static upper body posture with a symmetric occlusion condition. BMC MUSCULOSKELETAL DISORDERS 2020 http://dx.doi.org/10.1186/s12891-020-03655-x.
     1. Reason: Not a SR.
256. Mc Gillicuddy, A; Kelly, M; Crean, AM; Sahm, LJ. The knowledge, attitudes and beliefs of patients and their healthcare professionals around oral dosage form modification: A systematic review of the qualitative literature. RESEARCH IN SOCIAL & ADMINISTRATIVE PHARMACY 2017. http://dx.doi.org/10.1016/j.sapharm.2016.09.004
     1. Reason: Not primary an oral health topic.
257. McDonald, H; Browne, J; Perruzza, J; Svarc, R; Davis, C; Adams, K; Palermo, C. Transformative effects of Aboriginal health placements for medical, nursing, and allied health students: A systematic review NURSING & HEALTH SCIENCES 2018. http://dx.doi.org/10.1111/nhs.12410.
     1. Reason: Not primary an oral health topic.
258. McEvoy, MP; Crilly, M; Young, T; Farrelly, J; Lewis, LK. How Comprehensively Is Evidence-Based Practice Represented in Australian Health Professional Accreditation Documents? A Systematic Audit. TEACHING AND LEARNING IN MEDICINE 2016. http://dx.doi.org/10.1080/10401334.2015.1107490.
     1. Reason: Not primary an oral health topic. Not a SR.
259. Mejare, IA; Klingberg, G; Mowafi, FK; Stecksen-Blicks, C; Twetman, SHA; Tranaeus, SH. A Systematic Map of Systematic Reviews in Pediatric Dentistry-What Do We Really Know?. PLOS ONE 2015. http://dx.doi.org/10.1371/journal.pone.0117537.
     1. Reason: Not a SR.
260. Melo, G; Duarte, J; Pauletto, P; Porporatti, AL; Stuginski-Barbosa, J; Winocur, E; Flores-Mir, C; Canto, GD. Bruxism: An umbrella review of systematic reviews. JOURNAL OF ORAL REHABILITATION 2019. http://dx.doi.org/10.1111/joor.12801.
     1. Reason: Review of SRs.
261. Mendes, V; dos Santos, GO; Calasans-Maia, MD; Granjeiro, JM; Moraschini, V. Impact of bisphosphonate therapy on dental implant outcomes: An overview of systematic review evidence. INTERNATIONAL JOURNAL OF ORAL AND MAXILLOFACIAL SURGERY 2019. http://dx.doi.org/10.1016/j.ijom.2018.09.006.
     1. Reason: Review of SRs.
262. Meshkova, DT; Di Giacomo, P; Panti, F; D'Urso, A; Serritella, E; Di Paolo, C. Application of a Systematic Protocol in the Treatment of TMDs With Occlusal Appliances: Effectiveness and Efficiency in a Longitudinal Retrospective Study With Medium-Term Follow-Up. JOURNAL OF INTERNATIONAL SOCIETY OF PREVENTIVE AND COMMUNITY DENTISTRY 2019. http://dx.doi.org/10.4103/jispcd.JISPCD_106_19.
     1. Reason: Not a SR.
263. Messias, A; Nicolau, P; Guerra, F . Different Interventions for Rehabilitation of the Edentulous Maxilla with Implant-Supported Prostheses: An Overview of Systematic Reviews. INTERNATIONAL JOURNAL OF PROSTHODONTICS 2021. http://dx.doi.org/10.11607/ijp.7162.
     1. Reason: Review of SRs.
264. Miao, XC; Wang, DH; Xu, LY; Wang, J; Zeng, DL; Lin, SX; Huang, C; Liu, XY; Jiang, XQ. The response of human osteoblasts, epithelial cells, fibroblasts, macrophages and oral bacteria to nanostructured titanium surfaces: a systematic study. INTERNATIONAL JOURNAL OF NANOMEDICINE 2017. http://dx.doi.org/10.2147/IJN.S126760.
     1. Reason: Not primary an oral health topic. Not a SR.
265. Mickenautsch, S; Yengopal, V. Extent and quality of systematic review evidence related to minimum intervention in dentistry: essential oils, powered toothbrushes, triclosan, xylitol. INTERNATIONAL DENTAL JOURNAL 2011. http://dx.doi.org/10.1111/j.1875-595X.2011.00055.x.
     1. Reason: Not a SR.
266. Min, SN; Duangporn, D; Gao, SS; Detsomboonrat, P. Quality of the adaptation procedures and psychometric properties of the scale of oral health outcomes for 5-year-old children (SOHO-5): a systematic review. QUALITY OF LIFE RESEARCH. http://dx.doi.org/10.1007/s11136-022-03280-2.
     1. Reason: Not primary an oral health topic.
267. Mischo, J; Faidt, T; McMillan, RB; Dudek, J; Gunaratnam, G; Bayenat, P; Holtsch, A; Spengler, C; Muller, F; Hahl, H; Bischoff, M; Hannig, M; Jacobs, K. Hydroxyapatite Pellets as Versatile Model Surfaces for Systematic Adhesion Studies on Enamel: A Force Spectroscopy Case Study. ACS BIOMATERIALS SCIENCE & ENGINEERING 2022. http://dx.doi.org/10.1021/acsbiomaterials.1c00925.
     1. Reason: Not a SR.
268. Misra, SM; Holdstock, N; Baez, JC; Garcia, N; Guiterrez, A; Swamy, P; Lee, K; Bjugstad, A. Systematic review of former unaccompanied immigrant minors' access to healthcare services in the United States. JOURNAL OF PUBLIC HEALTH-HEIDELBERG 2022. http://dx.doi.org/10.1007/s10389-021-01652-5.
     1. Reason: Not primary an oral health topic.
269. Mittal, N; Goyal, M; Mittal, PK. Understanding and Appraising Systematic Reviews and Meta-Analysis. JOURNAL OF CLINICAL PEDIATRIC DENTISTRY 2017. http://dx.doi.org/10.17796/1053-4628-41.5.317.
     1. Reason: Not primary an oral health topic.
270. Mobasher, WA; Asiri, BM; Asiri, LA; Saad, LM; Meshawi, EA; Assiri, HA; AlAbdulqader, AH; Alhabedi, BA; Mortada, HH; Alharbi, MM. CLEFT LIP AND PALATE SURGICAL MANAGEMENT IN PEDIATRIC: SYSTEMATIC LITERATURE REVIEW. INDO AMERICAN JOURNAL OF PHARMACEUTICAL SCIENCES 2019. http://dx.doi.org/10.5281/zenodo.2529610.
     1. Reason: Not primary an oral health topic.
271. Mojtahedzadeh, M; Boojar, MMA; Habtemariam, S; Nabavi, SM; Najafi, A; Ghahremanian, A; Baktash, M; Aghaabdollahian, S; Sureda, A; Bagheri, M. Systematic review: Effectiveness of herbal oral care products on ventilator-associated pneumonia. PHYTOTHERAPY RESEARCH 2021. http://dx.doi.org/10.1002/ptr.7060.
     1. Reason: Not primary an oral health topic.
272. Monje, A; Pommer, B. The Concept of Platform Switching to Preserve Peri-implant Bone Level: Assessment of Methodologic Quality of Systematic Reviews. INTERNATIONAL JOURNAL OF ORAL & MAXILLOFACIAL IMPLANTS 2015. http://dx.doi.org/10.11607/jomi.4103.
     1. Reason: Not primary an oral health topic.
273. Monsarrat, P; Blaizot, A; Kemoun, P; Ravaud, P; Nabet, C; Sixou, M; Vergnes, JN. Clinical research activity in periodontal medicine: a systematic mapping of trial registers. JOURNAL OF CLINICAL PERIODONTOLOGY 2016. http://dx.doi.org/10.1111/jcpe.12534.
     1. Reason: Not a SR.
274. Morales, OA; Perdomo, B; Cassany, D; Izarra, E. The generic structure of Spanish systematic reviews in Dentistry. CIRCULO DE LINGUISTICA APLICADA A LA COMUNICACION 2020. http://dx.doi.org/10.5209/clac.70569.
     1. Reason: Not a SR.
275. Moraschini, V; Barboza, EDP. Quality assessment of systematic reviews on alveolar socket preservation. INTERNATIONAL JOURNAL OF ORAL AND MAXILLOFACIAL SURGERY 2016. http://dx.doi.org/10.1016/j.ijom.2016.03.010.
     1. Reason: Not a SR.
276. Moraschini, V; Luz, D; Velloso, G; Barboza, EDP. Quality assessment of systematic reviews of the significance of keratinized mucosa on implant health. INTERNATIONAL JOURNAL OF ORAL AND MAXILLOFACIAL SURGERY 2017. http://dx.doi.org/10.1016/j.ijom.2017.02.1274.
     1. Reason: Not a SR.
277. Moraschini, V; Mourao, CFDB; Machado, RCD; Nascimento, JRB; Javid, K; Calasans-Maia, MD; Cardarelli, A; Montemezzi, P; Calasans-Maia, JD. Does Platelet-Rich Fibrin Decrease Dimensional Changes and Improve Postoperative Comfort in Post-Extraction Sockets? An Overview of Systematic Reviews. APPLIED SCIENCES-BASEL 2020. http://dx.doi.org/10.3390/app10175750.
     1. Reason: Review of SRs.
278. Moszner, N; Salz, U; Zimmermann, J. Chemical aspects of self-etching enamel-dentin adhesives: A systematic review. DENTAL MATERIALS 2005. http://dx.doi.org/10.1016/j.dental.2005.05.001.
     1. Reason: Unclear whether only primary studies were included the in assessment.
279. Munoz, MG; Martin, MA; de Dios, JG. SYSTEMATIC REVIEW ABOUT DENTAL CARIES IN CHILDREN AND ADOLESCENTS WITH OBESITY AND/OR OVERWEIGHT. NUTRICION HOSPITALARIA 2013. http://dx.doi.org/10.3305/nh.2013.28.5.6674.
     1. Reason: Unclear whether only primary studies were included the in assessment.
280. Munshi, T; Heckman, CJ; Darlow, S. Association between tobacco waterpipe smoking and head and neck conditions A systematic review. JOURNAL OF THE AMERICAN DENTAL ASSOCIATION 2015. http://dx.doi.org/10.1016/j.adaj.2015.04.014.
     1. Reason: Not primary an oral health topic.
281. Nagendrababu, V; Abbott, PV; Boutsioukis, C; Duncan, HF; Faggion, CM; Kishen, A; Murray, PE; Pulikkotil, SJ; Dummer, PMH. Methodological quality assessment criteria for the evaluation of laboratory-based studies included in systematic reviews within the specialty of Endodontology: A development protocol. INTERNATIONAL ENDODONTIC JOURNAL 2022. http://dx.doi.org/10.1111/iej.13682.
     1. Reason: Not a SR.
282. Nagendrababu, V; Faggion, CM; Pulikkotil, SJ; Alatta, A; Dummer, PMH. Methodological assessment and overall confidence in the results of systematic reviews with network meta-analyses in Endodontics.INTERNATIONAL ENDODONTIC  JOURNAL 2022. http://dx.doi.org/10.1111/iej.13693.
     1. Reason: Not a SR.
283. Nagendrababu, V; Pulikkotil, SJ; Sultan, OS; Jayaraman, J; Peters, OA. Methodological and Reporting Quality of Systematic Reviews and Meta-analyses in Endodontics. JOURNAL OF ENDODONTICS 2018. http://dx.doi.org/10.1016/j.joen.2018.02.013.
     1. Reason: Not a SR.
284. Naimi-Akbar, A; Hultin, M; Klinge, A; Klinge, B; Tranaeus, S; Lund, B. Antibiotic prophylaxis in orthognathic surgery: A complex systematic review. PLOS ONE 2018. http://dx.doi.org/10.1371/journal.pone.0191161.
     1. Reason: Primary studies and review(s) were included in the assessment.
285. Nash, S; Arora, A. Interventions to improve health literacy among Aboriginal and Torres Strait Islander Peoples: a systematic review.BMC PUBLIC HEALTH 2021. http://dx.doi.org/10.1186/s12889-021-10278-x.
     1. Reason: Not primary an oral health topic.
286. Nath, S; Ju, XQ; Haag, DG; Kapellas, K; Santiago, PHR; Jamieson, L. Prevalence of dental caries among Indigenous populations compared to non-Indigenous populations: a quantitative systematic review protocol. JBI EVIDENCE SYNTHESIS 2021. http://dx.doi.org/10.11124/JBIES-20-00449.
     1. Reason: Unclear whether only primary studies were included the in assessment.
287. Natto, ZS; Hameedaldain, A. METHODOLOGICAL QUALITY ASSESSMENT OF META-ANALYSES AND SYSTEMATIC REVIEWS OF THE RELATIONSHIP BETWEEN PERIODONTAL AND SYSTEMIC DISEASES. JOURNAL OF EVIDENCE-BASED DENTAL PRACTICE 2019. http://dx.doi.org/10.1016/j.jebdp.2018.12.003.
     1. Reason: Not a SR.
288. Navabi, N; Shahravan, A; Haj-Esmaeilzadeh, E. A PRISMA assessment of reporting the quality of published dental systematic reviews in Iran, up to 2017.JOURNAL OF ORAL HEALTH AND ORAL EPIDEMIOLOGY 2018. http://dx.doi.org/10.22122/johoe.v7i4.413.
     1. Reason: Not a SR.
289. Nethan, ST; Sinha, DN; Chandan, K; Mehrotra, R. Smokeless tobacco cessation interventions: A systematic review. INDIAN JOURNAL OF MEDICAL RESEARCH 2018. http://dx.doi.org/10.4103/ijmr.IJMR_1983_17.
     1. Reason: Not primary an oral health topic.
290. Ni, XX; Yu, Y; Tian, T; Liu, L; Li, X; Li, FM; Xu, Y; Zhao, L.Acupuncture for patients with cancer-induced xerostomia: a systematic review protocol. BMJ OPEN 2019. http://dx.doi.org/10.1136/bmjopen-2019-031892.
     1. Reason: Unclear whether only primary studies were included the in assessment.
291. Nordstrom, M; Carlsson, P; Ericson, D; Hedenbjork-Lager, A; Petersson, GH. Common resilience factors among healthy individuals exposed to chronic adversity: a systematic review. ACTA ODONTOLOGICA SCANDINAVICA. http://dx.doi.org/10.1080/00016357.2022.2095021.
     1. Reason: Not primary an oral health topic.
292. Novak, I; Morgan, C; Fahey, M; Finch-Edmondson, M; Galea, C; Hines, A; Langdon, K; Mc Namara, M; Paton, MCB; Popat, H; Shore, B; Khamis, A; Stanton, E; [1] Finemore, OP; Tricks, A; te Velde, A; Dark, L; Morton, N; Badawi, N.State of the Evidence Traffic Lights 2019: Systematic Review of Interventions for Preventing and Treating Children with Cerebral Palsy. CURRENT NEUROLOGY AND NEUROSCIENCE REPORTS 2020. http://dx.doi.org/10.1007/s11910-020-1022-z.
     1. Reason: Not primary an oral health topic.
293. Nuryunarsih, D; Lewis, S; Langley, T. Health Risks of Kretek Cigarettes: A Systematic Review. NICOTINE & TOBACCO RESEARCH 2021. http://dx.doi.org/10.1093/ntr/ntab016.
     1. Reason: Not primary an oral health topic.
294. O'Keeffe, M; Kelly, M; O'Herlihy, E; O'Toole, PW; Kearney, PM; Timmons, S; O'Shea, E; Stanton, C; Hickson, M; Rolland, Y; Rosse, CS; Issanchou, S; Maitre, I; Stelmach-Mardas, M; Nagel, G; Flechtner-Mors, M; Wolters, M; Hebestreit, A; De Groot, LCPGM; van de Rest, O; Teh, R; Peyron, MA; Dardevet, D; Papet, I; Schindler, K; Streicher, M; Torbahn, G; Kiesswetter, E; Visser, M; Volkert, D; O'Connor, EM. Potentially modifiable determinants of malnutrition in older adults: A systematic review. CLINICAL NUTRITION 2019. http://dx.doi.org/10.1016/j.clnu.2018.12.007.
     1. Reason: Not primary an oral health topic.
295. Oen, M; Leknes, KN; Lund, B; Bunaes, DF. The efficacy of systemic antibiotics as an adjunct to surgical treatment of peri-implantitis: a systematic review. BMC ORAL HEALTH 2021. http://dx.doi.org/10.1186/s12903-021-02020-1.
     1. Reason: Primary studies and review(s) were included in the assessment.
296. Ogunsalu, C; Judy, K; Obiechina, A; Daisely, H; Bissoon, AK; Steigmann, M; Dosumu, O; Okojie, V; Akeredolu, P. Systematic Approach for the Propagation of Postgraduate Dental Implant Education in Developing Countries. IMPLANT DENTISTRY 2009. http://dx.doi.org/10.1097/ID.0b013e3181991096.
     1. Reason: Not a SR.
297. Ojha, J; Rawal, YB; Hornick, JL; Magliocca, K; Montgomery, DR; Foss, RD; Torske, KR; Accurso, B. Extra Nodal Rosai-Dorfman Disease Originating in the Nasal and Paranasal Complex and Gnathic Bones: A Systematic Analysis of Seven Cases and Review of Literature. HEAD & NECK PATHOLOGY 2020. http://dx.doi.org/10.1007/s12105-019-01056-8.
     1. Reason: Not primary an oral health topic.
298. Osborne, B; Larance, B; Ivers, R; Deane, FP; Robinson, LD; Kelly, PJ. Systematic review of guidelines for managing physical health during treatment for substance use disorders: Implications for the alcohol and other drug workforce. DRUG AND ALCOHOL REVIEW 2022. http://dx.doi.org/10.1111/dar.13504.
     1. Reason: Not primary an oral health topic.
299. Osterberg, M; Holmlund, A; Sunzel, B; Tranaeus, S; Twetman, S; Lund, B. KNOWLEDGE GAPS IN ORAL AND MAXILLOFACIAL SURGERY: A SYSTEMATIC MAPPING. INTERNATIONAL JOURNAL OF TECHNOLOGY ASSESSMENT IN HEALTH CARE 2017. http://dx.doi.org/10.1017/S026646231700023X.
     1. Reason: Not a SR.
300. Otzen, T; Manterola, C; Mora, M; Quiroz, G; Salazar, P; Garcia, N. Statements, Recommendations, Proposals, Guidelines, Checklists and Scales Available for Reporting Results in Biomedical Research and Quality of Conduct. A Systematic Review. INTERNATIONAL JOURNAL OF MORPHOLOGY 2020. DOI:10.4067/S0717-95022020000300774.
     1. Reason: Not primary an oral health topic.
301. Owston, H; Giannoudis, PV; Jones, E. Do skeletal muscle MSCs in humans contribute to bone repair? A systematic review. INJURY-INTERNATIONAL JOURNAL OF THE CARE OF THE INJURED 2016. http://dx.doi.org/10.1016/S0020-1383(16)30834-8.
     1. Reason: Not primary an oral health topic.
302. Ozhayat, EB; Gotfredsen, K; Elverdam, B; Owall, B. Patient-Generated Aspects in Oral Rehabilitation Decision Making. II. Comparison of an Individual Systematic Interview Method and the Oral Health Impact Profile. INTERNATIONAL JOURNAL OF PROSTHODONTICS 2010.
     1. Reason: Not a SR.
303. Ozhayat, EB; Gotfredsen, K; Elverdam, B; Owall, B. Comparison of an individual systematic interview method and the oral health impact profile. Responsiveness and ability of describing treatment effect of oral rehabilitation. JOURNAL OF ORAL REHABILITATION 2010. http://dx.doi.org/10.1111/j.1365-2842.2010.02093.x.
     1. Reason: Not a SR.
304. Pachito, DV; Latorraca, CDC; Riera, R. Efficacy of platelet-rich plasma for non-transfusion use: Overview of systematic reviews. INTERNATIONAL JOURNAL OF CLINICAL PRACTICE 2019. http://dx.doi.org/10.1111/ijcp.13402.
     1. Reason: Not primary an oral health topic.
305. Padhiary, S; Samal, D; Khandayataray, P; Murthy, MK. A systematic review report on tobacco products and its health issues in India. REVIEWS ON ENVIRONMENTAL HEALTH 2021. http://dx.doi.org/10.1515/reveh-2020-0037.
     1. Reason: Not primary an oral health topic.
306. Pandis, N. Randomized clinical trials (RCTs) and systematic reviews (SRs) in the context of evidence-based orthodontics (EBO). SEMINARS IN ORTHODONTICS 2013. http://dx.doi.org/10.1053/j.sodo.2013.03.004.
     1. Reason: Not a SR.
307. Pandis, N; Fleming, PS; Worthington, H; Dwan, K; Salanti, G. Discrepancies in Outcome Reporting Exist Between Protocols and Published Oral Health Cochrane Systematic Reviews. PLOS ONE 2015. http://dx.doi.org/10.1371/journal.pone.0137667.
     1. Reason: Not a SR.
308. Pandis, N; Fleming, PS; Worthington, H; Salanti, G. The Quality of the Evidence According to GRADE Is Predominantly Low or Very Low in Oral Health Systematic Reviews. PLOS ONE 2015. http://dx.doi.org/10.1371/journal.pone.0131644.
     1. Reason: Not a SR.
309. Papageorgiou, SN; Eliades, T. Evidence-based orthodontics: Too many systematic reviews, too few trials. JOURNAL OF ORTHODONTICS 2019. http://dx.doi.org/10.1177/1465312519842322.
     1. Reason: Not a SR.
310. Papageorgiou, SN; Papadopoulos, MA; Athanasiou, AE. Evaluation of methodology and quality characteristics of systematic reviews in orthodontics. ORTHODONTICS & CRANIOFACIAL RESEARCH 2011. http://dx.doi.org/10.1111/j.1601-6343.2011.01522.x.
     1. Reason: Not a SR.
311. Papi, P; Letizia, C; Pilloni, A; Petramala, L; Saracino, V; Rosella, D; Pompa, G. Peri-implant diseases and metabolic syndrome components: a systematic review. EUROPEAN REVIEW FOR MEDICAL AND PHARMACOLOGICAL SCIENCES 2018. 10.26355/eurrev_201802_14364.
     1. Reason: Not primary an oral health topic.
312. Patzelt, SBM; Bahat, O; Reynolds, MA; Strub, JR. The All-on-Four Treatment Concept: A Systematic Review. CLINICAL IMPLANT DENTISTRY AND RELATED RESEARCH 2014. http://dx.doi.org/10.1111/cid.12068.
     1. Reason: Unclear whether only primary studies were included the in assessment.
313. Pauletto, P; Ruales-Carrera, E; Mezzomo, LA; Stefani, CM; Taba, M; Goncalves, RB; Flores-Mir, C; Canto, GD. Clinical performance of short versus standard dental implants in vertically augmented bone: an overview of systematic reviews. CLINICAL ORAL INVESTIGATIONS 2021. http://dx.doi.org/10.1007/s00784-021-04095-0.
     1. Reason: Review of SRs.
314. Pelepenko, LE; Janini, ACP; Gomes, BPFA; de-Jesus-Soares, A; Marciano, MA. Effects of Bismuth Exposure on the Human Kidney-A Systematic Review. ANTIBIOTICS-BASEL 2022. http://dx.doi.org/10.3390/antibiotics11121741.
     1. Reason: Not primary an oral health topic.
315. Peng, X; Cheng, L; You, Y; Tang, CW; Ren, B; Li, YQ; Xu, X; Zhou, XD. Oral microbiota in human systematic diseases. INTERNATIONAL JOURNAL OF ORAL SCIENCE 2022. http://dx.doi.org/10.1038/s41368-022-00163-7.
     1. Reason: Not a SR.
316. Phillips, AC; Mackintosh, SF; Gibbs, C; Ng, L; Fryer, CE. A comparison of electronic and paper-based clinical skills assessment: Systematic review. MEDICAL TEACHER 2019. http://dx.doi.org/10.1080/0142159X.2019.1623387.
     1. Reason: Not primary an oral health topic.
317. Pichichero, ME; Casey, JR. Systematic review of factors contributing to penicillin treatment failure in Streptococcus pyogenes pharyngitis. OTOLARYNGOLOGY-HEAD AND NECK SURGERY 2007. http://dx.doi.org/10.1016/j.otohns.2007.07.033.
     1. Reason: Not primary an oral health topic.
318. Popelut, A; Valet, F; Fromentin, O; Thomas, A; Bouchard, P. Relationship between Sponsorship and Failure Rate of Dental Implants: A Systematic Approach. PLOS ONE 2010. http://dx.doi.org/10.1371/journal.pone.0010274.
     1. Reason: Not a SR.
319. Prasad, JB; Dhar, M. Risk of major cancers associated with various forms of tobacco use in India: a systematic review and meta-analysis. JOURNAL OF PUBLIC HEALTH-HEIDELBERG 2019. http://dx.doi.org/10.1007/s10389-018-0992-7.
     1. Reason: Not primary an oral health topic.
320. Praveen, G; Pasupuleti, MK; Penmetsa, GS; Nagisetti, H; Indukuri, SD; Akkaloori, A. Systematic reviews in dental research: A bibliometric analysis of contribution from Indian dentists during 1948-2022. JOURNAL OF INTERNATIONAL SOCIETY OF PREVENTIVE AND COMMUNITY DENTISTRY 2022. http://dx.doi.org/10.4103/jispcd.JISPCD_127_22.
     1. Reason: Not a SR.
321. Pulikkotil, SJ; Jayaraman, J; Nagendrababu, V. Quality of abstract of systematic reviews and meta-analyses in paediatric dentistry journals. EUROPEAN ARCHIVES OF PAEDIATRIC DENTISTRY 2019. http://dx.doi.org/10.1007/s40368-019-00432-w.
     1. Reason: Not a SR.
322. Qi, SC; Yan, YH; Luo, E; Hu, J. The development of dental informatics and dental information technology in China: A systematic study. JOURNAL OF DENTAL SCIENCES 2015. http://dx.doi.org/10.1016/j.jds.2014.07.003.
     1. Reason: Not a SR.
323. Qin, XF; Zi, H; Zeng, XJ. Changes in the global burden of untreated dental caries from 1990 to 2019: A systematic analysis for the Global Burden of Disease study. HELIYON 2022. http://dx.doi.org/10.1016/j.heliyon.2022.e10714.
     1. Reason: Not a SR.
324. Quinn, JB; Quinn, GD. A practical and systematic review of Weibull statistics for reporting strengths of dental materials. DENTAL MATERIALS 2010. http://dx.doi.org/10.1016/j.dental.2009.09.006.
     1. Reason: Not primary an oral health topic.
325. Ramos-Garcia, P; Gonzalez-Moles, MA; Warnakulasuriya, S. Oral cancer development in lichen planus and related conditions-3.0 evidence level: A systematic review of systematic reviews. ORAL DISEASES 2021. http://dx.doi.org/10.1111/odi.13812.
     1. Reason: Review of SRs.
326. Randhawa, V; Singh, AK; Acharya, V. A systematic approach to prioritize drug targets using machine learning, a molecular descriptor-based classification model, and high-throughput screening of plant derived molecules: a case study in oral cancer. MOLECULAR BIOSYSTEMS 2015. http://dx.doi.org/10.1039/c5mb00468c.
     1. Reason: Not a SR.
327. Ranjbar, M; Noudeh, GD; Hashemipour, MA; Mohamadzadeh, I. A systematic study and effect of PLA/Al2O3 nanoscaffolds as dental resins: mechanochemical properties. ARTIFICIAL CELLS NANOMEDICINE AND BIOTECHNOLOGY 2019. http://dx.doi.org/10.1080/21691401.2018.1548472.
     1. Reason: Not a SR.
328. Rasmussen, K; Belisario, JM; Wark, PA; Molina, JA; Loong, SL; Cotic, Z; Papachristou, N; Riboli-Sasco, E; Car, LT; Musulanov, EM; Kunz, H; Zhang, YF; George, PP; Heng, BH; Wheeler, EL; Al Shorbaji, N; Svab, I; Atun, R; Majeed, A; Car, J. Offline eLearning for undergraduates in health professions: A systematic review of the impact on knowledge, skills, attitudes and satisfaction. JOURNAL OF GLOBAL HEALTH 2014. http://dx.doi.org/10.7189/jogh.04.010405.
     1. Reason: Not primary an oral health topic.
329. Rees, EL; Hawarden, AW; Dent, G; Hays, R; Bates, J; Hassell, AB. Evidence regarding the utility of multiple mini-interview (MMI) for selection to undergraduate health programs: A BEME systematic review: BEME Guide No. 37. MEDICAL TEACHER 2016. http://dx.doi.org/10.3109/0142159X.2016.1158799.
     1. Reason: Not primary an oral health topic.
330. Reitano, E; Francone, E; Bona, E; Follenzi, A; Gentilli, S. Gut Microbiota Association with Diverticular Disease Pathogenesis and Progression: A Systematic Review. DIGESTIVE DISEASES AND SCIENCES. http://dx.doi.org/10.1007/s10620-022-07600-x.
     1. Reason: Not primary an oral health topic.
331. Ribeiro, LG; Antunes, LS; Kuchler, EC; Baratto, F; Kirschneck, C; Guimaraes, LS; Antunes, LAA. Impact of malocclusion treatments on Oral Health-Related Quality of Life: an overview of systematic reviews. CLINICAL ORAL INVESTIGATIONS 2023. http://dx.doi.org/10.1007/s00784-022-04837-8.
     1. Reason: Review of SRs.
332. Richards, DA; Hilli, A; Pentecost, C; Goodwin, VA; Frost, J. Fundamental nursing care: A systematic review of the evidence on the effect of nursing care interventions for nutrition, elimination, mobility and hygiene. JOURNAL OF CLINICAL NURSING 2018. http://dx.doi.org/10.1111/jocn.14150.
     1. Reason: Not primary an oral health topic.
333. Rodriguez-Merchan, EC. What does the Cochrane database of systematic reviews tell us about hemophilia? EXPERT REVIEW OF HEMATOLOGY. <http://dx.doi.org/10.1080/17474086.2019.1676718>.
     1. Reason: Not primary an oral health topic.
334. Roffi, A; Filardo, G; Kon, E; Marcacci, M. Does PRP enhance bone integration with grafts, graft substitutes, or implants? A systematic review. BMC MUSCULOSKELETAL DISORDERS 2013. http://dx.doi.org/10.1186/1471-2474-14-330.
     1. Reason: Not primary an oral health topic.
335. Rolo, D; Assuncao, R; Ventura, C; Alvito, P; Goncalves, L; Martins, C; Bettencourt, A; Jordan, P; Vital, N; Pereira, J; Pinto, F; Matos, P; Silva, MJ; Louro, H. Adverse Outcome Pathways Associated with the Ingestion of Titanium Dioxide Nanoparticles-A Systematic Review. NANOMATERIALS 2022. http://dx.doi.org/10.3390/nano12193275.
     1. Reason: Not primary an oral health topic.
336. Rosa, CDDD; Gomes, JMD; de Moraes, SLD; Lemos, CAA; Minatel, L; Limirio, JPJD; Pellizzer, EP. Does non-surgical periodontal treatment influence on rheumatoid arthritis? A systematic review and meta-analysis. SAUDI DENTAL JOURNAL 2021. http://dx.doi.org/10.1016/j.sdentj.2021.09.007.
     1. Reason: Not primary an oral health topic.
337. Rosen, A; Fors, U; Zary, N; Sejersen, R; Lund, B. A systematic approach to improve oral and maxillofacial surgery education. EUROPEAN JOURNAL OF DENTAL EDUCATION 2011. http://dx.doi.org/10.1111/j.1600-0579.2010.00661.x.
     1. Reason: Not a SR.
338. Russo, DS; Cinelli, F; Sarti, C; Giachetti, L. Adhesion to Zirconia: A Systematic Review of Current Conditioning Methods and Bonding Materials. DENTISTRY JOURNAL 2019. http://dx.doi.org/10.3390/dj7030074.
     1. Reason: Primary studies and review(s) were included in the assessment.
339. Sahm, BD; Botelho, AL; Agnelli, JAM; dos Reis, AC. Relation of physicochemical properties and accumulation of microorganisms in acrylic resins with antimicrobial properties: a systematic review. POLYMER BULLETIN. http://dx.doi.org/10.1007/s00289-022-04659-4.
     1. Reason: Not primary an oral health topic.
340. Saikia, A; Muthu, MS; Orenuga, OO; Mossey, P; Ousehal, L; Yan, S; Campodonico, M; England, R; Taylor, S; Sheeran, P. Systematic Review of Clinical Practice Guidelines for Oral Health in Children With Cleft Lip and Palate. CLEFT PALATE-CRANIOFACIAL JOURNAL 2022. http://dx.doi.org/10.1177/10556656211025189.
     1. Reason: Unclear whether only primary studies were included the in assessment.
341. Sailer, I; Makarov, NA; Thoma, DS; Zwahlen, M; Pjetursson, BE. All-ceramic or metal-ceramic tooth-supported fixed dental prostheses (FDPs)? A systematic review of the survival and complication rates. Part I: Single crowns (SCs). DENTAL MATERIALS 2015. http://dx.doi.org/10.1016/j.dental.2015.02.011.
     1. Reason: Unclear whether only primary studies were included the in assessment.
342. Saletta, JM; Garcia, JJ; Carames, JMM; Schliephake, H; Marques, DND. Quality assessment of systematic reviews on vertical bone regeneration. INTERNATIONAL JOURNAL OF ORAL AND MAXILLOFACIAL SURGERY 2019. http://dx.doi.org/10.1016/j.ijom.2018.07.014.
     1. Reason: Not a SR.
343. Salgado-Peralvo, AO; Mateos-Moreno, MV; Velasco-Ortega, E; Pena-Cardelles, JF; Kewalramani, N. Preventive antibiotic therapy in bone augmentation procedures in oral implantology: A systematic review. JOURNAL OF STOMATOLOGY ORAL AND MAXILLOFACIAL SURGERY 2022. http://dx.doi.org/10.1016/j.jormas.2021.01.011.
     1. Reason: Primary studies and review(s) were included in the assessment.
344. Salmos, J; Gerbi, MEMM; Braz, R; Andrade, ESS; Vasconcelos, BCE; Bessa-Nogueira, RV. Methodological quality of systematic reviews analyzing the use of laser therapy in restorative dentistry. LASERS IN MEDICAL SCIENCE 2010. http://dx.doi.org/10.1007/s10103-009-0733-9.
     1. Reason: Not a SR.
345. Saltaji, H; Cummings, GG; Armijo-Olivo, S; Major, MP; Amin, M; Major, PW; Hartling, L; Flores-Mir, C.A Descriptive Analysis of Oral Health Systematic Reviews Published 1991-2012: Cross Sectional Study. PLOS ONE2013. http://dx.doi.org/10.1371/journal.pone.0074545.
     1. Reason: Not a SR.
346. Saltaji, H; Ospina, MB; Armijo-Olivo, S; Agarwal, S; Cummings, GG; Amin, M; Flores-Mir, C. Evaluation of risk of bias assessment of trials in systematic reviews of oral health interventions, 1991-2014 A methodology study.JOURNAL OF THE AMERICAN DENTAL ASSOCIATION 2016. http://dx.doi.org/10.1016/j.adaj.2016.03.017.
     1. Reason: Not a SR.
347. Sanchez-Torres, A; Sanchez-Garces, MA; Gay-Escoda, C. Materials and prognostic factors of bone regeneration in periapical surgery: A systematic review. MEDICINA ORAL PATOLOGIA ORAL Y CIRUGIA BUCAL 2014. http://dx.doi.org/10.4317/medoral.19453.
     1. Reason: Primary studies and review(s) were included in the assessment.
348. Santana, MAC; Cano, JAA; Kanan, AD; Velazquez, FAD; Munguia, PDS; Castanon, GAM; Silva, BEC; Valencia, CS; Orozco, MFS. Should We Be Concerned about the Association of Diabetes Mellitus and Periodontal Disease in the Risk of Infection by SARS-CoV-2? A Systematic Review and Hypothesis. MEDICINA-LITHUANIA 2021. http://dx.doi.org/10.3390/medicina57050493.
     1. Reason: Primary studies and review(s) were included in the assessment.
349. Santiago, JF; Lemos, CAA; Gomes, JMD; Verri, FR; Moraes, SLD; Pellizzer, EP. Quality Assessment of Systematic Reviews on Platform-Switching vs Platform-Matched Implants: An Overview. JOURNAL OF ORAL IMPLANTOLOGY 2020. http://dx.doi.org/10.1563/aaid-joi-D-19-00114.
     1. Reason: Not a SR.
350. Sarkies, MN; White, J; Henderson, K; Haas, R; Bowles, J. Additional weekend allied health services reduce length of stay in subacute rehabilitation wards but their effectiveness and cost-effectiveness are unclear in acute general medical and surgical hospital wards: a systematic review. JOURNAL OF PHYSIOTHERAPY 2018. http://dx.doi.org/10.1016/j.jphys.2018.05.004.
     1. Reason: Not primary an oral health topic.
351. Sarkis-Onofre, R; Pereira-Cenci, T; Tricco, AC; Demarco, FF; Moher, D; Cenci, MS. Systematic reviews in restorative dentistry: discussing relevant aspects. JOURNAL OF ESTHETIC AND RESTORATIVE DENTISTRY 2019. http://dx.doi.org/10.1111/jerd.12463.
     1. Reason: Not a SR.
352. Sartawi, SY; Abu-Hammad, S; Salim, NA; Al-Omoush, S. Denture Stomatitis Revisited: A Summary of Systematic Reviews in the Past Decade and Two Case Reports of Papillary Hyperplasia of Unusual Locations. INTERNATIONAL JOURNAL OF DENTISTRY 2021. http://dx.doi.org/10.1155/2021/7338143.
     1. Reason: Review of SRs.
353. Sartori, M; Maglio, M; Tschon, M; Aldini, NN; Visani, A; Fini, M. Functionalization of Ceramic Coatings for Enhancing Integration in Osteoporotic Bone: A Systematic Review. COATINGS 2019. http://dx.doi.org/10.3390/coatings9050312.
     1. Reason: Not primary an oral health topic.
354. Satheeshkumar, PS; Papatheodorou, S; Sonis, S. Enhanced oral hygiene interventions as a risk mitigation strategy for the prevention of non-ventilator-associated pneumonia: a systematic review and meta-analysis. BRITISH DENTAL JOURNAL 2020. http://dx.doi.org/10.1038/s41415-020-1452-7.
     1. Reason: Not primary an oral health topic.
355. Saulle, R; Sinopoli, A; Baer, AD; Mannocci, A; Marino, M; de Belvis, AG; Federici, A; La Torre, G. The PRECEDE-PROCEED model as a tool in Public Health screening: a systematic review. CLINICA TERAPEUTICA. <http://dx.doi.org/10.7417/CT2020.2208>
     1. Reason: Not primary an oral health topic.
356. Saxena, S; Singh, PK; Singh, L; Kashyap, S; Singh, S. Smokeless tobacco use and public health nutrition: a global systematic review. PUBLIC HEALTH NUTRITION 2023. http://dx.doi.org/10.1017/S1368980022001331.
     1. Reason: Not primary an oral health topic.
357. Schvaneveldt, N; Stellrecht, EM. Assessing the roles and challenges of librarians in dental systematic and scoping reviews. JOURNAL OF THE MEDICAL LIBRARY ASSOCIATION 2021. http://dx.doi.org/10.5195/jmla.2021.1031.
     1. Reason: Not a SR.
358. Seaman, CE; Green, E; Freire, KEffect of Rural Clinical Placements on Intention to Practice and Employment in Rural Australia: A Systematic Review. INTERNATIONAL JOURNAL OF ENVIRONMENTAL RESEARCH AND PUBLIC HEALTH 2022. http://dx.doi.org/10.3390/ijerph19095363.
     1. Reason: Not primary an oral health topic.
359. Seehra, J; Fleming, PS; Polychronopoulou, A; Pandis, N. Reporting completeness of abstracts of systematic reviews published in leading dental specialty journals. EUROPEAN JOURNAL OF ORAL SCIENCES 2013. http://dx.doi.org/10.1111/eos.12027.
     1. Reason: Not a SR.
360. Shah, PK; El Karim, I; Duncan, HF; Nagendrababu, V; Chong, BS. Outcomes reporting in systematic reviews on surgical endodontics: A scoping review for the development of a core outcome set. INTERNATIONAL ENDODONTIC JOURNAL 2022. http://dx.doi.org/10.1111/iej.13763.
     1. Reason: Not a SR.
361. Sharka, R; Abed, H; Hector, M. Oral health-related quality of life and satisfaction of edentulous patients using conventional complete dentures and implant-retained overdentures: An umbrella systematic review. GERODONTOLOGY 2019. http://dx.doi.org/10.1111/ger.12399.
     1. Reason: Review of SRs.
362. Shoaee, S; Moghaddam, SS; Masinaei, M; Sofi-Mahmudi, A; Hessari, H; Heydari, MH; Shamsoddin, E; Parsaeian, M; Ghasemian, A; Larijani, B; Fakhrzadeh, H; Farzadfar, F. Trends in dental caries of deciduous teeth in Iran: a systematic analysis of the national and sub-national data from 1990 to 2017. BMC ORAL HEALTH 2022. http://dx.doi.org/10.1186/s12903-022-02634-z.
     1. Reason: Not a SR.
363. Shyagali, TR; Rathore, A; Kapoor, S; Gupta, A; Tiwari, A; Patidar, R. Evaluation of exaggerated claims in the abstracts of systematic reviews reporting accelerated orthodontic tooth movement: a meta research analysis. AUSTRALASIAN ORTHODONTIC JOURNAL 2022. http://dx.doi.org/10.2478/aoj-2022-0028.
     1. Reason: Not a SR.
364. Sicilia, A; Noguerol, B; Cobo, J; Zabalegui, I. Profile surgical template: A systematic approach to precise implant placement. A technical note. INTERNATIONAL JOURNAL OF ORAL & MAXILLOFACIAL IMPLANTS 1998.
     1. Reason: Not a SR.
365. Sideri, S; Papageorgiou, SN; Eliades, T. Registration in the international prospective register of systematic reviews (PROSPERO) of systematic review protocols was associated with increased review quality. JOURNAL OF CLINICAL EPIDEMIOLOGY 2018. http://dx.doi.org/10.1016/j.jclinepi.2018.01.003.
     1. Reason: Not a SR.
366. Simone, K; Ahmed, RA; Konkin, J; Campbell, S; Hartling, L; Oswald, AE. What are the features of targeted or system-wide initiatives that affect diversity in health professions trainees? A BEME systematic review: BEME Guide No. 50. MEDICAL TEACHER 2018. http://dx.doi.org/10.1080/0142159X.2018.1473562.
     1. Reason: Not primary an oral health topic.
367. Sinha, I; Jones, L; Smyth, RL; Williamson, PR. A systematic review of studies that aim to determine which outcomes to measure in clinical trials in children. PLOS MEDICINE  2008. http://dx.doi.org/10.1371/journal.pmed.0050096.
     1. Reason: Not primary an oral health topic.
368. Slavish, DC; Szabo, YZ. The effect of acute stress on salivary markers of inflammation: a systematic review protocol. SYSTEMATIC REVIEWS 2019. http://dx.doi.org/10.1186/s13643-019-1026-4.
     1. Reason: Unclear whether only primary studies were included the in assessment.
369. Smail-Faugeron, V; Fron-Chabouis, H; Courson, F. Methodological quality and implications for practice of systematic Cochrane reviews in pediatric oral health: a critical assessment. BMC ORAL HEALTH 2014. http://dx.doi.org/10.1186/1472-6831-14-35.
     1. Reason: Not a SR.
370. Smith, SMS; Sonego, S; Wallen, GR; Waterer, G; Cheng, AC; Thompson, P. Use of non-pharmaceutical interventions to reduce the transmission of influenza in adults: A systematic review RESPIROLOGY 2015. http://dx.doi.org/10.1111/resp.12541.
     1. Reason: Not primary an oral health topic.
371. Soares, RC; da Rosa, SV; Moyses, ST; Rocha, JS; Bettega, PVC; Werneck, RI; Moyses, SJ. Methods for prevention of early childhood caries: Overview of systematic reviews.INTERNATIONAL JOURNAL OF PAEDIATRIC DENTISTRY 2021. http://dx.doi.org/10.1111/ipd.12766.
     1. Reason: Review of SRs.
372. Sofi-Mahmudi, A; Iranparvar, P; Shakiba, M; Shamsoddin, E; Mohammad-Rahimi, H; Naseri, S; Motie, P; Tovani-Palone, MR; Mesgarpour, B. Quality Assessment of Studies Included in Cochrane Oral Health Systematic Reviews: A Meta-Research. INTERNATIONAL JOURNAL OF ENVIRONMENTAL RESEARCH AND PUBLIC HEALTH 2021. http://dx.doi.org/10.3390/ijerph18147284.
     1. Reason: Not a SR.
373. Sohn, WS; Ismail, AI; Tellez, M. Efficacy of educational interventions targeting primary care providers' practice behaviors: an overview of published systematic reviews. JOURNAL OF PUBLIC HEALTH DENTISTRY 2004. http://dx.doi.org/10.1111/j.1752-7325.2004.tb02747.x.
     1. Reason: Review of SRs.
374. Sojan, E; Rai, H; Karunakar, S; Shaila, M; Afnan, M; Raj, H. Role of Glut-1 in Tumor Progression and Prognosis in Oral Squamous Cell Carcinoma: A Systematic Review. ORAL & MAXILLOFACIAL PATHOLOGY JOURNAL 2023.
     1. Reason: Not primary an oral health topic.
375. Soley-Bori, M; Ashworth, M; Bisquera, A; Dodhia, H; Lynch, R; Wang, YZ; Fox-Rushby, J. Impact of multimorbidity on healthcare costs and utilisation: a systematic review of the UK literature. BRITISH JOURNAL OF GENERAL PRACTICE 2021. http://dx.doi.org/10.3399/bjgp20X713897.
     1. Reason: Not primary an oral health topic.
376. Solow, R. Systematic review versus structured critical analysis. CRANIO-THE JOURNAL OF CRANIOMANDIBULAR & SLEEP PRACTICE 2021. http://dx.doi.org/10.1080/08869634.2019.1614288.
     1. Reason: Not primary an oral health topic.
377. Souto-Maior, JR; Pellizzer, EP; Gomes, JMD; Lemos, CAA; Santiago, JF; Vasconcelos, BCD; de Moraes, SLD. Influence of Diabetes on the Survival Rate and Marginal Bone Loss of Dental Implants: An Overview of Systematic Reviews. JOURNAL OF ORAL IMPLANTOLOGY 2019. http://dx.doi.org/10.1563/aaid-joi-D-19-00087.
     1. Reason: Review of SRs.
378. Spece, H; Basgul, C; Andrews, CE; MacDonald, DW; Taheri, ML; Kurtz, SM. A systematic review of preclinical in vivo testing of 3D printed porous Ti6Al4V for orthopedic applications, part I: Animal models and bone ingrowth outcome measures. JOURNAL OF BIOMEDICAL MATERIALS RESEARCH PART B-APPLIED BIOMATERIALS 2021. http://dx.doi.org/10.1002/jbm.b.34803.
     1. Reason: Not primary an oral health topic.
379. Steegmans, PAJ; Bipat, S; Reynders, RAM. Seeking adverse effects in systematic reviews of orthodontic interventions: protocol for a cross-sectional study. SYSTEMATIC REVIEWS 2019. http://dx.doi.org/10.1186/s13643-019-1000-1.
     1. Reason: Not a SR.
380. Stefanini, R; Tufik, S; Soares, MCM; Haddad, FLM; Bittencourt, LRA; Santos-Silva, R; Gregorio, LC. Systematic Evaluation of the Upper Airway in the Adult Population of Sao Paulo, Brazil. OTOLARYNGOLOGY-HEAD AND NECK SURGERY 2012. http://dx.doi.org/10.1177/0194599811434256.
     1. Reason: Not primary an oral health topic. Not a SR.
381. Stjernfeldt, PE; Wardh, I; Trulsson, M; Irving, GF; Bostrom, AM. Methods for objectively assessing clinical masticatory performance: protocol for a systematic review. SYSTEMATIC REVIEWS 2017. http://dx.doi.org/10.1186/s13643-016-0403-5.
     1. Reason: Not a SR.
382. Subramaniam, J; Leelavathi, L. Professional assessment of impact of lifestyle in development of Periodontal Disease: A systematic approach. INTERNATIONAL JOURNAL OF EARLY CHILDHOOD SPECIAL EDUCATION 2022. http://dx.doi.org/10.9756/INT-JECSE/V14I2.156.
     1. Reason: Not a SR.
383. Suebnukarn, S; Ngamboonsirisingh, S; Rattanabanlang, A. A Systematic Evaluation of the Quality of Meta-analyses in Endodontics. JOURNAL OF ENDODONTICS 2010. http://dx.doi.org/10.1016/j.joen.2009.12.019.
     1. Reason: Not a SR.
384. Sueters, J; Groenman, FA; Bouman, MB; Roovers, JPW; de Vries, R; Smit, TH; Huirne, JAF. Tissue Engineering Neovagina for Vaginoplasty in Mayer-Rokitansky-Kuster-Hauser Syndrome and Gender Dysphoria Patients: A Systematic Review. TISSUE ENGINEERING PART B-REVIEWS 2023. http://dx.doi.org/10.1089/ten.teb.2022.0067.
     1. Reason: Not primary an oral health topic.
385. Sutherland, SE; Matthews, DC. Conducting systematic reviews and creating clinical practice guidelines in dentistry - Lessons learned. JOURNAL OF THE AMERICAN DENTAL ASSOCIATION 2004. http://dx.doi.org/10.14219/jada.archive.2004.0301.
     1. Reason: Not a SR.
386. Szudek, J; Taylor, SM. Systematic review of the platysma myocutaneous flap for head and neck reconstruction ARCHIVES OF OTOLARYNGOLOGY-HEAD & NECK SURGERY 2007. http://dx.doi.org/10.1001/archotol.133.7.655.
     1. Reason: Not primary an oral health topic.
387. Tackmann, E; Dettmer, S. Health-related quality of life in adult heart-transplant recipients-a systematic review. HERZ 2020. http://dx.doi.org/10.1007/s00059-018-4745-8.
     1. Reason: Not primary an oral health topic.
388. Tan, HY; Wong, KY; Othman, MHD; Kek, HY; Wahab, RA; Ern, GKP; Chong, WT; Lee, KQ. Current and potential approaches on assessing airflow and particle dispersion in healthcare facilities: a systematic review. ENVIRONMENTAL SCIENCE AND POLLUTION RESEARCH. 2022. http://dx.doi.org/10.1007/s11356-022-23407-9.
     1. Reason: Not primary an oral health topic.
389. Tang, JY; Marinkovich, MP; Lucas, E; Gorell, E; Chiou, A; Lu, Y; Gillon, J; Patel, D; Rudin, D. A systematic literature review of the disease burden in patients with recessive dystrophic epidermolysis bullosa. ORPHANET JOURNAL OF RARE DISEASES 2021. http://dx.doi.org/10.1186/s13023-021-01811-7.
     1. Reason: Not primary an oral health topic.
390. Teich, ST; Heima, M; Lang, L. Dental Students' Use of AMSTAR to Critically Appraise Systematic Reviews. JOURNAL OF DENTAL EDUCATION 2015.
     1. Reason: Not a SR.
391. Teich, ST; Lang, LA; Demko, CA. Characteristics of the Cochrane Oral Health Group Systematic Reviews. JOURNAL OF DENTAL EDUCATION 2015. 10.1002/j.0022-0337.2015.79.1.tb05851.x.
     1. Reason: Not a SR.
392. Teoh, L; Sloan, AJ; McCullough, MJ; Thompson, W. Measuring Antibiotic Stewardship Programmes and Initiatives: An Umbrella Review in Primary Care Medicine and a Systematic Review of Dentistry. ANTIBIOTICS-BASEL 2020. http://dx.doi.org/10.3390/antibiotics9090607.
     1. Reason: Review of SRs.
393. Terry, R; Hing, W; Orr, R; Milne, N. Do coursework summative assessments predict clinical performance? A systematic review. BMC MEDICAL EDUCATION 2017. http://dx.doi.org/10.1186/s12909-017-0878-3.
     1. Reason: Not primary an oral health topic.
394. Thankappan, K; Subramanian, S; Balasubramanian, D; Kuriakose, MA; Sankaranarayanan, R; Iyer, S. Cost-effectiveness of oral cancer screening approaches by visual examination: A systematic review. HEAD AND NECK-JOURNAL FOR THE SCIENCES AND SPECIALTIES OF THE HEAD AND NECK 2021. http://dx.doi.org/10.1002/hed.26816.
     1. Reason: Not primary an oral health topic.
395. The American Academy of Periodontology. Translating findings of systematic reviews into consensus statements on periodontal therapy. JOURNAL OF THE AMERICAN DENTAL ASSOCIATION 2004. https://doi.org/10.14219/jada.archive.2004.0368.
     1. Reason: Not a SR.
396. Theodoratou, E; Tzoulaki, I; Zgaga, L; Ioannidis, JPA. Vitamin D and multiple health outcomes: umbrella review of systematic reviews and meta-analyses of observational studies and randomised trials. BMJ-BRITISH MEDICALJOURNAL 2014. http://dx.doi.org/10.1136/bmj.g2035.
     1. Reason: Not primary an oral health topic. Review of SRs.
397. Thistlethwaite, JE; Davies, D; Ekeocha, S; Kidd, JM; MacDougall, C; Matthews, P; Purkis, J; Clay, D. The effectiveness of case-based learning in health professional education. A BEME systematic review: BEME Guide No. 23. MEDICAL TEACHER 2012. http://dx.doi.org/10.3109/0142159X.2012.680939.
     1. Reason: Not primary an oral health topic.
398. Thompson, W; Tonkin-Crine, S; Pavitt, SH; McEachan, RRC; Douglas, GVA; Aggarwal, VR; Sandoe, JAT. Factors associated with antibiotic prescribing for adults with acute conditions: an umbrella review across primary care and a systematic review focusing on primary dental care. JOURNAL OF ANTIMICROBIAL CHEMOTHERAPY 2019. http://dx.doi.org/10.1093/jac/dkz152.
     1. Reason: Review of SRs.
399. Thurzo, A; Urbanova, W; Novak, B; Czako, L; Siebert, T; Stano, P; Marekova, S; Fountoulaki, G; Kosnacova, H; Varga, I. Where Is the Artificial Intelligence Applied in Dentistry? Systematic Review and Literature Analysis. HEALTHCARE 2022. http://dx.doi.org/10.3390/healthcare10071269.
     1. Reason: Primary studies and review(s) were included in the assessment.
400. Ting, M; Craig, J; Balkin, BE; Suzuki, JB. Peri-implantitis: A Comprehensive Overview of Systematic Reviews. JOURNAL OF ORAL IMPLANTOLOGY 2018. http://dx.doi.org/10.1563/aaid-joi-D-16-00122.
     1. Reason: Review of SRs.
401. Ting, M; Faulkner, RJ; Donatelli, DP; Suzuki, JB. Tooth-to-Implant-Supported Fixed Partial Denture: A Comprehensive Overview of Systematic Reviews. IMPLANT DENTISTRY 2019. http://dx.doi.org/10.1097/ID.0000000000000901.
     1. Reason: Review of SRs.
402. Ting, M; Tenaglia, MS; Jones, GH; Suzuki, JB. Surgical and Patient Factors Affecting Marginal Bone Levels Around Dental Implants: A Comprehensive Overview of Systematic Reviews. IMPLANT DENTISTRY 2017. http://dx.doi.org/10.1097/ID.0000000000000565.
     1. Reason: Review of SRs.
403. Toews, I; Lohner, S; de Gaudry, DK; Sommer, H; Meerpohl, JJ. Association between intake of non-sugar sweeteners and health outcomes: systematic review and meta-analyses of randomised and non-randomised controlled trials and observational studies. BMJ-BRITISH MEDICAL JOURNAL 2019. http://dx.doi.org/10.1136/bmj.k4718.
     1. Reason: Not primary an oral health topic.
404. Togninalli, D; Antonarakis, GS; Schatz, JP. Condylar resorption following mandibular advancement or bimaxillary osteotomies: A systematic review of systematic reviews. JOURNAL OF STOMATOLOGY ORAL AND MAXILLOFACIAL SURGERY 2022. http://dx.doi.org/10.1016/j.jormas.2022.03.008.
     1. Reason: Review of SRs.
405. Tran, L; Tam, DNH; Elshafay, A; Dang, T; Hirayama, K; Huy, NT. Quality assessment tools used in systematic reviews of in vitro studies: A systematic review. BMC MEDICAL RESEARCH METHODOLOGY 2021. http://dx.doi.org/10.1186/s12874-021-01295-w.
     1. Reason: Not primary an oral health topic.
406. Tsigarida, A; Chochlidakis, K. A Comparison Between Fixed and Removable Mandibular Implant-Supported Full-Arch Prostheses: An Overview of Systematic Reviews. INTERNATIONAL JOURNAL OF PROSTHODONTICS 2021. http://dx.doi.org/10.11607/ijp.6911.
     1. Reason: Review of SRs.
407. Turner, E; Berry, K; Aggarwal, VR; Quinlivan, L; Villanueva, T; Palmier-Claus, J. Oral health self-care behaviours in serious mental illness: A systematic review and meta-analysis. ACTA PSYCHIATRICA SCANDINAVICA 2022. http://dx.doi.org/10.1111/acps.13308.
     1. Reason: Not primary an oral health topic.
408. URKEN, ML; MOSCOSO, JF; LAWSON, W; BILLER, HF. A SYSTEMATIC-APPROACH TO FUNCTIONAL RECONSTRUCTION OF THE ORAL CAVITY FOLLOWING PARTIAL AND TOTAL GLOSSECTOMY. ARCHIVES OF OTOLARYNGOLOGY-HEAD & NECK SURGERY 1994.10.1001/archotol.1994.01880300007002.
     1. Reason: Not a SR.
409. Uruthiralingam, U; Rea, PM.  Augmented and Virtual Reality in Anatomical Education - A Systematic Review. BIOMEDICAL VISUALISATION, VOL 6 2020. http://dx.doi.org/10.1007/978-3-030-37639-0_5.
     1. Reason: Not primary an oral health topic.
410. van Breeschoten, J; Lourenco, RD; Signorelli, C; Haas, M; Cohn, RJ; Wakefield, CE; Fardell, JE. Patterns and drivers of health care use in long-term childhood cancer survivors: A systematic review. CRITICAL REVIEWS IN ONCOLOGY HEMATOLOGY 2017. http://dx.doi.org/10.1016/j.critrevonc.2017.10.004.
     1. Reason: Not primary an oral health topic.
411. van den Borre, CE; Zigterman, BGR; Mommaerts, MY; Braem, A. How surface coatings on titanium implants affect keratinized tissue: A systematic review. JOURNAL OF BIOMEDICAL MATERIALS RESEARCH PART B-APPLIED BIOMATERIALS 2022. http://dx.doi.org/10.1002/jbm.b.35025.
     1. Reason: Not primary an oral health topic.
412. van der Maarel-Wierink, CD; Vanobbergen, JNO; Bronkhorst, EM; Schols, JMGA; de Baat, C. Risk Factors for Aspiration Pneumonia in Frail Older People: A Systematic Literature Review. JOURNAL OF THE AMERICAN MEDICAL DIRECTORS ASSOCIATION 2011. http://dx.doi.org/10.1016/j.jamda.2010.12.099.
     1. Reason: Not primary an oral health topic.
413. Vargas-Espinosa, ML; Sanmarti-Garcia, G; Vazquez-Delgado, E; Gay-Escoda, C. Antiepileptic drugs for the treatment of neuropathic pain: A systematic review. MEDICINA ORAL PATOLOGIA ORAL Y CIRUGIA BUCAL 2012. http://dx.doi.org/10.4317/medoral.18001.
     1. Reason: Not primary an oral health topic.
414. Vargas, MA; Margeas, R. A systematic approach to contouring and polishing anterior resin composite restorations: A checklist manifesto. JOURNAL OF ESTHETIC AND RESTORATIVE DENTISTRY 2021. http://dx.doi.org/10.1111/jerd.12698.
     1. Reason: Not primary a SR of primary studies
415. Vasconcellos, AFG; Palmier, NR; Ribeiro, ACP; Normando, AGC; Morais-Faria, K; Gomes-Silva, W; Vechiato, AJ; de Goes, MF; Leme, AFP; Brandao, TB; Lopes, MA; Marsh, PD; Santos-Silva, AR. Impact of Clustering Oral Symptoms in the Pathogenesis of Radiation Caries: A Systematic Review. CARIES RESEARCH 2020. http://dx.doi.org/10.1159/000504878.
     1. Reason: Not primary an oral health topic.
416. Vasquez-Cardenas, J; Zapata-Norena, O; Carvsjal-Florez, A; Barbosa-Liz, DM; Giannakopoulos, NN; Faggion, CL. Systematic reviews in orthodontics: Impact of the PRISMA for Abstracts checklist on completeness of reporting. AMERICAN JOURNAL OF ORTHODONTICS AND DENTOFACIAL ORTHOPEDICS 2019. http://dx.doi.org/10.1016/j.ajodo.2019.05.009.
     1. Reason: Not a SR.
417. Veale, D; Gledhill, LJ; Christodoulou, P; Hodsoll, J. Body dysmorphic disorder in different settings: A systematic review and estimated weighted prevalence. BODY IMAGE 2016. http://dx.doi.org/10.1016/j.bodyim.2016.07.003.
     1. Reason: Not primary an oral health topic.
418. Vercammen, KA; Frelier, JM; Lowery, CM; McGlone, ME; Ebbeling, CB; Bleich, SN. A systematic review of strategies to reduce sugar-sweetened beverage consumption among 0-year to 5-year olds. OBESITY REVIEWS 2018. http://dx.doi.org/10.1111/obr.12741.
     1. Reason: Not primary an oral health topic.
419. von Philipsborn, P; Stratil, JM; Burns, J; Busert, LK; Pfadenhauer, LM; Polus, S; Holzapfel, C; Hauner, H; Rehfuess, EA. Environmental Interventions to Reduce the Consumption of Sugar-Sweetened Beverages: Abridged Cochrane Systematic Review. OBESITY FACTS 2020. http://dx.doi.org/10.1159/000508843.
     1. Reason: Not primary an oral health topic.
420. Wallace, N; Schaffer, NE; Aleem, IS; Patel, R. 3D-printed Patient-specific Spine Implants A Systematic Review. CLINICAL SPINE SURGERY 2020. http://dx.doi.org/10.1097/BSD.0000000000001026.
     1. Reason: Not primary an oral health topic.
421. Wang, SY; Zhang, ZY; Xia, LG; Zhao, J; Sun, XJ; Zhang, XL; Ye, DX; Uludag, H; Jiang, XQ. Systematic evaluation of a tissue-engineered bone for maxillary sinus augmentation in large animal canine model. BONE 2010. http://dx.doi.org/10.1016/j.bone.2009.09.008
     1. Reason: Not a SR.
422. Wang, X; Jiang, DM; Li, TX; Zhang, X; Wang, R; Gao, S; Yang, FY; Wang, Y; Tian, Q; Xie, CR; Liang, JH. Association between microbiological risk factors and neurodegenerative disorders: An umbrella review of systematic reviews and meta-analyses. FRONTIERS IN PSYCHIATRY 2022. http://dx.doi.org/10.3389/fpsyt.2022.991085.
     1. Reason: Not primary an oral health topic. Review of SRs.
423. Wasfi, RA; Bang, F; de Groh, M; Champagne, A; Han, AR; Lang, JJ; McFaull, SR; Melvin, A; Pipe, AL; Saxena, S; Thompson, W; Warner, E; Prince, SA. Chronic health effects associated with electronic cigarette use: A systematic review. FRONTIERS IN PUBLIC HEALTH 2022. http://dx.doi.org/10.3389/fpubh.2022.959622.
     1. Reason: Not primary an oral health topic.
424. Wasiak, J; Shen, AY; Tan, HB; Mahar, R; Kan, G; Khoo, WR; Faggion, CM. Methodological quality assessment of paper-based systematic reviews published in oral health. CLINICAL ORAL INVESTIGATIONS 2016. http://dx.doi.org/10.1007/s00784-015-1663-5.
     1. Reason: Not a SR.
425. Waziry, R; Jawad, M; Ballout, RA; Al Akel, M; Akl, EA. The effects of waterpipe tobacco smoking on health outcomes: an updated systematic review and meta-analysis. INTERNATIONAL JOURNAL OF EPIDEMIOLOGY 2017. http://dx.doi.org/10.1093/ije/dyw021.
     1. Reason: Not primary an oral health topic.
426. Weisfeld, CC; Turner, JA; Dunleavy, K; Ko, A; Bowen, JI; Roelk, B; Eissa, R; Benfield, E; Robertson, K. Dealing with Anxious Patients: A Systematic Review of the Literature on Nonpharmaceutical Interventions to Reduce Anxiety in Patients Undergoing Medical or Dental Procedures. JOURNAL OF ALTERNATIVE AND COMPLEMENTARY MEDICINE 2021. http://dx.doi.org/10.1089/acm.2020.0504.
     1. Reason: Not primary an oral health topic.
427. Williams, PG. The Benefits of Breakfast Cereal Consumption: A Systematic Review of the Evidence Base. ADVANCES IN NUTRITION 2014. http://dx.doi.org/10.3945/an.114.006247.
     1. Reason: Not primary an oral health topic.
428. Wilson, C; Freitas, CMT; Awan, KH; Ajdaharian, J; Geiler, J; Thirucenthilvelan, P. Adverse effects of E-cigarettes on head, neck, and oral cells: A systematic review. JOURNAL OF ORAL PATHOLOGY & MEDICINE. <http://dx.doi.org/10.1111/jop.13273>.
     1. Reason: Not primary an oral health topic.
429. Wong, LB; Yap, AU; Allen, PF.Periodontal disease and quality of life: Umbrella review of systematic reviews. JOURNAL OF PERIODONTAL RESEARCH 2021. http://dx.doi.org/10.1111/jre.12805.
     1. Reason: Review of SRs.
430. Wu, JQ; Feng, HJ; Ouyang, W; Sun, YG; Chen, P; Wang, J; Xian, JL; Huang, LH. Systematic evaluation of salivary gland damage following I-131 therapy in differentiated thyroid cancer patients by quantitative scintigraphy and clinical follow-up. NUCLEAR MEDICINE COMMUNICATIONS 2015. http://dx.doi.org/10.1097/MNM.0000000000000325.
     1. Reason: Not a SR.
431. Wu, LT; Low, MMJ; Tan, KK; Lopez, V; Liaw, SY. Why not nursing? A systematic review of factors influencing career choice among healthcare students. INTERNATIONAL NURSING REVIEW 2015. http://dx.doi.org/10.1111/inr.12220.
     1. Reason: Not primary an oral health topic.
432. Wu, QJ; Wang, X; Jin, D; Zhang, ZS; Jiang, F; Wen, J; Jiang, XQ. Validation of Digital Evaluation in Systematic Training on Tooth Preparation in Aesthetic Veneer Rehabilitation. CHINESE JOURNAL OF DENTAL RESEARCH 2021. http://dx.doi.org/10.3290/j.cjdr.b1105879.
     1. Reason: Not a SR.
433. Wu, ZX; Cai, MJ; Huang, PD; Chen, JY; Lv, ZH; Huang, XY. Comparative efficacy and dysmenorrhea score of 6 object-separated moxibustions for the treatment of Chinese patients with dysmenorrhea A systematic review and network meta-analysis. MEDICINE 2021. http://dx.doi.org/10.1097/MD.0000000000026185.
     1. Reason: Not primary an oral health topic.
434. Xiao, PL; Hsu, CJ; Ma, YG; Liu, D; Peng, R; Xu, XH; Lu, HD. Prevalence and treatment rate of osteoporosis in patients undergoing total knee and hip arthroplasty: a systematic review and meta-analysis. ARCHIVES OF OSTEOPOROSIS 2022. http://dx.doi.org/10.1007/s11657-021-01055-9.
     1. Reason: Not primary an oral health topic.
435. Xie, F; Teng, L; Jin, XL; Zheng, JL; Xu, JJ; Lu, JJ; Zhang, C; Xu, MB; Zeng, HF; Li, SY; Sun, XJ. Systematic Analysis of Clinical Outcomes of Anterior Maxillary and Mandibular Subapical Osteotomy With Preoperative Modeling in the Treatment of Bimaxillary Protrusion. JOURNAL OF CRANIOFACIAL SURGERY 2013. http://dx.doi.org/10.1097/SCS.0b013e3182a28b45
     1. Reason: Not a SR.
436. Yan, Q; Chen, P; Wang, ST; Liu, N; Zhao, P; Gu, AH. Association between HIF-1 alpha C1772T/G1790A polymorphisms and cancer susceptibility: an updated systematic review and meta-analysis based on 40 case-control studies. BMC CANCER 2014. http://dx.doi.org/10.1186/1471-2407-14-950.
     1. Reason: Not primary an oral health topic.
437. Yan, YZ; Wang, LJ; Si, ZH; Zhang, XY; Yuan, WK. A novel cocrystal of metformin hydrochloride with citric acid: Systematic synthesis and computational simulation. EUROPEAN JOURNAL OF PHARMACEUTICS AND BIOPHARMACEUTICS 2022. http://dx.doi.org/10.1016/j.ejpb.2022.08.013.
     1. Reason: Not primary an oral health topic. Not a SR.
438. Yaylali, IE; Alacam, T. Critical Assessment of Search Strategies in Systematic Reviews in Endodontics. JOURNAL OF ENDODONTICS 2016. http://dx.doi.org/10.1016/j.joen.2016.02.018.
     1. Reason: Not primary a SR of primary studies
439. Yi, J; Xiao, J; Li, H; Li, Y; Li, X; Zhao, Z. Effectiveness of adjunctive interventions for accelerating orthodontic tooth movement: a systematic review of systematic reviews. JOURNAL OF ORAL REHABILITATION 2017. http://dx.doi.org/10.1111/joor.12509.
     1. Reason: Review of SRs.
440. Yoshimura, HN; Chimanski, A; Cesar, PF. Systematic approach to preparing ceramic-glass composites with high translucency for dental restorations. DENTAL MATERIALS 2015. http://dx.doi.org/10.1016/j.dental.2015.06.015.
     1. Reason: Not a SR.
441. Yourtee, DM; Smith, RE; Russo, KA; Burmaster, S; Cannon, JM; Eick, JD; Kostoryz, EL. The stability of methacrylate biomaterials when enzyme challenged: Kinetic and systematic evaluations. JOURNAL OF BIOMEDICAL MATERIALS RESEARCH 2001. http://dx.doi.org/10.1002/1097-4636(20011215)57:4<522::AID-JBM1198>3.0.CO;2-9.
     1. Reason: Not a SR.
442. Zanirato, A; Cavagnaro, L; Basso, M; Divano, S; Felli, L; Formica, M. Metaphyseal sleeves in total knee arthroplasty revision: complications, clinical and radiological results. A systematic review of the literature. ARCHIVES OF ORTHOPAEDIC AND TRAUMA SURGERY 2018. http://dx.doi.org/10.1007/s00402-018-2967-0.
     1. Reason: Not primary an oral health topic.
443. Zhang, JD; Xu, JZ; Zhang, J; Ren, Y. Chinese herbal compound combined with western medicine therapy in the treatment of plasma cell mastitis A protocol for systematic review and meta-analysis. MEDICINE 2020. http://dx.doi.org/10.1097/MD.0000000000022858.
     1. Reason: Not primary an oral health topic.
444. Zhang, XA; Zhang, LF; Tan, XY; Lin, Y; Han, XS; Wang, HD; Ming, HW; Li, QJ; Liu, K; Feng, G. Systematic analysis of genes involved in oral cancer metastasis to lymph nodes. CELLULAR & MOLECULAR BIOLOGY LETTERS 2018. http://dx.doi.org/10.1186/s11658-018-0120-2.
     1. Reason: Not a SR.
445. Zhang, XM; Tian, XM; Wei, YZ; Deng, H; Ma, LC; Chen, ZY. Activity and Safety of Tegafur, Gimeracil, and Oteracil Potassium for Nasopharyngeal Carcinoma: A Systematic Review and Meta-Analysis. JOURNAL OF ONCOLOGY 2021. http://dx.doi.org/10.1155/2021/6690275.
     1. Reason: Not primary an oral health topic.
446. Zhang, Y; Sun, XY; Li, KX; Wang, XM; Cai, LJ; Li, X; Zhou, M. The Therapy of Elimination First for Early Acute Mastitis: A Systematic Review and Meta-Analysis. EVIDENCE-BASED COMPLEMENTARY AND ALTERNATIVE MEDICINE 2018. http://dx.doi.org/10.1155/2018/8059256.
     1. Reason: Not primary an oral health topic.
447. Zhang, YF; Xu, Q; Lu, J; Wang, P; Zhang, HW; Zhou, L; Ma, XQ; Zhou, YH. Tea consumption and the incidence of cancer: a systematic review and meta-analysis of prospective observational studies. EUROPEAN JOURNAL OF CANCER PREVENTION 2015. http://dx.doi.org/10.1097/CEJ.0000000000000094.
     1. Reason: Not primary an oral health topic.
448. Zhou, C; Ren, Y; Li, J; Li, X; He, JJ; Liu, PJ. Systematic review and meta-analysis of rectal washout on risk of local recurrence for cancer. JOURNAL OF SURGICAL RESEARCH 2014. http://dx.doi.org/10.1016/j.jss.2014.01.030.
     1. Reason: Not primary an oral health topic.
